# Linear Increments with Non‐monotone Missing Data and Measurement Error

**DOI:** 10.1111/sjos.12225

**Published:** 2016-04-06

**Authors:** Shaun R. Seaman, Daniel Farewell, Ian R. White

**Affiliations:** ^1^ Medical Research Council Biostatistics Unit; ^2^ Institute of Primary Care and Public HealthCardiff University

**Keywords:** ignorability, imputation, missing not at random, mortal cohort inference, non‐ignorable missing data, partly conditional inference

## Abstract

Linear increments (LI) are used to analyse repeated outcome data with missing values. Previously, two LI methods have been proposed, one allowing non‐monotone missingness but not independent measurement error and one allowing independent measurement error but only monotone missingness. In both, it was suggested that the expected increment could depend on current outcome. We show that LI can allow non‐monotone missingness and either independent measurement error of unknown variance or dependence of expected increment on current outcome but not both. A popular alternative to LI is a multivariate normal model ignoring the missingness pattern. This gives consistent estimation when data are normally distributed and missing at random (MAR). We clarify the relation between MAR and the assumptions of LI and show that for continuous outcomes multivariate normal estimators are also consistent under (non‐MAR and non‐normal) assumptions not much stronger than those of LI. Moreover, when missingness is non‐monotone, they are typically more efficient.

## 1 Introduction

Many medical studies involve repeated measurement of an outcome over time on a set of patients. Such longitudinal studies include clinical trials and observational cohort studies. Missing outcome data are a common feature of these studies, typically arising because patients miss scheduled visits or drop out of the study. Statistical methods for analysing such incomplete datasets include inverse probability weighting (Seaman & White, 2013), multiple imputation (Little & Rubin, 2002), random‐effects models (Verbeke & Molenberghs, 2000), shared random‐effects models (Henderson et al., 2000), doubly robust estimation (Seaman & Copas, 2009) and the focus of this article: linear increments (LI).

Linear increments were introduced by Diggle *et al.* (2007) (henceforth ‘DF&H’), who brought ideas from survival analysis into the analysis of longitudinal data. This has consequent advantages such as the ability to use martingale central limit theorems. LI was later developed by Aalen & Gunnes (2010) (henceforth ‘A&G’). Pullenayegum & Feldman (2013) extended the LI approach to allow for irregular observation times. Gunnes *et al.* (2009a) & Gunnes *et al.* (2009b) and Kingsley *et al.* (2012) describe applications of LI to randomized trials and observational studies.

DF&H assumed that the change in the underlying outcome (the ‘increment’) of a patient between two consecutive measurement times *t* − 1 and *t* is the sum of a predictable component and a martingale increment, and that these underlying outcomes are then measured with mutually independent errors that are independent of all the underlying outcomes and whose variances are unknown (‘independent measurement error’). A linear model is specified for the dependence of the predictable component on covariates, and the parameters of this model are estimated using a series of linear regressions. The expected outcome at each time *t* is then estimated by summing the predictable components up to time *t* and averaging over the individuals, a method known as estimating the compensator. DF&H's basic model assumes that the increment between times *t* − 1 and *t* is independent of the underlying outcome at time *t* − 1. However, they suggested that this assumption could be weakened by including the measured outcome (or function thereof) at time *t* − 1 as a covariate in the model for the predictable component of the increment. DF&H's method was limited to monotone missing data, that is, data where if a patient's outcome is missing at time *t* then it is also missing at all later times.

A&G allowed for multivariate outcomes and non‐monotone missing data and explicitly modelled the increment between times *t* − 1 and *t* as a function of the outcome at time *t* − 1. They also treated the special situation where some missing outcomes result from patients dying, and, as an alternative to estimating the compensator, introduced an imputation method (which we call ‘LI‐LS imputation’), which is particularly suitable when some patients die. Unlike DF&H's model, A&G's model does not explicitly allow for independent measurement error.

Many of the commonly used methods for handling missing data in longitudinal studies assume data are missing at random (MAR), that is, the probability an outcome is observed depends only on observed data (see Seaman *et al.* (2013) for a formal definition). Among these methods is the multivariate normal (MVN) model fitted using maximum likelihood (Schafer, 1997). The LI approach was originally conceived as a computationally simple way to handle data that are missing not at random (MNAR), that is, where the probability an outcome is observed can depend on the unobserved data in a particular way. It involves the ‘discrete‐time independent censoring (DTIC)’ assumption that the expected increment between times *t* − 1 and *t* given the underlying outcomes up to time *t* − 1 and the missingness pattern in the increments up to time *t* does not depend on that missingness pattern. LI–LS imputation additionally requires another, stronger DTIC assumption and an ‘independent return’ assumption, that is, an assumption about the probability that a patient with missing outcome at time *t* will be observed again at a later time point. The relation between DTIC and MAR has not been investigated in depth.

In this article, we have several aims. In Section 2, we show how DF&H's LI model can be used with non‐monotone missing data. We demonstrate that, contrary to DF&H's suggestion, this model cannot simultaneously allow for independent measurement error and dependence of the expected increment on the previous outcome. DF&H allowed for independent measurement error; A&G allowed for dependence on the previous outcome. In Section 3, we clarify the relation between the DTIC, MAR and independent return assumptions. In Section 4, we prove that under specific (MNAR) assumptions, which for a continuous outcome are not much stronger than those required by LI–LS imputation, fitting the MVN model ignoring the missingness mechanism gives consistent estimation. Section 5 describes how the LI model can allow for dependence of the expected increment on outcomes prior to the previous one. In Section 6, we demonstrate using simulation studies that the MVN approach can be more efficient than estimating the compensator and LI–LS imputation when data are non‐monotone missing. In Section 7, we identify that using imputation to treat the situation where some patients die requires an assumption not mentioned by A&G. Finally, Section 8 describes an application of the various methods to data from the British household panel survey (BHPS).

## 2 The linear increments method

### 2.1 The LI model

Let ***Y***_*it*_ denotes a vector of length *m* representing an underlying outcome for individual *i* at time *t* (*i* = 1,…,*N*; *t* = 1,…,*T*). The corresponding measured outcome is ***Y***_*it*_+***e***_*it*_, where ***e***_*it*_ is a measurement error. When the outcome is observed for individual *i* at time *t*, it is ***Y***_*it*_+***e***_*it*_ that is observed; ***Y***_*it*_ itself is never observed (except when ***e***_*it*_=0). Let ***X***_*i*_ be a vector of *p* fully‐observed baseline covariates for individual *i*. The change (or ‘increment’), Δ***Y***_*it*_=***Y***_*it*_−***Y***_*i*,*t* − 1_, in the underlying outcome between times *t* − 1 and *t* is assumed to be related to the underlying outcome at time *t* − 1 and the covariates by

(1) $$\Delta Y_{\mathrm{it}}=\alpha_{t}+{\left(\beta_{t}-I\right)}^{⊤}Y_{i,t-1}+\gamma_{t}^{⊤}X_{\;i}+\varepsilon_{\mathrm{it}}\;(t=2,\ldots ,T)$$

where ***β***_*t*_ and ***γ***_*t*_ are, respectively, *m* × *m* and *p* × *m* matrices of parameters, ***I*** is the identity matrix, and ***ε***_*it*_ is a random vector of length *m* satisfying $E(\varepsilon_{\mathrm{it}}|F_{i,t-1})=0$, with $F_{\mathrm{it}}=\{X_{\;i},Y_{\;\;i1},\ldots ,Y_{\;\;\mathrm{it}}\}$ denoting the history of individual *i*'s covariates and underlying outcomes up to time *t*. We assume ***ε***_*it*_ and ***e***_*it*_ have finite variance. An important special case of Equation (1) is where ***β***_*t*_=***I***: then the expectation of Δ***Y***_*it*_ does not depend on ***Y***_*i*,*t* − 1_. Equation (1) can be written equivalently as

(2) $$Y_{\;\;\mathrm{it}}=\alpha_{t}+\beta_{t}^{⊤}Y_{\;\;i,t-1}+\gamma_{t}^{⊤}X_{\;i}+\varepsilon_{\mathrm{it}}\;(t=2,\ldots ,T).$$

Like DF&H, we assume that the measurement error process {***e***_*it*_:*t* = 1,…,*T*} is independent of all other processes (i.e. ***e***_*i*1_,…,***e***_*iT*_ are independent of ***X***_*i*_,***Y***_*i*1_,***ε***_*i*2_,…,***ε***_*iT*_), that ***e***_*it*_ is independent of ***e***_*is*_ for all *t* ≠ *s*, and that *E*(***e***_*it*_) = **0**. The underlying outcome processes, measurement error processes and missingness processes (described in Section 2.2) of different individuals are assumed independent (given ***X***_*i*_,***Y***_*i*1_).

Some elements of ***β***_*t*_ and/or ***γ***_*t*_ may be constrained. For example, if baseline outcome ***Y***_*i*1_+***e***_*i*1_ is included in ***X***_*i*_, then ***β***_2_ can be constrained to equal ***I*** for identifiability. In much of this article, we shall consider two special cases of Equation (2). The first, which we call the case of ‘no independent measurement error’ (or just ‘no measurement error’) is where ***e***_*it*_=**0**∀*i*,*t*. Strictly speaking, we mean by ‘no measurement error’ that there are no mutually independent measurement errors that are independent of ***X***_*i*_, ***Y***_*i*1_, ***ε***_*i*2_, …, ***ε***_*iT*_ and whose variance is unknown (see Section 9 for case of known variance). The second case is where ***β***_*t*_=***I***∀*t*.

### 2.2 Missing data

Let *R*_0,*it*_=1 if ***Y***_*it*_+***e***_*it*_ is observed and *R*_0,*it*_=0 if ***Y***_*it*_+***e***_*it*_ is missing. Let *R*_*it*_=1 if *R*_0,*it*_=*R*_0,*it* − 1_=1 and *R*_*it*_=0 otherwise. Hence, *R*_*it*_ indicates whether the measured increment (***Y***_*it*_+***e***_*it*_) − (***Y***_*i*,*t* − 1_+***e***_*i*,*t* − 1_) is observed, and *R*_*it*_=1 implies *R*_0,*it*_=1. Let $R_{0,\mathrm{it}}={\left(R_{0,i1},\ldots ,R_{0,\mathrm{it}}\right)}^{⊤}$ and $R_{\mathrm{it}}={\left(R_{i1},\ldots ,R_{\mathrm{it}}\right)}^{⊤}$ denote missingness histories up to and including time *t*. We assume *R*_0,*i*1_=1∀*i*. The assumption that the measurement error process is independent of all other processes means that *R*_0,*i*1_,…,*R*_0,*iT*_ are independent of ***e***_*i*1_,…,***e***_*iT*_. We say that the ‘data are monotone missing’ if *P*(*R*_0,*it* + 1_=1∣*R*_0,*it*_=0) = 0 for all *i* (i.e. we mean that the data‐generating mechanism always generates monotone missing data). Let ***G***_*it*_ denote the vector consisting of ***X***_*i*_ and those elements of ***Y***_*i*1_,…,***Y***_*it*_ whose corresponding elements of *R*_0,*it*_ equal one. So, ***G***_*it*_ consists of the baseline covariates and the underlying outcomes at the times at which the measured (with error) outcome is observed. For example, if $R_{0,i4}={\left(1,1,0,1\right)}^{⊤}$, then $G_{i4}={\left(Y_{\;\;i1}^{⊤},Y_{i2}^{⊤},Y_{i4}^{⊤},X_{\;i}^{⊤}\right)}^{⊤}$. Henceforth, we omit the *i* index, unless doing so causes ambiguity.

DF&H define DTIC as $E(\Delta Y_{\;\;t}|F_{t-1},e_{1},\ldots ,e_{t-1},R_{t})=E(\Delta Y_{\;\;t}|F_{t-1},,e_{1},\ldots ,e_{t-1})$ for all *t*. Because it is assumed that the measurement error process is independent of all other processes, this definition reduces to

(3) $$E(\Delta Y_{\;\;t}|F_{t-1},R_{t})=E(\Delta Y_{\;\;t}|F_{t-1})\;\;\forall t.$$

Note that Equation (3) can be equivalently written as $E(Y_{\;\;t}|F_{t-1},R_{t})=E(Y_{\;\;t}|F_{t-1})\;\forall t$. We discuss the relation between DTIC and MAR in Section 3. A&G give two versions of DTIC: Equation (3) and

(4) $$E(Y_{\;\;t}|F_{t-1},R_{0,t})=E(Y_{\;\;t}|F_{t-1})\;\;\forall t.$$

For convenience, we call (3) and ((4)) ‘weak DTIC’ and ‘strong DTIC’, respectively. A&G point out that strong DTIC implies weak DTIC, and that when data are monotone missing, weak DTIC implies strong DTIC (i.e. they are equivalent).

### 2.3 Parameter estimation

Consider the model

(5) $$E(Y_{\;\;t}+e_{t})=\alpha_{t}^{\text{ls}}+{\left(\beta_{t}^{\text{ls}}\right)}^{⊤}(Y_{\;\;t-1}+e_{t-1})+{\left(\gamma_{t}^{\text{ls}}\right)}^{⊤}X.$$

In general, $\alpha_{t}^{\text{ls}}$, $\beta_{t}^{\text{ls}}$ and $\gamma_{t}^{\text{ls}}$ differ from the parameters of interest ***α***_*t*_, ***β***_*t*_ and ***γ***_*t*_ in Equation (2). The former relates *measured* outcomes, the latter *underlying* outcomes. Let ${\hat{\alpha }}_{t}^{\text{ls}}$, ${\hat{\beta }}_{t}^{\text{ls}}$ and ${\hat{\gamma }}_{t}^{\text{ls}}$ denote the least‐squares estimators of $\alpha_{t}^{\text{ls}}$, $\beta_{t}^{\text{ls}}$ and $\gamma_{t}^{\text{ls}}$ obtained by fitting model ((5)) to the set of patients with *R*_*t*_=1. DF&H offered a proof (Section 4.2) that ${\hat{\alpha }}_{t}^{\text{ls}}$, ${\hat{\beta }}_{t}^{\text{ls}}$ and ${\hat{\gamma }}_{t}^{\text{ls}}$ are unbiased consistent estimators of ***α***_*t*_, ***β***_*t*_ and ***γ***_*t*_ in Equation (2) when weak DTIC holds, data are monotone missing and *m* = 1. However, this proof implicitly assumes that either (i) *β*_*t*_=1 and ${\hat{\beta }}_{t}^{\text{ls}}$ is fixed at 1 before calculating the least‐squares estimator of $\left(\alpha_{t}^{\text{ls}},\gamma_{t}^{\text{ls}}\right)$, or (ii) *E*{(*Y*_*t* − 1_+*e*_*t* − 1_)*e*_*t* − 1_}=0. Because (ii) is not true unless Var(*e*_*t* − 1_) = 0, this proof is not valid unless either (i) the expectation of the increment Δ*Y*_*it*_ does not depend on *Y*_*t* − 1_ (i.e. *β*_*t*_=1) and ${\hat{\beta }}_{t}^{\text{ls}}$ is fixed at the true value of *β*_*t*_ or (ii) there is no measurement error. This lack of validity is demonstrated by Example 1 later. A&G also proved that ${\hat{\alpha }}_{t}^{\text{ls}}$, ${\hat{\beta }}_{t}^{\text{ls}}$ and ${\hat{\gamma }}_{t}^{\text{ls}}$ are unbiased estimators of ***α***_*t*_, ***β***_*t*_ and ***γ***_*t*_ when weak DTIC holds and there is no measurement error. Their proof allows *m* > 1 and data to be non‐monotone missing.

The following theorem and corollary show that if weak DTIC holds and ${\hat{\beta }}_{t}^{\text{ls}}$ is fixed at the true value of ***β***_*t*_, then $\left({\hat{\alpha }}_{t}^{\text{ls}},{\hat{\gamma }}_{t}^{\text{ls}}\right)$ is an unbiased consistent estimator of (***α***_*t*_,***γ***_*t*_) even if missingness is non‐monotone and/or there is measurement error. As explained later, this theorem is of most practical interest when the true value of ***β***_*t*_ is ***I***. The theorem is followed by an example which illustrates that the least‐squares estimators may be neither unbiased nor consistent estimators of the parameters of interest when there is measurement error unless ${\hat{\beta }}_{t}^{\text{ls}}$ is fixed at the true value of ***β***_*t*_.

Theorem 1 If the increments model of Equation (2) and the weak DTIC assumption of Equation (3) hold and ${\hat{\beta }}_{t}^{\text{ls}}$ is fixed at the true value of ***β***_*t*_, then $E\{({\hat{\alpha }}_{t}^{\text{ls}},{\hat{\gamma }}_{t}^{\text{ls}})\}=(\alpha_{t},\gamma_{t})$ and $\left({\hat{\alpha }}_{t}^{\text{ls}},{\hat{\gamma }}_{t}^{\text{ls}})\to (\alpha_{t},\gamma_{t}\right)$ as *N*→*∞*.

Proofs of theorems are given in Appendix S1.

Example 1 *m* = 1, *T* = 2, *Y*_1_∼Normal(0,1), *e*_1_∼Normal(0,1), *e*_2_=0 and *Y*_2_=*Y*_1_. Note that this is a special case of Equation (2) in which *α*_2_=*ε*_2_=0, *β*_2_=1, and there is no covariate ***X***. It can be shown that $\alpha_{2}^{\text{ls}}=0$ and $\beta_{2}^{\text{ls}}=0.5$. Thus, even if there are no missing data, ${\hat{\alpha }}_{2}^{\text{ls}}$ and ${\hat{\beta }}_{2}^{\text{ls}}$ are not unbiased or consistent estimators of *α*_2_ and *β*_2_. Moreover, when there is missing data, they may not even be unbiased or consistent estimators of $\alpha_{2}^{\text{ls}}$ and $\beta_{2}^{\text{ls}}$. For example, suppose that *R*_02_=1 if and only if *Y*_1_≥0. It can be shown that if *Y*_2_+*e*_2_=*Y*_2_ is regressed on *Y*_1_+*e*_1_ using only those individuals with *R*_02_=1, then ${\hat{\alpha }}_{2}^{\text{ls}}$ and ${\hat{\beta }}_{2}^{\text{ls}}$ converge to 0.585 and 0.267, respectively, as *N*→*∞*. Hence, ${\hat{\beta }}_{2}^{\text{ls}}$ is a consistent estimator neither of *β*_2_=1 nor of $\beta_{2}^{\text{ls}}=0.5$. Note that if ${\hat{\beta }}_{2}^{\text{ls}}$ is fixed at *β*_2_=1, then Theorem 1 implies that ${\hat{\alpha }}_{2}^{\text{ls}}$ converges to *α*_2_=0.

In conclusion, the least‐squares estimators are unbiased for the parameters of the LI model if weak DTIC holds and either (i) ${\hat{\beta }}_{t}^{\text{ls}}$ is fixed at the true value of ***β***_*t*_, or (ii) there is no measurement error. Otherwise, they may not be. Henceforth, the only value at which we consider fixing ${\hat{\beta }}_{t}^{\text{ls}}$ is ***I***. This is the case of most practical interest: one could fix ${\hat{\beta }}_{t}^{\text{ls}}=I$ if one believed that expected increment $E(\Delta Y_{\;\;t}|F_{t-1})$ did not depend on ***Y***_*t* − 1_ (i.e. ***β***_*t*_=***I***). Otherwise, one could treat ***β***_*t*_ as unknown and estimate it. It seems unlikely one would wish to fix ***β***_*t*_ at a value other than ***I***.

### 2.4 Estimating the compensator

From Equation (2), $E(Y_{\;\;t}|X,Y_{\;\;1})=\alpha_{t}+\beta_{t}^{⊤}E(Y_{\;\;t-1}|X,Y_{\;\;1})+\gamma_{t}^{⊤}X$, and so

(6) $$E(Y_{\;\;t}|X,Y_{\;\;1})=\alpha_{t}+\gamma_{t}^{⊤}X+\left\{\overset{t-1}{\underset{j=2}{\sum }}\left(\overset{t}{\underset{k=j+1}{\prod }}\beta_{k}^{⊤}\right)(\alpha_{j}+\gamma_{j}^{⊤}X)\right\}+\overset{t}{\underset{j=2}{\prod }}\beta_{j}^{⊤}Y_{\;\;1}$$

where $\overset{b}{\underset{j=a}{\prod }}\beta_{j}^{⊤}$ means $\beta_{b}^{⊤}\beta_{b-1}^{⊤}\ldots \beta_{a}^{⊤}$. Thus, *E*(***Y***_*it*_∣***X***_*i*_,***Y***_*i*1_) can be estimated for each individual *i* in the dataset by replacing ***α***_*t*_, ***β***_*t*_ and ***γ***_*t*_ in Equation (6) with ${\hat{\alpha }}_{t}^{\text{ls}}$, ${\hat{\beta }}_{t}^{\text{ls}}$ and ${\hat{\gamma }}_{t}^{\text{ls}}$. Denote this estimate as $Y_{\;\;\mathrm{it}}^{\text{cpr}}$. This is the estimating the compensator method. The overall mean *E*(***Y***_*t*_) can then be estimated as $N^{-1}\overset{N}{\underset{i=1}{\sum }}Y_{\;\;\mathrm{it}}^{\text{cpr}}$. If $\left({\hat{\alpha }}_{t}^{\text{ls}},{\hat{\beta }}_{t}^{\text{ls}},{\hat{\gamma }}_{t}^{\text{ls}}\right)$ is an unbiased consistent estimator of (***α***_*t*_,***β***_*t*_,***γ***_*t*_), then this estimate of *E*(***Y***_*t*_) will also be unbiased and consistent.

Returning to Example 1, if ${\hat{\alpha }}_{2}^{\text{ls}}=0.585$ and ${\hat{\beta }}_{2}^{\text{ls}}=0.267$ are used in Equation (6), then *E*(*Y*_2_) is calculated to equal ${\hat{\alpha }}_{2}^{\text{ls}}=0.585$, whereas its true value is zero.

### 2.5 LI–LS imputation

A&G propose an alternative to estimating the compensator when there is no measurement error. We call this method ‘LI‐LS imputation’, because it is based on the LI model of Equation (2) and the least‐squares (LS) estimators ${\hat{\alpha }}_{t}^{\text{ls}},{\hat{\beta }}_{t}^{\text{ls}},{\hat{\gamma }}_{t}^{\text{ls}}$ of ***α***_*t*_, ***β***_*t*_ and ***γ***_*t*_. (Later, we shall introduce other LI imputation methods that differ from LI‐LS imputation only in that they use alternative estimators of ***α***_*t*_, ***β***_*t*_ and ***γ***_*t*_.) LI–LS imputation uses an actual outcome when it is observed and imputes a missing outcome as the most recently observed outcome updated by the expected increments. That is, letting $Y_{\;\;t}^{\text{est}}$ denotes the value of ***Y***_*t*_ in the imputed dataset, we have $Y_{\;\;t}^{\text{est}}=Y_{\;\;t}$ if *R*_0*t*_=1 and $Y_{\;\;t}^{\text{est}}={\hat{\alpha }}_{t}^{\text{ls}}+{\left({\hat{\beta }}_{t}^{\text{ls}}\right)}^{⊤}Y_{\;\;t-1}^{\text{est}}+{\left({\hat{\gamma }}_{t}^{\text{ls}}\right)}^{⊤}X$ if *R*_0*t*_=0.

A&G show that if Equation (2) holds, there is no measurement error, strong DTIC holds and

(7) $$E\{R_{0,t}(Y_{t-1}^{\text{est}}-Y_{\;\;t-1})|X,Y_{\;\;1}\}=0$$

then $E(Y_{\;\;t}^{\text{est}}|Y_{\;\;1},X)=E(Y_{\;\;t}|Y_{\;\;1},X)$. It follows that $N^{-1}\overset{N}{\underset{i=1}{\sum }}Y_{\;\;\mathrm{it}}^{\text{est}}$ is an unbiased estimator of *E*(***Y***_*t*_) and that if a linear regression model for ***Y*** with any or all of ***X*** and *t* as covariates is fitted to the imputed dataset $\left\{Y_{\;\;\mathrm{it}}^{\text{est}}:i=1,\ldots ,N;\;t=1,\ldots ,T\right\}$ using least squares, then the parameter estimators of this model are consistent.

The following theorem shows that LI–LS imputation can also be used when there is measurement error, provided that ***β***_*t*_=***I*** and ${\hat{\beta }}_{t}^{\text{ls}}$ is fixed at ***I***.

Theorem 2 Suppose that the increments model of Equation (2), the strong DTIC assumption of Equation ((4)) and Equation (7) hold, that ***β***_*t*_=***I***, and that ${\hat{\beta }}_{t}^{\text{ls}}$ is fixed at ***I***. Then, $E(Y_{\;\;t}^{\text{est}}|Y_{\;\;1},X)=E(Y_{\;\;t}|Y_{\;\;1},X)$.

If the conditions of Theorem 2 are satisfied, then (i) $N^{-1}\overset{N}{\underset{i=1}{\sum }}Y_{\;\;\mathrm{it}}^{\text{est}}$ is an unbiased estimator of *E*(***Y***_*t*_) and (ii) if a linear regression model for ***Y*** with any or all of ***X*** and *t* as covariates is fitted to the imputed dataset using least squares, the parameter estimators are asymptotically unbiased.

Returning to Example 1, it can be shown that $N^{-1}\overset{N}{\underset{i=1}{\sum }}Y_{i2}^{\text{est}}$ tends to 0.585 as *N*→*∞*, and so is an inconsistent estimator of *E*(***Y***_2_) = 0.

A&G say that Equation (7) will hold if

(8) $$\begin{array}{cc} P(R_{0,t} & \;=\;1\;|\;R_{0,k-1},\;R_{0,k}\;=\;1,R_{0,k+1}\;\;=\;R_{0,k+2}\;=\;\ldots \;=\;R_{0,t-1}\;=\;0,\;X,\;Y_{\;\;k},Y_{\;\;k\;+\;1},\ldots ,\;Y_{\;\;t})\\ \;=\;P(R_{0,t}\;=\;1\;|\;R_{0,k-1},R_{0,k}\;=\;1,R_{0,k+1}=R_{0,k+2}\;=\ldots =R_{0,t-1}=0,X,Y_{\;\;k}) \end{array}$$

for all *k*≤*t* − 2, and imply that it is unlikely to hold otherwise. Equation (8) can be interpreted as meaning that the conditional probability of return at time *t* given dropout immediately after time *k*, no return between times *k* and *t*, the baseline covariates ***X***, the most recently observed outcome ***Y***_*k*_ and subsequent outcomes does not depend on these subsequent outcomes (or, more informally, as ‘return after a dropout is independent of outcomes occurring since that dropout’). We shall call Equation (8) the ‘independent return’ assumption.

### 2.6 Estimating the compensator versus LI–LS imputation

A&G do not discuss the relative advantages and disadvantages of the two methods in detail but do say that LI–LS imputation can be used to give mortal‐cohort inference (as well as immortal‐cohort inference), whereas estimating the compensator gives immortal‐cohort inference only. We postpone consideration of mortal‐cohort inference until Section 7. The advantage of estimating the compensator is that it relies on fewer assumptions than LI–LS imputation. Notably, it does not make assumptions about the probability of return after dropout. The disadvantages are that it may be less efficient and less robust to violation of those assumptions than LI–LS imputation. LI–LS imputation may be more efficient because, unlike estimating the compensator, it uses ***Y***_*t*_+***e***_*t*_ values with *R*_0,*t*_=1 but *R*_0,*t* − 1_=0 (so that *R*_*t*_=0). It may be more robust to violation of the weak DTIC assumption because it relies on DTIC only to impute missing values. For example, if ***Y***_3_+***e***_3_ is fully observed and ***Y***_2_+***e***_2_ is missing whenever ***Y***_2_<0, then the estimate of *E*(***Y***_3_) from LI–LS imputation will be unbiased, whereas that from estimating the compensator will be biased. Note that when data are monotone missing, the two estimators $N^{-1}\overset{N}{\underset{i=1}{\sum }}Y_{\;\;\mathrm{it}}^{\text{cpr}}$ and $N^{-1}\overset{N}{\underset{i=1}{\sum }}Y_{\;\;\mathrm{it}}^{\text{est}}$ of *E*(***Y***_*t*_) are equal (Aalen & Gunnes, 2010).

## 3 Relation between missingness assumptions

In this section, we consider the relation between DTIC and MAR and then the implications for the dropout and return processes of assuming DTIC.

Weak and strong DTIC are assumptions only about expectations. MAR, on the other hand, is an assumption about whole distributions. A slightly stronger assumption than strong DTIC, which is about whole distributions, is

(9) $$f(Y_{\;\;t}|F_{t-1},R_{0,t})=f(Y_{\;\;t}|F_{t-1})\;\;\forall t.$$

We call Equation (9) ‘dDTIC’ (‘DTIC in distribution’). In practice, it seems likely that in most cases when strong DTIC holds, dDTIC will also hold. dDTIC can be written equivalently as

(10) $$P(R_{0,t}=1|R_{0,t-1},F_{T})=P(R_{0,t}=1|R_{0,t-1},F_{t-1})\;\;\;\;\forall t.$$

(see Appendix S2). Thus, the hazards of dropout and return are not allowed to depend on future underlying outcomes.

Missing at random means that the probability the outcome is missing at time *t* can depend on covariates ***X*** and observed outcomes, including future observed outcomes, but not on missing outcomes (i.e. those at times *t* where *R*_0*t*_=0). When there is measurement error, missingness depends on the outcomes observed with measurement error, not on the underlying outcomes. Conversely, the dDTIC assumption, combined with the assumed independence of the measurement error and missingness processes, allows the probability of missingness to depend on *all* past underlying outcomes (including those at times *t* where *R*_0*t*_=0) but not on future underlying outcomes or the outcomes measured with error. When data are monotone missing and there is no measurement error, dDTIC is equivalent to MAR (and also to ‘sequential MAR’ — Hogan *et al*. (2004); Diggle *et al*. (2007)).

A simple example illustrates that estimating the compensator can give inconsistent estimation when there is measurement error and dropout depends on it. Suppose that *m* = 1 and *T* = 2, that *Y*_1_=*Y*_2_=0, that *e*_1_ and *e*_2_ are independently distributed Normal(0,1), that there are no baseline covariates ***X***, that *R*_02_=1 if and only if *Y*_1_+*e*_1_>0, and that ${\hat{\beta }}_{2}^{\text{ls}}$ is constrained to equal 1. Whereas *α*_2_=0, the least‐squares estimator ${\hat{\alpha }}_{2}^{\text{ls}}$ has negative expectation. Note that these data are MAR.

The following example illustrates that dDTIC and independent return can hold without MAR holding. In this example, the probability that an individual drops out at time *t* = 4 depends on the outcome at *t* = 2, which may be missing.

Example 2 *m* = 1 and *T* = 4, *α*_*t*_=0 and *β*_*t*_=1 for all *t*, there is no covariate ***X*** or measurement error, *P*(*R*_02_=1∣*Y*_1_,*Y*_2_,*Y*_3_,*Y*_4_) = 0.5, *R*_03_=1 always, and *R*_04_=1 if and only if *Y*_2_>0.

If, in addition to Equation (10), it is assumed that

(11) $$\begin{array}{cc} P(R_{0,t} & =1|R_{0,k-1},R_{0,k}=1,R_{0,k+1}=\ldots =R_{0,t-1}=0,F_{t-1})\\ =P(R_{0,t}=1|R_{0,k-1},R_{0,k}=1,R_{0,k+1}=\ldots =R_{0,t-1}=0,F_{k})\;\;\;\;\forall k \end{array}$$

then the hazard of return after dropout cannot depend on any of the underlying outcomes after the most recent dropout. The following theorem shows that Equation (11) is sufficient but not necessary for independent return to hold.

Theorem 3 Let ***B***_*k*_ and ***C***_*k*_ be subvectors of ${\left(X^{⊤},Y_{\;\;1}^{⊤},\ldots ,Y_{\;\;k}^{⊤}\right)}^{⊤}$ such that both contain ${\left(X^{⊤},Y_{\;\;k}^{⊤}\right)}^{⊤}$ and ***C***_*k*_ is a subvector of ***B***_*k*_. If the increments model of Equation (2) and the dDTIC assumption of Equation (9) hold, then

$$\begin{array}{cc} P(R_{0,t} & =1|R_{0,k-1},R_{0,k}=1,R_{0,k+1}=\ldots =R_{0,t-1}=0,B_{k},Y_{\;\;k+1},\ldots ,Y_{\;\;T})\\ =P(R_{0,t}=1|R_{0,k-1},R_{0,k}=1,R_{0,k+1}=\ldots =R_{0,t-1}=0,B_{k}), \end{array}$$

implies

$$\begin{array}{cc} P(R_{0,t} & =1|R_{0,k-1},R_{0,k}=1,R_{0,k+1}=\ldots =R_{0,t-1}=0,C_{k},Y_{\;\;k+1},\ldots ,Y_{\;\;T})\\ =P(R_{0,t}=1|R_{0,k-1},R_{0,k}=1,R_{0,k+1}=\ldots =R_{0,t-1}=0,C_{k}). \end{array}$$

The converse is not true in general.

Theorem 3 also implies that the following assumption, which will be important in Section 4.1, is stronger than independent return but weaker than Equation (11):

(12) $$\begin{array}{cc} P(R_{0,t} & =1|R_{0,k-1},R_{0,k}=1,R_{0,k+1}=R_{0,k+2}=\ldots =R_{0,t-1}\\ =0,G_{k},Y_{\;\;k+1},Y_{\;\;k+2},\ldots ,Y_{\;\;t})\\ =P(R_{0,t}=1|R_{0,k-1},R_{0,k}\;=1,R_{0,k+1}=R_{0,k+2}=\ldots =R_{0,t-1}=0,G_{k}) \end{array}$$

for all *k*≤*t* − 2. We call Equation (12) the ‘strong independent return’ assumption. In the proof of Theorem 3, we give an example where independent return holds but strong independent return does not. However, in most real‐world situations, we believe it is unlikely that independent return would hold without strong independent return also holding. Note that the word ‘strong’ in ‘strong independent return’ is meant in a different sense from that in ‘strong DTIC’.

## 4 Use of MVN model assuming ignorability

A popular method for handling missing repeated outcome data is to assume that outcomes and covariates are normally distributed and calculate the maximum likelihood estimates of the mean and variance of this normal distribution ignoring the missingness mechanism and imposing no structure on the mean and variance (Schafer, 1997). We call this the ‘unstructured MVN’ method. These maximum likelihood estimates may be of interest in themselves, or they can be used to impute missing values. This approach yields consistent estimates when data are MAR and the MVN model is correctly specified (Seaman *et al.*, 2013). (Indeed, under these conditions, the missingness mechanism is ‘ignorable’ in the following sense. The unstructured MVN method yields the same maximum likelihood estimates and imputed values as would be obtained if the MVN model were fitted jointly with any missingness model—that is, model for the missingness pattern given the outcomes and covariates—that assumes MAR and has parameters distinct from those of the MVN model (Seaman et al., 2013)). As we have noted, the assumptions used in estimating the compensator (weak DTIC) and LI–LS imputation (strong DTIC and Equation (7)) do not imply the data are MAR or normally distributed. Nevertheless, we now show (Section 4.1) that when there is no measurement error and slightly stronger assumptions than those of LI–LS imputation are satisfied, the unstructured MVN method yields consistent estimates *even when MAR does not hold and the data are not normally distributed*. We then show (Section 4.2) that a more efficient estimator can be obtained by constraining the variance matrix of the MVN distribution. We call this the ‘autoregressive MVN’ method. Finally, we show (Section 4.3) that if a further constraint is applied to the variance matrix, the resulting method provides consistent estimates even when there is measurement error, provided that ***β***_*t*_=***I*** for all *t*. We call this the ‘random‐walk MVN’ method. These MVN methods are typically more efficient than estimating the compensator and LI–LS imputation.

### 4.1 Unstructured MVN and no measurement error

We assume throughout Sections 4.1 and 4.2 that there is no measurement error, that is, ***e***_*t*_=0. Let $\mu =E\{{\left(Y_{\;\;1}^{⊤},\ldots ,Y_{T}^{⊤},X^{⊤}\right)}^{⊤}\}$ and $\Sigma =\mathrm{Var}\{{\left(Y_{\;\;1}^{⊤},\ldots ,Y_{\;\;T}^{⊤},X^{⊤}\right)}^{⊤}\}$. Let ***μ***_*t*_ denotes the subvector of ***μ*** corresponding to ***Y***_*t*_ (*t* = 1,…,*T* + 1), where ***Y***_*T* + 1_ means ***X***. Similarly, let **Σ**_*s*,*t*_ denotes the submatrix of **Σ** corresponding to (***Y***_*s*_,***Y***_*t*_) (1≤*s*,*t*≤*T* + 1). So, for example, ***μ***_3_=*E*(***Y***_3_), ***μ***_*T* + 1_=*E*(***X***), **Σ**_3,*T* + 1_=Cov(***Y***_3_,***X***) and **Σ**_*T* + 1,*T* + 1_=Var(***X***).

Let $\hat{\mu }$ and $\hat{\Sigma }$ denote the maximum likelihood estimates obtained by fitting the model ${\left(Y_{\;\;1}^{⊤},\ldots ,Y_{\;\;T}^{⊤},X^{⊤}\right)}^{⊤}∼\text{Normal}(\mu ,\Sigma )$ with unstructured ***μ*** and **Σ** to the observed data and ignoring the missingness mechanism. That is, $\hat{\mu }$ and $\hat{\Sigma }$ are the maximum likelihood estimates from the model $G_{\;\;\mathrm{iT}}∼\text{Normal}(\mu_{R_{0,\mathrm{iT}}},\;\Sigma_{R_{0,\mathrm{iT}},R_{0,\mathrm{iT}}})(i=1,\ldots ,N)$, where $\mu_{R_{0,\mathrm{iT}}}$ and $\Sigma_{R_{0,\mathrm{iT}},R_{0,\mathrm{iT}}}$ denote the subvector of ***μ*** and submatrix of **Σ** corresponding to ***G***_*iT*_. This model can be fitted using an EM algorithm (e.g. using the norm package in R) (Schafer, 1997).

Theorem 4 If the increments model of Equation (2), the dDTIC assumption of Equation (9) and the strong independent return assumption of Equation (12) hold and $\mathrm{Var}(\varepsilon_{t}|F_{t-1})=\mathrm{Var}(\varepsilon_{t})$ for all *t*, then $\left(\hat{\mu },\hat{\Sigma }\right)$ is a consistent estimator of (***μ***,**Σ**).

Theorem 4 may be surprising, because there is no assumption the data are MAR (e.g. Example 2 satisfies the conditions of Theorem 4, but the data are not MAR) nor that they are normally distributed. Note that Theorem 4 is not saying the missingness process is ‘ignorable’. In Example 2, missingness is not ignorable, because information about a missing value of *Y*_2_ is gained by knowing whether *Y*_4_ is observed. The intuition behind Theorem 4 is that although dDTIC and strong independent return allow dropout and return to depend on earlier unobserved outcomes, this dependence does not matter, because the autoregressive structure of the data means that the later outcomes are conditionally independent of these earlier outcomes given outcomes that are observed. Consider Example 2. Although whether or not *Y*_4_ is observed depends on *Y*_2_, which may be missing, *Y*_4_ is independent of *Y*_2_ given *Y*_3_, which is observed. This results in observed outcomes being in an important sense representative of all the outcomes, as formalized in the following theorem.

Theorem 5 If the increments model of Equation (2), the dDTIC assumption of Equation (9) and the strong independent return assumption of Equation (12) hold, then for *k* < *t*,

(13) $$\begin{array}{c} \text{a)}f(Y_{\;\;t}|R_{0,t-2},R_{0,t-1}=R_{0,t}=1,G_{t-1})=f(Y_{\;\;t}|X,Y_{\;\;t-1})\\ \text{b)}f(Y_{\;\;t}|R_{0,k-1},R_{0,k}=1,R_{0,k+1}=\ldots =R_{0,t-1}=0,R_{0,t}=1,G_{k}) \end{array}$$

(14) $$\;\;=f(Y_{\;\;t}|X,Y_{\;\;k})$$

If Equation (8) holds instead of Equation (12), then Equations ((13)) and ((14)) still hold but with ***G***_*t* − 1_ and ***G***_*k*_ replaced by, respectively, (***X***,***Y***_*t* − 1_) and (***X***,***Y***_*k*_).

Equation ((13)) says that the conditional distribution of the outcome at time *t* given the previous observed outcomes in individuals who are observed at times *t* and *t* − 1 is the same as the distribution in the whole sample. Equation ((14)) says that the same is true of individuals returning after dropout. Because the joint distribution of the observed part of ${\left(Y_{\;\;1}^{⊤},\ldots ,Y_{T}^{⊤},X^{⊤}\right)}^{⊤}$ can be factorized as the product of these conditional distributions, the parameters (***μ***,**Σ**) of the joint distribution can be consistently estimated (see proof of Theorem 4 for more details).

Having obtained estimates $\hat{\mu }$ and $\hat{\Sigma }$, the corresponding estimates of ***α***_*t*_, ***β***_*t*_ and ***γ***_*t*_ can be calculated as follows, and these used for LI imputation in place of the least‐squares estimates ${\hat{\alpha }}_{t}^{\text{ls}}$, ${\hat{\beta }}_{t}^{\text{ls}}$, and ${\hat{\gamma }}_{t}^{\text{ls}}$. We call this method ‘LI‐uMVN imputation’. Let

(15) $$\begin{array}{cc} {\hat{\beta }}_{t}= & {\left({\hat{\Sigma }}_{t-1,t-1}-{\hat{\Sigma }}_{t-1,T+1}{\hat{\Sigma }}_{T+1,T+1}^{-1}{\hat{\Sigma }}_{T+1,t-1}\right)}^{-1}\\ \left({\hat{\Sigma }}_{t-1,t}-{\hat{\Sigma }}_{t-1,T+1}{\hat{\Sigma }}_{T+1,T+1}^{-1}{\hat{\Sigma }}_{T+1,t}\right) \end{array}$$

(16) $${\hat{\gamma }}_{t}={\hat{\Sigma }}_{T+1,T+1}^{-1}{\hat{\Sigma }}_{T+1,t}-{\hat{\Sigma }}_{T+1,T+1}^{-1}{\hat{\Sigma }}_{T+1,t-1}{\hat{\beta }}_{t}$$

(17) $${\hat{\alpha }}_{t}={\hat{\mu }}_{t}-{\hat{\beta }}_{t}{\hat{\mu }}_{t-1}-\hat{\gamma }{\hat{\mu }}_{T+1}$$

where ${\hat{\mu }}_{t}$ denotes the *t*th element of $\hat{\mu }$ and ${\hat{\Sigma }}_{s,t}$ denotes the (*s*,*t*)th element of $\hat{\Sigma }(s,t=1,\ldots ,T+1$, with the (*T* + 1)th element corresponding to ***X***).

Theorem 6 If $\hat{\mu }$ and $\hat{\Sigma }$ are consistent estimators of ***μ*** and **Σ**, then ${\hat{\alpha }}_{t},{\hat{\beta }}_{t}$ and ${\hat{\gamma }}_{t}$ are consistent estimators of ***α***_*t*_,***β***_*t*_ and ***γ***_*t*_.

LI–uMVN imputation might be expected to be more efficient than estimating the compensator and LI–LS imputation, because the latter methods estimate ***α***_*t*_, ***β***_*t*_ and ***γ***_*t*_ using only those observed outcomes ***Y***_*t*_ with *R*_0,*t* − 1_=1, whereas the MVN method uses all observed outcomes.

If the motivation for using LI imputation is to enable a linear regression model for ***Y*** with any or all of ***X*** and *t* as covariates to be fitted to the imputed dataset, then an even more efficient alternative is available. It can be seen that the maximum likelihood estimates of the parameters in this linear regression are functions of $\hat{\mu }$ and $\hat{\Sigma }$. A computationally convenient way to calculate these functions is to impute each missing ***Y***_*t*_ value as its conditional expectation given ***G***_*iT*_ (given by Equation ((18)) with ***μ*** and **Σ** replaced by $\hat{\mu }$ and $\hat{\Sigma }$) and then fit the linear regression to the imputed data. We call this method ‘uMVN imputation’. More details and justification are given in Appendix S6. Unlike in LI imputation, where a missing value of ***Y***_*t*_ is imputed using only the individual's observed past (***G***_*t* − 1_), in uMVN imputation, missing values are imputed using his or her observed past and future (***G***_*T*_), and hence uMVN imputation may be more efficient when the data are non‐monotone missing. The conditional expectation of ***Y***_*t*_ given ***G***_*iT*_ is

(18) $$\mu_{t}+\Sigma_{t,R_{0,\mathrm{iT}}}\Sigma_{R_{0,\mathrm{iT}},R_{0,\mathrm{iT}}}^{-1}\left(G_{\;\;\mathrm{iT}}-\mu_{R_{0,\mathrm{iT}}}\right)$$

where $\Sigma_{t,R_{0,\mathrm{iT}}}$ is the submatrix of **Σ** composed of the *m* rows corresponding to ***Y***_*t*_ and the columns corresponding to ***G***_*iT*_.

So far, we have assumed there are baseline covariates in the model and that ***Y***_1_ is not treated as one of these covariates. If there are no baseline covariates, then ***γ***_*t*_, ***X***, **Σ**_*T* + 1,*t*_, **Σ**_*t*,*T* + 1_ and **Σ**_*T* + 1,*T* + 1_ should be omitted from all expressions in this article. If, on the other hand, ***Y***_1_ is included in ***X***, then ***β***_2_ should be set equal to zero to ensure parameter identifiability.

Theorem 4 (and hence LI‐uMVN imputation and uMVN imputation) requires the assumption that the variance of ***ε***_*t*_ does not depend on the history $F_{t-1}$. This is not required by the estimating the compensator and LI–LS imputation methods. When ***Y***_*t*_ is a continuous variable, one might be content to make this assumption. However, the LI approach can also be used for categorical outcomes. For example, A&G consider a Markov chain with *q* states. They define ***Y***_*t*_ to be a vector of *q* − 1 indicator variables for the state occupied at time *t* and show that ${\hat{\alpha }}_{t}^{\text{ls}}$, ${\hat{\beta }}_{t}^{\text{ls}}$ and ${\hat{\gamma }}_{t}^{\text{ls}}$ are closely related to the Aalen–Johansen estimator of the transition matrix. In this case, each element of ***Y***_*t*_−***Y***_*t* − 1_ equals 0, 1 or −1, and the variance of ***ε***_*t*_ depends on ***Y***_*t* − 1_. So, $\mathrm{Var}(\varepsilon_{t}|F_{t-1})=\mathrm{Var}(\varepsilon_{t})$ is not true. Even for a continuous outcome ***Y***_*t*_, it may not be true if ***Y***_*t*_ is bounded above or below.

### 4.2 Autoregressive MVN and no measurement error

Although the unstructured MVN method may be expected often to be more efficient than estimating the compensator or LI–LS imputation, this is not guaranteed, because unlike those methods it does not exploit the autoregressive assumption in Equation (2). As shown in Appendix S3, Equation (2) implies the following constraint on **Σ**: for 1≤*s*<*t*≤*T*,

(19) $$\begin{array}{cc} \Sigma_{s,t} & =\Sigma_{s,T+1}\Sigma_{T+1,T+1}^{-1}\Sigma_{T+1,t}\\ \;+\beta_{t}^{⊤}\beta_{t-1}^{⊤}\ldots \beta_{s+1}^{⊤}\left(\Sigma_{s,s}-\Sigma_{s,T+1}\Sigma_{T+1,T+1}^{-1}\Sigma_{T+1,s}\right) \end{array}$$

The model defined by ${\left(Y_{\;\;1}^{⊤},\ldots ,Y_{T}^{⊤},X^{⊤}\right)}^{⊤}∼\text{Normal}(\mu ,\Sigma )$ with the constraint of Equation (19) can be fitted by maximum likelihood to the observed data ignoring the missingness mechanism (an EM algorithm is described in Appendix S5). We call this the ‘autoregressive MVN’ method, and we call imputation using formula (18) with these estimates of ***μ*** and **Σ** ‘aMVN imputation’. The corresponding estimates of ***β***_*t*_, ***γ***_*t*_ and ***α***_*t*_ are given by Equations (15)–(17), and we call LI imputation using these estimates ‘LS ‐aMVN imputation’. These autoregressive MVN methods might be expected to be more efficient than the corresponding unstructured MVN methods.

Theorem 4 holds also for the autoregressive MVN method (proof in Appendix S1). Furthermore, the strong independent return assumption in that theorem can be replaced by the weaker independent return assumption. The following theorem shows a relation between autoregressive MVN and the methods of A&G.

Theorem 7 When data are monotone missing, the estimates of ***α***_*t*_, ***β***_*t*_ and ***γ***_*t*_ obtained by using autoregressive MVN followed by Equations (15)–(17) are equal to ${\hat{\alpha }}_{t}^{\text{ls}}$, ${\hat{\beta }}_{t}^{\text{ls}}$ and ${\hat{\gamma }}_{t}^{\text{ls}}$. Furthermore, LI–LS imputation, LI–aMVN imputation and aMVN imputation yield identical imputed values.

### 4.3 Random‐walk MVN and measurement error

Suppose now that there is measurement error ***e***_*t*_. Equation (2) implies that the underlying outcome ***Y***_*t*_ is conditionally independent of ***Y***_*s*_ for *s* < *t* − 1 given ***Y***_*t* − 1_ and ***X***, but it does not imply that the outcomes ***Y***_*t*_+***e***_*t*_ measured with error also have this autoregressive structure. Nevertheless, we show in Appendix S4 that when ***β***_*t*_=***I*** for all *t*, fitting the MVN model imposing the constraint of Equation (19) with ***β***_*t*_=***I*** (‘random‐walk MVN’ or ‘rMVN’) to the observed outcomes measured with error and ignoring the missingness mechanism gives consistent estimation of the parameters of Equation (2). The resulting LI–rMVN imputation and rMVN imputation methods can be more efficient than LI–LS imputation when data are non‐monotone missing.

## 5 Extension to higher‐order autoregression

Equation (2) implies that ***Y***_*t*_ is conditionally independent of ***Y***_1_,…,***Y***_*t* − 2_ given ***Y***_*t* − 1_. So, if it is assumed that there is no measurement error (***e***_*t*_=0), then the measured outcomes are assumed to follow a first‐order autoregressive process. This can be quite a restrictive assumption. When measurement error is allowed, Equation (2) implies that the measured outcome ***Y***_*t*_+***e***_*t*_ can, in general, depend on all previous measured outcomes, thus allowing more flexibility. Unfortunately, as shown in Section 2.3, LI methods that allow for measurement error require the restrictive assumption that ***β***_*t*_=***I***.

One way to allow an outcome measured at time *t* to depend on more than just the outcome measured at time *t* − 1, while still allowing ***β***_*t*_≠***I***, is to define ***Y***_*t*_ to include the outcome (or outcomes) measured at both times *t* and *t* − 1 and set ***e***_*t*_=0. This possibility was not explicitly mentioned by A&G but was mentioned in earlier work by the same authors (Gunnes et al., 2009a). There they considered only monotone missing data. We now describe how this approach could be used with monotone or non‐monotone missing data.

To avoid confusion, denote the outcome (or outcomes) of interest measured at time *t* as ***Z***_*t*_, define ***Y***_*t*_ in Equation (2) as $Y_{\;\;t}={\left(Z_{t}^{⊤},Z_{t-1}^{⊤}\right)}^{⊤}$ for *t* > 1 and ***Y***_1_=***Z***_1_, and let ***e***_*t*_=0 for all *t*. Equation (2) now allows for second‐order autoregression in ***Z***_*t*_.

Note that, by definition, *R*_0,*t*_=1 if and only if all elements of ***Y***_*t*_ are observed. (This was illustrated by A&G, who described an analysis where ***Y***_*t*_ included three quality‐of‐life outcomes all measured at time *t*; here *R*_0,*t*_=1 only when all three were observed.) When $Y_{\;\;t}={\left(Z_{t}^{⊤},Z_{t-1}^{⊤}\right)}^{⊤}$, *R*_0,*t*_=1 requires that both ***Z***_*t*_ and ***Z***_*t* − 1_ be observed. Because the two methods described by A&G only use values of ***Y***_*it*_ for which *R*_0*i*,*t*_=1, this means that if ***Z***_*it*_ is observed but ***Z***_*i*,*t* − 1_ and ***Z***_*i*,*t* + 1_ are both missing, then ***Z***_*it*_ is treated as missing and individual *i* is regarded as not having returned at time *t*. This is also true of the uMVN and aMVN methods. Furthermore, *R*_*t*_=1 if and only if ***Z***_*t*_, ***Z***_*t* − 1_ and ***Z***_*t* − 2_ are all observed. This means that if ***Z***_*it*_ and ***Z***_*i*,*t* + 1_ are observed but neither ***Z***_*i*,*t* − 1_ nor ***Z***_*i*,*t* + 2_ are, then A&G's methods do not use ***Z***_*it*_ or ***Z***_*i*,*t* + 1_ in the estimation of the parameters of Equation (2) (unlike the MVN methods).

Application of A&G's methods is now straightforward, except that it is not immediately obvious how LI–LS imputation should deal with an observed value of ***Z***_*it*_ when ***Z***_*i*,*t* − 1_ is missing and ***Z***_*i*,*t* + 1_ is observed. In this case, *R*_0*i*,*t*_=0 and *R*_0*i*,*t* + 1_=1. Because *R*_0*i*,*t*_=0, LI–LS imputation involves imputing ***Y***_*it*_ (and hence ***Z***_*it*_). However, because *R*_0*i*,*t* + 1_=1, ***Y***_*i*,*t* + 1_ (and hence ***Z***_*it*_) is observed. Thus, we have both an observed and an imputed value of ***Z***_*it*_. Fortunately, Theorem 5 implies that the conditional distributions of the observed and imputed values of ***Z***_*it*_ given the most recently observed value of ***Y***_*ik*_ (*k* < *t*) are the same. Therefore, it is valid to use either the observed or imputed value. We recommend using the former, for reasons of efficiency and robustness to possible misspecification of Equation (2).

Application of uMVN imputation is also straightforward. First, as with A&G's methods, any observed values of ***Z***_*it*_ for which ***Z***_*i*,*t* − 1_ and ***Z***_*i*,*t* + 1_ are both missing are deleted. One could then fit the MVN model to ${\left(Y_{\;\;1}^{⊤},\ldots ,Y_{T}^{⊤},X^{⊤}\right)}^{⊤}$. However, because the second element of ***Y***_*t* + 1_ and first element of ***Y***_*t*_ are equal by definition (both equal ***Z***_*t*_), there is no need to include both. Instead, one can just fit the model ${\left(Z_{1}^{⊤},\ldots ,Z_{T}^{⊤},X^{⊤}\right)}^{⊤}∼\text{Normal}(\mu^{Z},\Sigma^{Z})$. Missing values of ***Z***_*t*_ would then be imputed as their conditional expectations given the observed data, as in Equation ((18)). LI–uMVN imputation would instead involve calculating $\hat{\beta_{t}}$, $\hat{\gamma_{t}}$ and $\hat{\alpha_{t}}$ from the maximum likelihood estimates of ***μ*** ^*Z*^ and **Σ** ^*Z*^, as in Equations (15)–(17), and then using these to impute in the same way as in LI–LS imputation. LI–aMVN imputation and aMVN imputation could be adapted to allow for second‐order autoregression by imposing a constraint analogous to Equation (19) on **Σ**.

Extension to third‐ (or higher‐) order autoregression is possible, by defining ***Y***_*t*_=(***Z***_*t*_,***Z***_*t* − 1_,***Z***_*t* − 2_). However, the more elements ***Y***_*t*_ contains, the greater is the risk that one will be missing, causing more observed ***Z***_*t*_ values to be treated as missing.

## 6 Simulation studies

The following simple simulation studies are intended to demonstrate unbiasedness of the methods investigated in this paper when their assumptions are satisfied and biasedness when they are not, and to investigate their relative efficiencies. As indicated in Sections 2.6 and 4, estimating the compensator, LI–LS imputation, LI–aMVN imputation and aMVN imputation yield identical estimates when data are monotone missing. So, our simulation study scenarios were chosen to have high rates of dropout and return, in order to investigate how large the differences in efficiency can be when missingness is far from monotone. In most realistic situations, missingness would be non‐monotone but with lower rates of return, and so, the efficiency gains available there would be less than those demonstrated here. In the studies described in Sections 6.1 and 6.2, there is no measurement error. Appendix S8 includes a further study (Study 3) with measurement error and ***β***_*t*_=***I***. Its results demonstrate that methods that estimate ***β***_*t*_, rather than constraining it to equal its true value, are biased when there is measurement error, and also that, when data are non‐monotone missing, LI–rMVN imputation and rMVN imputation can be more efficient than LI–LS imputation with ***β***_*t*_ constrained as ***β***_*t*_=***I***.

### 6.1 Study 1: first‐order autoregression

Data were generated from the following model. Let *X* ∼ Uniform(0,1), *Y*_1_∼Normal(0.5*X*,1), *Y*_*t*_=0.4 + *β*_*t*_ *Y*_*t* − 1_+0.5*X* + *ε*_*t*_(*t* = 2,…6), *ε*_*t*_∼Normal(1,1) with probability 0.5 and *ε*_*t*_∼Normal(−1,1) otherwise, *R*_1_=1, $\text{logit}\{P(R_{0,t}=1|R_{0,t-1}=1,R_{0,t-2},F_{T})\}=\omega_{t}+X+(Y_{t-1}+Y_{t-2})/2$, and $\text{logit}\{P(R_{0,t}=1|R_{0,t-1}=0,R_{0,t-2},F_{T})\}=\phi_{t}+X+(Y_{L_{t}}+Y_{L_{t}-1})/2$, where *β*_*t*_=1.2 for all *t*, *L*_*t*_=argmax_*j*_{*j* < *t* and *R*_0,*j*_=1} (*t*≥2) denotes the most recent time at which the outcome was observed prior to time *t*, *L*_1_=1 and *Y*_0_=0. The values of *ω*_*t*_ and *ϕ*_*t*_ were chosen to make *P*(*R*_0,*t*_=0∣*R*_0,*t* − 1_=1) = 0.5 and *P*(*R*_0,*t*_=1∣*R*_0,*t* − 1_=0) = 0.5. This is a scenario in which dDTIC and strong independent return hold, and the data are MNAR. The (arguably unrealistic) bimodal distribution of *ε*_*t*_ was chosen to illustrate that normality of (*Y*_1_,…,*Y*_6_,*X*) is not required by the MVN methods.

For each of 1000 simulated datasets, we estimated *μ*_*t*_=*E*(*Y*_*t*_) by estimating the compensator and by LI–LS, LI–uMVN, LI–aMVN, uMVN and aMVN imputation. Estimates were also calculated from the complete data (i.e. before imposing missingness) and from the complete cases (i.e. the mean of the outcomes with *R*_0*t*_=1).

Table 1 shows the means and empirical SEs of the estimators. As expected, all but the complete‐cases estimator are approximately unbiased. LI imputation using the least‐squares estimates of *α*_*t*_, *β*_*t*_ and *γ*_*t*_ (LI–LS imputation) is considerably more efficient than estimating the compensator, and further efficiency is gained by replacing the least‐squares estimates by the corresponding parameter estimates from the unstructured or autoregressive MVN method (LI–uMVN or LI–aMVN imputation). Once *α*_*t*_, *β*_*t*_ and *γ*_*t*_ have been estimated, the LI imputation methods impute each individual's missing values using only his or her observed past. The uMVN and aMVN imputation methods, on the other hand, implicitly impute using the observed past and future. However, Table 1 shows that the efficiency gain from doing this is very small. It also shows that the difference in efficiency between the unstructured and autoregressive MVN methods is negligible.

**Table 1:** Means and empirical SEs of estimated *μ*_*t*_ in Simulation Study 1

Method	*μ*_1_	*μ*_2_	*μ*_3_	*μ*_4_	*μ*_5_	*μ*_6_
True values	0.250	0.950	1.790	2.798	4.008	5.459
	Means					
Complete data	0.249	0.945	1.784	2.790	3.998	5.447
Complete cases	0.249	1.482	2.471	4.164	6.009	8.325
Estim. compens.	0.249	0.944	1.784	2.798	4.017	5.469
LI–LS impute	0.249	0.944	1.786	2.796	4.007	5.455
LI–uMVN impute	0.249	0.946	1.786	2.790	3.997	5.454
LI–aMVN impute	0.249	0.946	1.787	2.788	3.999	5.450
uMVN impute	0.249	0.947	1.784	2.791	3.998	5.455
aMVN impute	0.249	0.947	1.786	2.789	4.000	5.450
	Empirical SEs					
Complete data	0.045	0.084	0.120	0.160	0.204	0.255
Complete cases	0.045	0.115	0.172	0.224	0.278	0.319
Estim. compens.	0.045	0.113	0.227	0.343	0.477	0.615
LI–LS impute	0.045	0.113	0.179	0.238	0.296	0.354
LI–uMVN impute	0.045	0.102	0.140	0.186	0.234	0.292
LI–aMVN impute	0.045	0.100	0.139	0.186	0.233	0.291
uMVN impute	0.045	0.099	0.137	0.182	0.231	0.292
aMVN impute	0.045	0.097	0.136	0.182	0.230	0.291

LI, linear increment; LS, least‐square; MVN, multivariate normal; aMVN, autoregressive MVN.

We also fitted the linear regression model *E*(*Y*_*t*_∣*X*) = *ψ*_0_+*ψ*_1_ *X* + *ψ*_2_ *t* + *ψ*_3_ *X* *t* to datasets imputed by the five imputation methods. The results were in agreement with the results of Table 1. Again, all but the complete‐case estimator (i.e. the least‐squares estimator using outcomes with *R*_0*t*_=1) are approximately unbiased; LI imputation using estimates of *α*_*t*_, *β*_*t*_ and *γ*_*t*_ from an MVN method is more efficient than LI imputation using the least‐squares estimates; and using the observed future to impute missing outcomes or constraining the variance matrix to be autoregressive provides little benefit (Table S1).

In Appendix S8, we present the results when the return mechanism *P*(*R*_0,*t*_=1∣*R*_0,*t* − 1_=1, $R_{0,t-2},F_{T})$ is modified so that the independent return assumption is violated. We demonstrate there that, as expected, estimating the compensator yields unbiased estimates of *E*(*Y*_*t*_) and *E*(*Y*_*t*_∣*X*), but the other methods (which assume independent return) are biased. Although the estimates from LI–LS imputation are biased, they are less biased than those from the MVN methods. This is because the least‐squares estimators of *α*_*t*_, *β*_*t*_ and ***γ***_*t*_ are unbiased, whereas the corresponding MVN‐based estimators are biased.

### 6.2 Study 2: second‐order autoregression

Data were generated from the following model. Let *X* ∼ Uniform(0,1), *Z*_1_∼Normal(0.5*X*,1), *Z*_2_=0.4 + 1.5*Z*_1_+0.5*X* + *ε*_2_, and *Z*_*t*_=0.4 + 1.5*Z*_*t* − 1_−0.5*Z*_*t* − 2_+0.5*X* + *ε*_*t*_(3≤*t*≤7), where *ε*_*t*_∼Normal(1,1) with probability 0.5 and *ε*_*t*_∼Normal(−1,1) otherwise. Let $R_{0,t}^{Z}=1$ if *Z*_*t*_ is observed, and $R_{0,t}^{Z}=0$ if *Z*_*t*_ is missing. *Z*_1_ is always observed.

Dropout is allowed at time 2 and when at least two consecutive outcomes have been observed. For times 4 and later, the probability of dropout depends on the rate of change in the outcome since time 2. Specifically, $\text{logit}\{P(R_{0,2}^{Z}=1|F_{T})\}=\omega_{2}+Z_{1}+X$, $\text{logit}\{P(R_{0,3}^{Z}=1|R_{0,2}^{Z}=1,F_{T})\}=\omega_{t}+W_{3}+X$ and $\text{logit}\{P(R_{0,t}^{Z}=1|R_{0,t-1}^{Z}=R_{0,t-2}^{Z}=1,R_{0,t-3}^{Z},F_{T})\}=\omega_{t}+W_{t}+X$ (4≤*t*≤7), where *W*_3_=*Z*_2_−*Z*_1_ and *W*_*t*_=(*Z*_*t* − 1_−*Z*_2_)/(*t* − 3) (4≤*t*≤7). Return is possible at times 3, 4, 5 and 6. Specifically, $\text{logit}\{P(R_{0,t}^{Z}=1|R_{0,t-1}^{Z}=0,R_{0,t-2}^{Z},F_{T})\}=\phi_{t}+X+W_{L_{t}^{Z}}$ (3≤*t*≤6), where $L_{t}^{Z}={\text{argmax}}_{j}\{j<t\text{and}R_{0,j}^{Z}=1\}$ denotes the most recent time at which the outcome *Z*_*t*_ was observed prior to time *t*. The value of *ω*_*t*_ (*ϕ*_*t*_) was chosen to make the overall probability of dropout (return) at time *t* among individuals at risk of dropout (return) at time *t* equal to 0.5. Note that dropout and return can depend on *Z*_2_, which may be missing, and that dDTIC and the independent return assumption hold for the process ***Y***_*t*_=(*Z*_*t*_,*Z*_*t* − 1_)^⊤^.

The results of applying the various methods with ***Y***_*t*_ defined as ***Y***_*t*_=(*Z*_*t*_,*Z*_*t* − 1_)^⊤^ are shown in Tables 2 and S2. The autoregressive MVN methods have not been applied, as these were shown in Simulation Study 1 to be hardly more efficient than the unstructured MVN methods. As expected, all but the complete‐case estimator are approximately unbiased, estimating the compensator is least efficient, followed by LI–LS imputation, and uMVN imputation is only slightly more efficient than LI–uMVN imputation.

**Table 2:** Means and empirical SEs of estimated *μ*_*t*_ in Simulation Study 2

Method	*μ*_1_	*μ*_2_	*μ*_3_	*μ*_4_	*μ*_5_	*μ*_6_	*μ*_7_
True values	0.250	1.025	2.062	3.231	4.466	5.733	7.016
	Means						
Complete data	0.247	1.022	2.059	3.230	4.465	5.733	7.014
Complete cases	0.247	1.702	3.066	3.911	5.589	7.256	12.222
Estim. compens.	0.247	1.022	2.065	3.237	4.466	5.735	7.015
LI–LS impute	0.247	1.022	2.065	3.233	4.462	5.731	7.010
LI–uMVN impute	0.247	1.022	2.061	3.229	4.463	5.729	7.014
uMVN impute	0.247	1.023	2.061	3.227	4.463	5.729	7.014
	Empirical SEs						
Complete data	0.046	0.095	0.148	0.188	0.226	0.262	0.293
Complete cases	0.046	0.125	0.197	0.239	0.286	0.326	0.557
Estim. compens.	0.046	0.126	0.258	0.480	0.672	0.809	0.925
LI–LS impute	0.046	0.126	0.206	0.275	0.304	0.330	0.428
LI–uMVN impute	0.046	0.113	0.164	0.205	0.245	0.282	0.386
uMVN impute	0.046	0.110	0.159	0.200	0.241	0.282	0.387

LI, linear increment; LS, least‐square; MVN, multivariate normal.

## 7 Missingness due to death

A&G briefly discuss inference in the situation where a cause of missingness is death. They distinguish between ‘immortal‐cohort’ and ‘mortal‐cohort’ inference (also known, respectively, as ‘unconditional’ and ‘partly conditional’ inference). In this section, we elaborate on their brief discussion, in particular, establishing that an additional, unmentioned assumption is required for mortal‐cohort inference. Like A&G, we restrict ourselves to the case of no measurement error, although similar considerations would apply if there were measurement error. We begin by modifying earlier assumptions about the outcome, dropout and return processes so that they make sense when data can be missing due to death. Then, we explain what is meant by immortal‐cohort and mortal‐cohort inference. This is followed by an example which illustrates that the conditions stated by A&G as being sufficient for valid LI imputation are insufficient when interest is in mortal‐cohort inference. Finally, we prove that these conditions can be made sufficient by adding an additional assumption about the death process.

Let *D*_*i*_ denote the time of individual's *i* last visit before death (*D*_*i*_=*T* if he or she does not die). Like A&G, we assume *D*_*i*_ is known for all individuals. For individuals with *D* < *t*, the variable ***Y***_*t*_ describes an outcome that, in general, does not exist. In this setting, the meanings of the assumptions expressed by Equations (2), (3), (4), (8), (9) and (12) are unclear. It is therefore difficult to assess whether they are plausible for any particular given dataset. We now show that they can be replaced by Equations (20)–(25), which are statements about only predeath outcomes, that is, outcomes that do exist.

Assume that

(20) $$Y_{\;\;\mathrm{it}}=\alpha_{t}+\beta_{t}^{⊤}Y_{\;\;i,t-1}+\gamma_{t}^{⊤}X_{\;i}+\varepsilon_{\mathrm{it}}\;(t=2,\ldots ,D_{i})$$

with $E(\varepsilon_{\mathrm{it}}|F_{i,t-1},D_{i}\geq t)=0$. This is a restriction of Equation (2) to predeath times, that is, to *t*≤*D*. Similarly, Equations (3), ((4)), ((8)), ((9)) and ((12)) are adapted to make them conditional on *t*≤*D*. Specifically, Equations (3), ((4)) and ((9)) are adapted to yield what we call the ‘mortal‐cohort weak DTIC’, ‘mortal‐cohort strong DTIC’ and ‘mortal‐cohort dDTIC’ assumptions, respectively:

(21) $$E(\Delta Y_{\;\;t}|F_{t-1},R_{t},D\geq t)=E(\Delta Y_{\;\;t}|F_{t-1},D\geq t)\;\;\forall t,$$

(22) $$E(Y_{\;\;t}|F_{t-1},R_{0,t},D\geq t)=E(Y_{\;\;t}|F_{t-1},D\geq t)\;\;\forall t,\text{and}$$

(23) $$f(Y_{\;\;t}|F_{t-1},R_{0,t},D\geq t)=f(Y_{\;\;t}|F_{t-1},D\geq t)\;\;\forall t,$$

while Equations ((8)) and ((12)) are adapted to yield the ‘mortal‐cohort independent return’ and ‘mortal‐cohort strong independent return’ assumptions, respectively:

(24) $$\begin{array}{cc} P(R_{0,t} & \;=\;1\;|\;R_{0,k-1},R_{0,k}\;=\;1,R_{0,k+1}\;=\;\ldots =R_{0,t-1}=0,X,Y_{\;\;k},Y_{\;\;k+1},\ldots Y_{\;\;t},D\geq t)\\ =P(R_{0,t}=1|R_{0,k-1},R_{0,k}=1,R_{0,k+1}=\ldots =R_{0,t-1}=0,X,Y_{\;\;k},D\geq t) \end{array}$$

and

(25) $$\begin{array}{cc} P(R_{0,t} & =1|R_{0,k-1},R_{0,k}=1,R_{0,k+1}=\ldots =R_{0,t-1}\\ =0,G_{k},Y_{\;\;k+1},Y_{\;\;k+2},\ldots Y_{\;\;t},D\geq t)\\ =P\left(R_{0,t}=1|R_{0,k-1},R_{0,k}=1,R_{0,k+1}=\ldots =R_{0,t-1}=0,G_{k},D\geq t\right). \end{array}$$

The stochastic process defined by Equation (20) terminates with ***Y***_*D*_. In immortal‐cohort inference, no distinction is made between data missing due to death and data missing for other reasons, and both are imputed in the same way. Consequently, immortal‐cohort inference is about *E*(***Y***_*t*_∣***Y***_1_,***X***) in the following ‘supplemented’ outcome process. Define the ‘supplemented process’ as that defined by Equation (20) up to time *D* but with additional hypothetical postdeath outcomes ***Y***_*D* + 1_,…,***Y***_*T*_ for individuals with *D* < *T* that are assumed to obey

$$\begin{array}{cc} f(\Delta Y_{\;\;t}|F_{t-1},R_{0,t},D<t) & =f(\Delta Y_{\;\;t}|F_{t-1},D<t)\\ =f(\Delta Y_{\;\;t}|F_{t-1},D\geq t)\;\;\forall t\geq D \end{array}$$

It thus follows from, respectively, Equations (21), (22) and (23) that $E\{\Delta Y_{\;\;t}|F_{t-1},R_{t},I(D\geq t)\}=E(\Delta Y_{\;\;t}|F_{t-1})$, that $E\{Y_{\;\;t}|F_{t-1},R_{0t},I(D\geq t)\}=E(Y_{\;\;t}|F_{t-1})$, and that

(26) $$f\{Y_{\;\;t}|F_{t-1},R_{0,t},I(D\geq t)\}=f(Y_{\;\;t}|F_{t-1}).$$

Equations ((2)), (3), ((4)) and ((9)) therefore hold for the supplemented process. Hence, the least‐squares estimators ${\hat{\alpha }}_{t}^{\text{ls}}$, ${\hat{\beta }}_{t}^{\text{ls}}$ and ${\hat{\gamma }}_{t}^{\text{ls}}$ of ***α***_*t*_, ***β***_*t*_ and ***γ***_*t*_ are unbiased and consistent for the parameters of Equation (2), and therefore also of Equation (20).

The proof in Section 3.3 of A&G establishes that LI–LS imputation respects $E(Y_{t}^{\text{est}}-Y_{\;\;t}|X,Y_{\;\;1})=0$ for the supplemented process, provided that Equations ((2)) and ((7)) and strong DTIC hold, and hence one can validly use the imputed data to estimate *E*(***Y***_*t*_∣***Y***_1_,***X***), the expected outcome in the supplemented process.

In mortal‐cohort inference, on the other hand, the estimand is *E*(***Y***_*t*_∣***Y***_1_,***X***,*D*≥*t*), the expected outcome at time *t* in individuals who are still alive at time *t*. A&G discuss immortal‐ versus mortal‐cohort inference. They (and Gunnes *et al.* (2009a)) defend immortal‐cohort inference, arguing that it may provide ‘a more fair comparison of treatments’ when one treatment improves survival in a way that means that patients with poor outcomes survive longer. However, Dufouil *et al.* (2004), Kurland & Heagerty (2005) and Kurland *et al.* (2009) generally favour mortal‐cohort inference, saying that for most purposes immortal‐cohort inference would be ‘inappropriate’, or that it is ‘generally inappropriate’ and ‘probably not of great scientific interest’ unless the death and outcome processes are independent.

A&G estimate *E*(***Y***_*t*_∣***Y***_1_,***X***,*D*≥*t*) by using LI–LS imputation to impute only predeath missing outcomes, that is, calculating $Y_{\;\;\mathrm{it}}^{\text{est}}$ for individual *i* only if *D*_*i*_≥*t*, and then fitting a linear regression model to the resulting imputed dataset $\left\{Y_{\;\;\mathrm{it}}^{\text{est}}:\;i=1,\ldots ,N;\;t\leq D_{i}\right\}$ (rather than $\left\{Y_{\;\;\mathrm{it}}^{\text{est}}:\;i=1,\ldots ,N;\;t=1,\ldots ,T\right\}$, as in Section 2.5). In order for this to be valid, it is necessary that $E(Y_{t}^{\text{est}}-Y_{\;\;t}|X,Y_{\;\;1},D\geq t)=0$. However, the fact that $E(Y_{t}^{\text{est}}-Y_{\;\;t}|X,Y_{\;\;1})=0$ for the supplemented process does not necessarily imply that $E(Y_{t}^{\text{est}}-Y_{\;\;t}|X,Y_{\;\;1},D\geq t)=0$. A simple example illustrates this. Suppose *T* = 3, *m* = 1 and there are no baseline covariates ***X***. Let *P*(*Y*_1_=0) = 1 and *P*(*Y*_2_=*Y*_3_=0) = *P*(*Y*_2_=*Y*_3_=1) = 0.5. Suppose that the only dropout occurs between times 1 and 2, and its probability does not depend on *Y*_2_, and that there is no return. Let *P*(*D* = 3∣*R*_0,2_=1) = *P*(*D* = 3∣*Y*_2_=0) = 1, so no one can die unless they drop out and have *Y*_2_=1. Let *P*(*D* = 2∣*R*_0,2_=0,*Y*_2_=1) = *P*(*D* = 3∣*R*_0,2_=0,*Y*_2_=1) = 0.5, so half of those who drop out and have *Y*_2_=1 die between times 2 and 3. When LI imputation or MVN imputation is applied to this population, the dropouts will all have their *Y*_3_ imputed as 0.5. However, among the dropouts who are still alive at time 3, the mean value of *Y*_3_ is actually 0.33.

Theorem 9 and its corollary, later, show that $E(Y_{t}^{\text{est}}-Y_{\;\;t}|X,Y_{\;\;1},D\geq t)=0$ can be ensured by making two additional assumptions. We call these ‘independent death’:

(27) $$\begin{array}{cc} P(D & =t|R_{0,k-1},R_{0,k}=1,R_{0,k+1}=\ldots =R_{0,t}=0,X,Y_{\;\;k},\ldots ,Y_{\;\;t},D\geq t)\\ =P(D=t|R_{0,k-1},R_{0,k}=1,R_{0,k+1}=\ldots =R_{0,t}=0,X,Y_{\;\;k},D\geq t) \end{array}$$

and ‘strong independent death’:

(28) $$\begin{array}{cc} P(D & \;=\;t\;|\;R_{0,k-1},R_{0,k}=1,R_{0,k+1}\;=\;\ldots =\;R_{0,t}\;=0,G_{k},Y_{\;\;k+1},Y_{\;\;k+2},\ldots ,Y_{\;\;t},D\geq t)\\ =P(D=t|R_{0,k-1},R_{0,k}=1,R_{0,k+1}=\ldots =R_{0,t}=0,G_{k},D\geq t) \end{array}$$

Equation ((27)) means that the probability of dying between visits *t* and *t* + 1 for people who did not attend visit *t* does not depend on outcomes since their last observed outcome. This is analogous to the independent return assumption (Equation (8)), except that independent return is about the probability of returning after dropout, rather than dying after dropout. Just as independent death is analogous to independent return, strong independent death is analogous to strong independent return. The independent death assumption will often not be entirely plausible; it is more likely to hold approximately when long gaps between dropout and death are uncommon.

Theorem 8 shows the relation between the mortal‐cohort independent return assumptions and independent death assumptions, and between mortal‐cohort assumptions and independent return assumptions in the supplemented process.

Theorem 8 Suppose that the increments model of Equation (20) and the mortal‐cohort dDTIC assumption of Equation (23) hold. Then, (a) mortal‐cohort strong independent return and strong independent death together imply mortal‐cohort independent return and independent death; (b) mortal‐cohort independent return and independent death together imply independent return in the supplemented process; and (c) mortal‐cohort strong independent return and strong independent death together imply strong independent return in the supplemented process.

Theorem 9 establishes that, under the conditions of Theorem 8, the conditional distribution of a missing outcome given earlier observed outcomes is the same whether or not the missing outcome is after death.

Theorem 9 If the increments model of Equation (20), and the mortal‐cohort dDTIC, mortal‐cohort independent return and independent death assumptions (Equations (23), ((24)) and ((28))) hold, then for *k* < *t*,

$$f(Y_{\;\;t}|R_{0,k-1},R_{0,k}=1,R_{0,k+1}=\ldots =R_{0,t}=0,X,Y_{\;\;k},D\geq t)=f(Y_{\;\;t}|X,Y_{\;\;k}),$$

Under the conditions of Theorem 9, if LI imputation is carried out using the true values of ***α***_*t*_, ***β***_*t*_ and ***γ***_*t*_, then $E(Y_{t}^{\text{est}}-{Y\;}_{t}|X,{Y\;}_{1},D\geq t)=0$.

In practice, the true values of ***α***_*t*_, ***β***_*t*_ and ***γ***_*t*_ are unknown and must be estimated. Earlier in this section, we described when the least‐squares estimators are unbiased and consistent. Now, we consider the uMVN and aMVN estimators.

(of Theorems 4 and 8) If the increments model of Equation (20) and the mortal‐cohort dDTIC, mortal‐cohort strong independent return and strong independent death assumptions (Equations (23), ((25)) and ((28))) hold and $\mathrm{Var}(\varepsilon_{t}|F_{t-1},D\;\geq \;t)\;=\;\mathrm{Var}(\varepsilon_{t}|D\;\geq \;t)$, then fitting the unstructured or autoregressive MVN model to the observed data ignoring the missingness mechanism yields consistent estimates of the mean and variance (***μ***,**Σ**) of the supplemented process, and hence of ***α***_*t*_, ***β***_*t*_ and ***γ***_*t*_. Furthermore, when fitting the autoregressive model, the mortal‐cohort strong independent return and strong independent death assumptions can be replaced by the mortal‐cohort independent return and independent death assumptions.

In Appendix S7, we discuss the validity of uMVN and aMVN imputation for mortal‐cohort inference. They are valid when ***ε***_*t*_ is normally distributed; otherwise, they are expected to have small bias. However, in view of the results in Section 6, where MVN imputation was only slightly more efficient than LI‐MVN imputation, the latter may be the better option.

## 8 Analysis of data from the BHPS

The BHPS began in 1991. The panel consisted of some 5500 households and 10,300 individuals drawn from many areas of Great Britain. Complex sampling weights were used to adjust for unequal selection probabilities and for non‐response. These weights were ignored in our analysis, which is intended to be illustrative rather than definitive. We examined the dependence of earnings (elicited for each year) on current age, adjusting for calendar time. We used waves 1–8 (1991–1998) (the period before booster samples was introduced) and restricted the sample to men aged 21–50 in 1991 who were observed to earn something during at least one of those 8 years. There were 3286 such men. Age was categorized as 21–25, 26–30, 31–40, 41–50 and 51–60. Mean earnings in each calendar year were estimated by estimating the compensator, LI–LS imputation and the MVN methods. Also, a linear regression model was fitted to the imputed dataset, regressing earnings in each year on current age group and calendar year. The baseline covariate ***X*** used in Equation (2) for all LI methods was age group in 1991. In order to handle missing values of earnings in 1991, we defined *Y*_*i*2_,…,*Y*_*i*9_ to be individual *i*'s incomes in 1991–1998, respectively, defined *Y*_*i*1_=*c* for all *i*, where *c* is an arbitrary constant, and constrained *β*_2_ in Equation (2) to equal zero (so the choice of *c* does not matter). SEs were estimated by bootstrapping.

Of the 3286 men, the percentage who had observed outcome at each wave varied from 55% (in 1983) to 64% (in 1987). 829 (15%) were observed at all eight waves, 658 (20%) had missing outcome at wave 1 but were observed at a later wave and did not drop out again, and 517 (16%) were observed at wave 1 but later dropped out and did not return. Of the remaining 1282 (39%) men, 976, 284 and 24 dropped out once, twice and thrice, respectively; and 1010, 258 and 14 returned once, twice and thrice, respectively. Of the 15,502 observed outcomes, 818 (5%) were immediately preceded and followed by missing outcomes; these are not used by the least‐squares estimators of the LI model and are completely ignored by LI methods that allow for autoregression of order two.

Table 3 shows the observed mean earnings in each year and the means estimated using seven LI methods. Estimates from LI–aMVN and LI–rMVN imputation (not shown) were very similar to those from LI–uMVN; estimates from aMVN and rMVN imputation (not shown) were very similar to those from uMVN imputation. All LI methods estimated the mean earnings in 1991 as lower than the observed mean. This is because the proportion of men reporting their earnings in 1991 increased with age group, and the mean earnings among those reporting also increased with age group, which suggests that the observed mean in 1991 is an overestimate of the mean in 1991 in the whole sample. Among the LI methods, the highest estimates came from estimating the compensator and the lowest from uMVN imputation. The difference ranged from 393 to 816 pounds. That these two methods differ most is not surprising, because the first makes no use of outcomes recorded immediately after return from dropout, whereas the second makes maximal use of these. Allowing for second‐order autoregression slightly increased estimates from LI–LS and LI–uMVN imputation but left those from estimating the compensator unchanged or slightly decreased.

**Table 3:** Estimated mean earnings (and SEs) in calendar years 1991–1998 (1000's of pounds).

	Year							
Method	1991	1992	1993	1994	1995	1996	1997	1998
	Estimated means							
Observed data	14.35	14.55	15.28	15.60	16.85	17.25	17.96	18.63
Compensator	13.87	14.45	15.10	15.84	16.72	17.46	18.57	19.24
LI–LS	13.87	14.33	14.91	15.49	16.45	17.12	17.92	18.51
LI–uMVN	13.73	14.18	14.75	15.31	16.26	16.93	17.76	18.40
uMVN	13.48	13.96	14.49	15.14	15.99	16.69	17.75	18.46
Compensator(2)	13.87	14.45	15.12	15.89	16.72	17.41	18.41	19.11
LI–LS(2)	13.87	14.44	15.03	15.65	16.58	17.25	18.08	18.80
LI–uMVN(2)	13.77	14.33	14.88	15.49	16.44	17.14	18.02	18.74
	Standard errors							
Observed data	0.240	0.228	0.250	0.263	0.321	0.283	0.321	0.352
Compensator	0.233	0.234	0.260	0.281	0.293	0.298	0.349	0.403
LI–LS	0.233	0.213	0.228	0.239	0.265	0.258	0.282	0.333
LI–uMVN	0.220	0.202	0.216	0.230	0.265	0.253	0.285	0.328
uMVN	0.209	0.192	0.208	0.228	0.248	0.252	0.280	0.331
Compensator(2)	0.233	0.234	0.266	0.291	0.301	0.315	0.336	0.420
LI–LS(2)	0.233	0.220	0.240	0.254	0.276	0.273	0.287	0.362
LI–uMVN(2)	0.224	0.209	0.226	0.241	0.276	0.267	0.297	0.360

Methods are means of observed data, estimating the compensator, LI–LS imputation, LI–uMVN imputation and uMVN imputation.

All LI methods use a LI model with autoregression of order one, unless they are marked ‘(2)’, in which case they use a LI model with second‐order autoregression.

LS, least‐square; LI, linear increments; MVN, multivariate normal.

Estimated standard errors from LI–aMVN imputation and LI–rMVN imputation were very close to those from LI–uMVN imputation. Those from aMVN imputation were very close to those from uMVN imputation; those from rMVN were up to 10% greater. The largest standard errors came from estimating the compensator; these were even larger than those for the observed means. Standard errors from LI–LS imputation were smaller than those for the observed means. Those from LI–uMVN imputation tended to be slightly smaller than those from LI–LS imputation, and those from uMVN imputation slightly smaller still. This suggests that when there is substantial dropout and return, methods (like LI–uMVN and uMVN) that use all the data efficiently can give appreciably more precise estimates. Allowing for second‐order autoregression generally increased the standard errors, because this involves ignoring some of the observed outcomes.

Table 4 shows estimated coefficients when the linear regression of earnings on current age group and calendar year was fitted to observed data and to data imputed by the various LI methods. Mean earnings increased with current age, being 3000–4000, 6500–7500 and 7000–8000 pounds higher at ages 26–30, 31–40 and 41–50, respectively, than at 21–25. However, they were 1500–2500 pounds lower at ages 51–60 than at 41–50, possibly because of early retirement. The LI imputation methods all estimated that the differences between ages 26–50 and ages 21–25 were somewhat lower that the observed data suggest. Standard errors for LI methods were lower than those for the observed‐data analysis. Those for LI–uMVN and uMVN imputation were lower than those for LI–LS imputation. Allowing for second‐order autoregression increased standard errors, probably because some observed outcomes are then ignored.

**Table 4:** Estimated coefficients (and standard errors) of linear regression of earnings (1000's of pounds) on current age group and year.

		Current age				Year
Method	Intercept	26–30	31–40	41–50	51–60	–1991
Estimates						
Observed data	8.322	4.018	7.386	7.948	5.472	0.475
LI–LS	8.517	3.879	7.109	7.774	5.792	0.457
LI–uMVN	8.557	3.614	6.855	7.519	5.496	0.468
uMVN	8.404	3.386	6.641	7.396	5.330	0.512
LI–LS(2)	8.504	3.783	7.236	7.907	6.127	0.473
LI–uMVN(2)	8.573	3.596	6.960	7.615	5.722	0.494
Standard errors						
Observed data	0.322	0.377	0.459	0.525	0.799	0.049
LI–LS	0.298	0.335	0.436	0.520	0.713	0.046
LI–uMVN	0.284	0.315	0.422	0.496	0.691	0.045
uMVN	0.298	0.323	0.433	0.486	0.686	0.042
LI–LS(2)	0.300	0.338	0.447	0.541	0.764	0.047
LI.uMVN(2)	0.289	0.319	0.435	0.516	0.729	0.047

Methods are observed data, LI–LS imputation, LI–uMVN imputation and uMVN imputation. LI methods use a LI model with autoregression of order one, unless they are marked ‘(2)’, in which case they use a LI model with second‐order autoregression.

LI, linear increment; LS, least‐square; MVN, multivariate normal.

## 9 Discussion

Missing at random and the distributional form of DTIC (dDTIC) are equivalent assumptions when missingness is monotone and there is no independent measurement error. Otherwise, they differ. MAR allows dropout and return to depend on observed outcomes, including future outcomes but not on the underlying (error‐free) outcomes. DTIC allows dropout and return to depend on *all* past underlying outcomes but not on future outcomes or on the measurement error. Nevertheless, we have shown that some methods that ostensibly assume MAR can also be valid when data are MNAR but dDTIC holds and the underlying outcomes have an autoregressive structure. For example, in the absence of measurement error, fitting an unstructured MVN model and ignoring the missingness mechanism gives consistent estimation and can be more efficient than LI imputation using least squares (even when data are not normally distributed). In addition, it remains valid under the alternative assumption of MAR, even if the autoregressive structure does not apply. On the other hand, LI using least squares allows the variance of ***ε***_*t*_ to depend on the history $F_{i,t-1}$, which may make this method more appealing than the MVN method when the outcome is categorical. Moreover, it is less prone to bias than the MVN method when the independent return assumption is violated. Estimating the compensator is the least efficient method but makes the fewest assumptions: it only requires weak DTIC and not independent return.

The MVN method can be made more efficient by constraining the variance matrix to have an autoregressive form. However, this was found in simulation studies to offer little benefit. Further investigation is required to determine whether it offers greater benefit with small sample sizes or when there are many time points.

As we have shown, when there is measurement error, none of the methods discussed in this paper allow the increment Δ***Y***_*t*_ to depend on underlying outcome ***Y***_*t* − 1_ (i.e. for ***β***_*t*_≠***I***), unless the magnitude of this dependence (i.e. the value of ***β***_*t*_) is known, which is unlikely in practice. Just as measurement error can be handled when ***β***_*t*_ is known, it may be possible when ***β***_*t*_ is unknown but the measurement error variance, Var(***e***_*t*_), is known (Fuller, 1987).

Like A&G, we have focused on a first‐order autoregressive model. This is quite a restrictive assumption and may be too restrictive for some applications. So, we have shown in detail how the various methods can allow for autoregression of order *s* > 1 by including in the vector ***Y***_*t*_ not only the underlying outcome at time *t* but also those at times *t* − 1,…,*t* − *s* + 1. A drawback of this approach is that because *R*_0,*t*_=0 when ***Y***_*it*_ is not fully observed, some observed measurements may be ignored unless missingness is monotone. Alternative, more efficient extensions may be possible.

Software for applying some of the methods covered in this paper are described in Appendix S9. Like DF&H and A&G, we recommend using bootstrap to calculate standard errors.. However, it may be possible to derive expressions for sandwich variance estimators (Zeng & Lin, 2007; Farewell, 2010).

Note that we have treated the missingness processes of individuals as independent, whereas A&G's formulation is slightly more general, allowing for dropout and return of one individual to depend on the histories of other individuals.

A&G briefly discuss how LI could be used for causal inference about treatment effects in a non‐randomized longitudinal study with time‐dependent confounders, viewing the counterfactual untreated outcomes of treated individuals as missing data.

Apart from estimating the compensator, all methods discussed in this article require an assumption about the probability of return after dropout (the independent return assumption). Much of the published work on non‐monotone missing data in longitudinal studies assumes either MAR or that the probability of attending a visit at time *t* is independent of attendance at other times given the outcomes or given a random effect shared with the model for the outcomes, and so does not focus explicitly on return. Liao *et al.* (2012) give a recent review of some of this work. Articles in which an explicit model for the probability of return is used include Preisser *et al.* (2000), Lin *et al.* (2004) and Liao *et al.* (2012).

It would be interesting to understand better the connection between the random‐walk MVN method and the g‐inverse working singularity GEE method proposed by Farewell (2010). When ***β***_*t*_=***I*** and there are no covariates ***X***, Equation (19) reduces to **Σ**_*s*,*t*_=**Σ**_*s*,*s*_ for all *s* < *t*. A special case of this is **Σ**_*s*,*t*_=**Σ**_*s*,*s*_=*s*, which is the working covariance matrix used by Farewell (2010). Because this is the covariance matrix of a random walk where all increments have unit variance, it may be that the random‐walk MVN method is more efficient than the g‐inverse method when the variances of the increments differ at different times.

Finally, we note that we have not discussed the plausibility of the MAR and DTIC assumptions. This must be considered within the context of any given dataset. For example, medical data may be MAR when follow‐up of a patient is determined by the doctor on the basis of a measured health outcome, whereas DTIC may be more plausible when follow‐up is determined by the patient on the basis of his or her underlying health state. When data are monotone missing, MAR has a straightforward interpretation: dropout is independent of the present and future given the past. Interpretation when data are non‐monotone missing is more problematic, however (Robins & Gill, 1997). One advantage of the DTIC assumption may be that it is easily interpreted in both the monotone and non‐monotone case.

## Supporting information

Supporting info item
