# Supplementary material for: Linear Increments with Non‐monotone Missing Data and Measurement Error
Source: Scand Stat Theory Appl. 2016 Apr 6;43(4):996–1018. doi: 10.1111/sjos.12225 (PMC5111617; doi:10.1111/sjos.12225)
Supplement: Supplementary file 1 — Supporting info item [file SJOS-43-996-s001.pdf]

Supporting Materials: Proofs of theorems, further results from simulation study, and EM and Newton-Raphson algorithms

Appendix S1: Proofs of Theorems

In all the following proofs, let $A_{k,t}$ denote the event $\{R_{0,k} = 1, R_{0,k+1} = R_{0,k+2} = \ldots = R_{0,t} = 0\}$. Let $\bar{\boldsymbol{Y}}_t$ denote the complete-data sample mean of $\boldsymbol{Y}_t$ and $\bar{\boldsymbol{X}}$ denote the complete-data sample mean of $\boldsymbol{X}$. Also, let $\boldsymbol{c}_{s,t}$ $(1 \leq s, t \leq T)$ denote the complete-data sample covariance of $\boldsymbol{Y}_s$ and $\boldsymbol{Y}_t$, let $\boldsymbol{c}_{T+1,t}$ denote the sample covariance of $\boldsymbol{X}$ and $\boldsymbol{Y}_t$, and let $\boldsymbol{c}_{T+1,T+1}$ denote the sample variance of $\boldsymbol{X}$.

1.1 Proof of Theorem 1

When $\hat{\boldsymbol{\beta}}_t^{\mathrm{ls}}$ is fixed to equal $\boldsymbol{\beta}_t$, the least-squares estimator $(\hat{\boldsymbol{\alpha}}_t^{\mathrm{ls}}, \hat{\boldsymbol{\gamma}}_t^{\mathrm{ls}})$ of the remaining parameters $(\boldsymbol{\alpha}_t^{\mathrm{ls}}, \boldsymbol{\gamma}_t^{\mathrm{ls}})$ using only those individuals with $R_t = 1$ is given by

$$\begin{bmatrix} \hat{\boldsymbol{\alpha}}_t^{\mathrm{ls}\top} \\ \hat{\boldsymbol{\gamma}}_t^{\mathrm{ls}} \end{bmatrix} = (\boldsymbol{U}_t^\top \boldsymbol{U}_t)^{-1} \boldsymbol{U}_t^\top \begin{bmatrix} \boldsymbol{Y}_{1t}^\top + \boldsymbol{e}_{1t}^\top - (\boldsymbol{Y}_{1,t-1} + \boldsymbol{e}_{1,t-1})^\top \boldsymbol{\beta}_t \\ \vdots \\ \boldsymbol{Y}_{Nt}^\top + \boldsymbol{e}_{Nt}^\top - (\boldsymbol{Y}_{N,t-1} + \boldsymbol{e}_{N,t-1})^\top \boldsymbol{\beta}_t \end{bmatrix}$$

where

$$\boldsymbol{U}_t = \begin{bmatrix} R_{1t} & R_{1t}\boldsymbol{X}_1^\top \\ \vdots & \vdots \\ R_{Nt} & R_{Nt}\boldsymbol{X}_N^\top \end{bmatrix}$$

Therefore, assuming that equations (2) and (3) hold, that the measurement error process is independent of the other processes, and that measurement errors have

mean zero, we have

$$
\begin{aligned}
&E\left(\left[\begin{array}{c}(\hat{\boldsymbol{\alpha}}_t^{\mathrm{ls}})^\top\\ \hat{\gamma}_t^{\mathrm{ls}}\end{array}\right] \mid \{\mathcal{F}_{it-1},\mathcal{R}_{it}: i=1,\ldots,N\}\right)\\
&=(\boldsymbol{U}_t^\top\boldsymbol{U}_t)^{-1}\boldsymbol{U}_t^\top\left\{E\left(\left[\begin{array}{c}\boldsymbol{Y}_{1t}^\top\\ \vdots\\ \boldsymbol{Y}_{Nt}^\top\end{array}\right] \mid \{\mathcal{F}_{it-1},\mathcal{R}_{it}: i=1,\ldots,N\}\right)-\left[\begin{array}{c}\boldsymbol{Y}_{1,t-1}^\top\boldsymbol{\beta}_t\\ \vdots\\ \boldsymbol{Y}_{N,t-1}^\top\boldsymbol{\beta}_t\end{array}\right]\right\}\\
&=(\boldsymbol{U}_t^\top\boldsymbol{U}_t)^{-1}\boldsymbol{U}_t^\top\left\{\left[\begin{array}{c}\boldsymbol{\alpha}_t^\top+\boldsymbol{Y}_{1,t-1}^\top\boldsymbol{\beta}_t+\boldsymbol{X}_1^\top\boldsymbol{\gamma}_t\\ \vdots\\ \boldsymbol{\alpha}_t^\top+\boldsymbol{Y}_{N,t-1}^\top\boldsymbol{\beta}_t+\boldsymbol{X}_N^\top\boldsymbol{\gamma}_t\end{array}\right]-\left[\begin{array}{c}\boldsymbol{Y}_{1,t-1}^\top\boldsymbol{\beta}_t\\ \vdots\\ \boldsymbol{Y}_{N,t-1}^\top\boldsymbol{\beta}_t\end{array}\right]\right\}\\
&=(\boldsymbol{U}_t^\top\boldsymbol{U}_t)^{-1}\boldsymbol{U}_t^\top\left[\begin{array}{c}\boldsymbol{\alpha}_t^\top+\boldsymbol{X}_1^\top\boldsymbol{\gamma}_t\\ \vdots\\ \boldsymbol{\alpha}_t^\top+\boldsymbol{X}_N^\top\boldsymbol{\gamma}_t\end{array}\right]\\
&=(\boldsymbol{U}_t^\top\boldsymbol{U}_t)^{-1}\boldsymbol{U}_t^\top\boldsymbol{U}_t\left[\begin{array}{c}\hat{\boldsymbol{\alpha}}_t^\top\\ \hat{\boldsymbol{\gamma}}_t\end{array}\right]\\
&=\left[\begin{array}{c}\hat{\boldsymbol{\alpha}}_t^\top\\ \hat{\boldsymbol{\gamma}}_t\end{array}\right]
\end{aligned}
\tag{29}
$$

Hence

$$
E\left\{\left[\begin{array}{c}(\hat{\boldsymbol{\alpha}}_t^{\mathrm{ls}})^\top\\ \hat{\boldsymbol{\gamma}}_t^{\mathrm{ls}}\end{array}\right]\right\}=\left[\begin{array}{c}\boldsymbol{\alpha}_t^\top\\ \boldsymbol{\gamma}_t^\top\end{array}\right]
$$

For consistency of $(\boldsymbol{\alpha}_t^{\mathrm{ls}},\boldsymbol{\gamma}_t^{\mathrm{ls}})$, we see that

$$
\begin{aligned}
&\mathrm{Var}\left(\left[\begin{array}{c}(\hat{\boldsymbol{\alpha}}_t^{\mathrm{ls}})^\top\\ \hat{\boldsymbol{\gamma}}_t^{\mathrm{ls}}\end{array}\right]\right)\\
&=\mathrm{Var}\left\{E\left(\left[\begin{array}{c}(\hat{\boldsymbol{\alpha}}_t^{\mathrm{ls}})^\top\\ \hat{\boldsymbol{\gamma}}_t^{\mathrm{ls}}\end{array}\right] \mid \{\mathcal{F}_{it-1},\mathcal{R}_{it}: i=1,\ldots,N\}\right)\right\}\\
&\quad+E\left\{\mathrm{Var}\left(\left[\begin{array}{c}(\hat{\boldsymbol{\alpha}}_t^{\mathrm{ls}})^\top\\ \hat{\boldsymbol{\gamma}}_t^{\mathrm{ls}}\end{array}\right] \mid \{\mathcal{F}_{it-1},\mathcal{R}_{it}: i=1,\ldots,N\}\right)\right\}\\
&=E\left\{\mathrm{Var}\left(\left[\begin{array}{c}(\hat{\boldsymbol{\alpha}}_t^{\mathrm{ls}})^\top\\ \hat{\boldsymbol{\gamma}}_t^{\mathrm{ls}}\end{array}\right] \mid \{\mathcal{F}_{it-1},\mathcal{R}_{it}: i=1,\ldots,N\}\right)\right\}\\
&=E\left\{(\boldsymbol{U}_t^\top\boldsymbol{U}_t)^{-1}\boldsymbol{U}_t^\top\mathrm{Var}\left(\left[\begin{array}{c}\boldsymbol{Y}_{1t}^\top+\boldsymbol{e}_{1t}^\top\\ \vdots\\ \boldsymbol{Y}_{Nt}^\top+\boldsymbol{e}_{Nt}^\top\end{array}\right] \mid \{\mathcal{F}_{it-1},\mathcal{R}_{it}: i=1,\ldots,N\}\right)\boldsymbol{U}_t(\boldsymbol{U}_t^\top\boldsymbol{U}_t)^{-1}\right\}\\
&=E\left[(\boldsymbol{U}_t^\top\boldsymbol{U}_t)^{-1}\boldsymbol{U}_t^\top\mathrm{diag}\{\mathrm{Var}(\boldsymbol{\epsilon}_t)+\mathrm{Var}(\boldsymbol{e}_t)\}\boldsymbol{U}_t(\boldsymbol{U}_t^\top\boldsymbol{U}_t)^{-1}\right]\\
&\to 0 \text{ as } N\to\infty
\end{aligned}
$$

Similarly to equation (29), it can be shown that when equation (4) holds,

$$
E\left\{\left[\begin{array}{c}(\hat{\boldsymbol{\alpha}}_t^{\mathrm{ls}})^\top\\ \hat{\boldsymbol{\gamma}}_t^{\mathrm{ls}}\end{array}\right] \mid \{\mathcal{F}_{it-1},\mathcal{R}_{0,it}: i=1,\ldots,N\}\right\}=\left[\begin{array}{c}\boldsymbol{\alpha}_t^\top\\ \boldsymbol{\gamma}_t\end{array}\right]
\tag{30}
$$

1.2 Proof of Theorem 2

In this proof we omit the superscript 'ls' from $\boldsymbol{\alpha}_t^{\text{ls}}$ and $\boldsymbol{\gamma}_t^{\text{ls}}$.

In their Section 3.3, A&G describe their imputation method. Adapting their formulae for $\boldsymbol{Y}_t^{\text{est}}$ and $\Delta\boldsymbol{Y}_t^{\text{est}}$ to make them apply to the outcomes observed with error rather than to the underlying outcomes, we have

$$\begin{aligned}
\boldsymbol{Y}_1^{\text{est}} &= \boldsymbol{Y}_1 + \boldsymbol{e}_1 \\
\Delta\boldsymbol{Y}_t^{\text{est}} &= (1 - R_{0t})(\hat{\boldsymbol{\alpha}}_t + \hat{\boldsymbol{\gamma}}_t^{\top}\boldsymbol{X}) + R_{0t}(\boldsymbol{Y}_t + \boldsymbol{e}_t - \boldsymbol{Y}_{t-1}^{\text{est}}) \\
\boldsymbol{Y}_t^{\text{est}} &= \boldsymbol{Y}_{t-1}^{\text{est}} + \Delta\boldsymbol{Y}_t^{\text{est}}
\end{aligned}$$

We use induction to prove that $E(\boldsymbol{Y}_t^{\text{est}} - \boldsymbol{Y}_t \mid \boldsymbol{X}, \boldsymbol{Y}_1) = \boldsymbol{0}$ for all $t = 1, \ldots, T$. Assume that

$$E(\boldsymbol{Y}_{t-1}^{\text{est}} - \boldsymbol{Y}_{t-1} \mid \boldsymbol{X}, \boldsymbol{Y}_1) = \boldsymbol{0}. \tag{31}$$

This is true for $t-1=1$, since $E(\boldsymbol{e}_1 \mid \boldsymbol{X}, \boldsymbol{Y}_1) = \boldsymbol{0}$. Now, for $t \geq 2$,

$$
\begin{aligned}
& E(\boldsymbol{Y}_t^{\text{est}} - \boldsymbol{Y}_t \mid \boldsymbol{X}, \boldsymbol{Y}_1) \\
&= E(\Delta \boldsymbol{Y}_t^{\text{est}} - \Delta \boldsymbol{Y}_t \mid \boldsymbol{X}, \boldsymbol{Y}_1) + E(\boldsymbol{Y}_{t-1}^{\text{est}} - \boldsymbol{Y}_{t-1} \mid \boldsymbol{X}, \boldsymbol{Y}_1) \\
&= E(\Delta \boldsymbol{Y}_t^{\text{est}} - \Delta \boldsymbol{Y}_t \mid \boldsymbol{X}, \boldsymbol{Y}_1) && (32) \\
&= E\{(1-R_{0t})(\hat{\boldsymbol{\alpha}}_t + \hat{\boldsymbol{\gamma}}_t^\top \boldsymbol{X}) + R_{0t}(\boldsymbol{Y}_t + \boldsymbol{e}_t - \boldsymbol{Y}_{t-1}^{\text{est}}) - \Delta \boldsymbol{Y}_t \mid \boldsymbol{X}, \boldsymbol{Y}_1\} \\
&= E\{(1-R_{0t})(\hat{\boldsymbol{\alpha}}_t + \hat{\boldsymbol{\gamma}}_t^\top \boldsymbol{X}) \mid \boldsymbol{X}, \boldsymbol{Y}_1\} \\
&\quad + E\{R_{0t}(\boldsymbol{Y}_{t-1} + \Delta \boldsymbol{Y}_t + \boldsymbol{e}_t - \boldsymbol{Y}_{t-1}^{\text{est}}) - \Delta \boldsymbol{Y}_t \mid \boldsymbol{X}, \boldsymbol{Y}_1\} \\
&= E\{(1-R_{0t})(\hat{\boldsymbol{\alpha}}_t + \hat{\boldsymbol{\gamma}}_t^\top \boldsymbol{X}) \mid \boldsymbol{X}, \boldsymbol{Y}_1\} + E\{R_{0t}(\Delta \boldsymbol{Y}_t + \boldsymbol{e}_t) - \Delta \boldsymbol{Y}_t \mid \boldsymbol{X}, \boldsymbol{Y}_1\} && (33) \\
&= E\{(1-R_{0t})(\hat{\boldsymbol{\alpha}}_t + \hat{\boldsymbol{\gamma}}_t^\top \boldsymbol{X}) \mid \boldsymbol{X}, \boldsymbol{Y}_1\} + E\{R_{0t}\Delta \boldsymbol{Y}_t - \Delta \boldsymbol{Y}_t \mid \boldsymbol{X}, \boldsymbol{Y}_1\} && (34) \\
&= E\{(1-R_{0t})(\hat{\boldsymbol{\alpha}}_t + \hat{\boldsymbol{\gamma}}_t^\top \boldsymbol{X}) \mid \boldsymbol{X}, \boldsymbol{Y}_1\} - E\{(1-R_{0t})\Delta \boldsymbol{Y}_t \mid \boldsymbol{X}, \boldsymbol{Y}_1\} \\
&= E\{(1-R_{0t})(\hat{\boldsymbol{\alpha}}_t + \hat{\boldsymbol{\gamma}}_t^\top \boldsymbol{X}) \mid \boldsymbol{X}, \boldsymbol{Y}_1\} \\
&\qquad - E[E\{(1-R_{0t})\Delta \boldsymbol{Y}_t \mid R_{0t}, \boldsymbol{X}, \boldsymbol{Y}_1, \ldots, \boldsymbol{Y}_{t-1}\} \mid \boldsymbol{X}, \boldsymbol{Y}_1] \\
&= E\{(1-R_{0t})(\hat{\boldsymbol{\alpha}}_t + \hat{\boldsymbol{\gamma}}_t^\top \boldsymbol{X}) \mid \boldsymbol{X}, \boldsymbol{Y}_1\} \\
&\quad - E[(1-R_{0t})E\{\Delta \boldsymbol{Y}_t \mid \boldsymbol{X}, \boldsymbol{Y}_1, \ldots, \boldsymbol{Y}_{t-1}\} \mid \boldsymbol{X}, \boldsymbol{Y}_1] && (35) \\
&= E\{(1-R_{0t})(\hat{\boldsymbol{\alpha}}_t + \hat{\boldsymbol{\gamma}}_t^\top \boldsymbol{X}) \mid \boldsymbol{X}, \boldsymbol{Y}_1\} - E[(1-R_{0t})(\boldsymbol{\alpha}_t + \boldsymbol{\gamma}_t^\top \boldsymbol{X}) \mid \boldsymbol{X}, \boldsymbol{Y}_1] \\
&= E(E[(1-R_{0t})(\hat{\boldsymbol{\alpha}}_t + \hat{\boldsymbol{\gamma}}_t^\top \boldsymbol{X}) \mid \{\mathcal{F}_{it-1}, \mathcal{R}_{0,it} : i = 1, \ldots, N\}] \mid \boldsymbol{X}, \boldsymbol{Y}_1) \\
&\qquad - E[(1-R_{0t})(\boldsymbol{\alpha}_t + \boldsymbol{\gamma}_t^\top \boldsymbol{X}) \mid \boldsymbol{X}, \boldsymbol{Y}_1] \\
&= E[(1-R_{0t})E(\hat{\boldsymbol{\alpha}}_t + \hat{\boldsymbol{\gamma}}_t^\top \boldsymbol{X} \mid \{\mathcal{F}_{it-1}, \mathcal{R}_{0,it} : i = 1, \ldots, N\}) \mid \boldsymbol{X}, \boldsymbol{Y}_1] \\
&\qquad - E[(1-R_{0t})(\boldsymbol{\alpha}_t + \boldsymbol{\gamma}_t^\top \boldsymbol{X}) \mid \boldsymbol{X}, \boldsymbol{Y}_1] \\
&= E\{(1-R_{0t})(\boldsymbol{\alpha}_t + \boldsymbol{\gamma}_t^\top \boldsymbol{X}) \mid \boldsymbol{X}, \boldsymbol{Y}_1\} \\
&\quad - E\{(1-R_{0t})(\boldsymbol{\alpha}_t + \boldsymbol{\gamma}_t^\top \boldsymbol{X}) \mid \boldsymbol{X}, \boldsymbol{Y}_1\} && (36) \\
&= \boldsymbol{0} && (37)
\end{aligned}
$$

Equation (32) follows by equation (31). Equation (33) follows by using equation (7). Equation (34) follows by the independence of measurement errors. Equation (35) follows by using equation (4). Equation (36) follows by using equa-

tion (30).

1.3 Proof of Theorem 3

$$
\begin{aligned}
&P(R_{0,t}=1 \mid \mathcal{R}_{0,k-1}, A_{k,t-1}, \boldsymbol{C}_k, \boldsymbol{Y}_{k+1}, \ldots \boldsymbol{Y}_t) \\
&= \int P(R_{0,t}=1 \mid \mathcal{R}_{0,k-1}, A_{k,t-1}, \boldsymbol{B}_k, \boldsymbol{Y}_{k+1}, \ldots, \boldsymbol{Y}_t) \\
&\qquad \times f(\boldsymbol{B}_k \mid \mathcal{R}_{0,k-1}, A_{k,t-1}, \boldsymbol{C}_k, \boldsymbol{Y}_{k+1}, \ldots \boldsymbol{Y}_t)\, d\boldsymbol{B}_k \\
&= \int P(R_{0,t}=1 \mid \mathcal{R}_{0,k-1}, A_{k,t-1}, \boldsymbol{B}_k) \\
&\qquad \times f(\boldsymbol{B}_k \mid \mathcal{R}_{0,k-1}, A_{k,t-1}, \boldsymbol{C}_k, \boldsymbol{Y}_{k+1}, \ldots \boldsymbol{Y}_t)\, d\boldsymbol{B}_k \\
&= \int P(R_{0,t}=1 \mid \mathcal{R}_{0,k-1}, A_{k,t-1}, \boldsymbol{B}_k)\, f(\boldsymbol{B}_k \mid \mathcal{R}_{0,k-1}, A_{k,t-1}, \boldsymbol{C}_k) \\
&\qquad \times \frac{f(\boldsymbol{Y}_{k+1}, \ldots, \boldsymbol{Y}_t \mid \mathcal{R}_{0,k-1}, A_{k,t-1}, \boldsymbol{B}_k)}{f(\boldsymbol{Y}_{k+1}, \ldots, \boldsymbol{Y}_t \mid \mathcal{R}_{0,k-1}, A_{k,t-1}, \boldsymbol{C}_k)}\, d\boldsymbol{B}_k \\
&= \int P(R_{0,t}=1 \mid \mathcal{R}_{0,k-1}, A_{k,t-1}, \boldsymbol{B}_k) \\
&\qquad \times f(\boldsymbol{B}_k \mid \mathcal{R}_{0,k-1}, A_{k,t-1}, \boldsymbol{C}_k)\, d\boldsymbol{B}_k \qquad (38) \\
&= P(R_{0,t}=1 \mid \mathcal{R}_{0,k-1}, A_{k,t-1}, \boldsymbol{C}_k)
\end{aligned}
$$

Line (38) follows because of the assumption of dDTIC and the autoregressive assumption of equation (1), as we now show.

For $t > k$,

$$
\begin{aligned}
&f(\boldsymbol{Y}_{k+1}, \ldots, \boldsymbol{Y}_t \mid \mathcal{R}_{0,k-1}, A_{k,t-1}, \boldsymbol{B}_k) \\
&= \prod_{s=k+1}^{t} f(\boldsymbol{Y}_s \mid \mathcal{R}_{0,k-1}, A_{k,t-1}, \boldsymbol{B}_k, \boldsymbol{Y}_{k+1}, \ldots, \boldsymbol{Y}_{s-1}) \qquad (39)
\end{aligned}
$$

For $k < s \leq t$,

$$
\begin{aligned}
&f(\boldsymbol{Y}_s \mid \mathcal{R}_{0,k-1}, A_{k,t-1}, \boldsymbol{B}_k, \boldsymbol{Y}_{k+1}, \ldots, \boldsymbol{Y}_{s-1}) \\
&= f(\boldsymbol{Y}_s \mid \mathcal{R}_{0,k-1}, A_{k,s}, \boldsymbol{B}_k, \boldsymbol{Y}_{k+1}, \ldots, \boldsymbol{Y}_{s-1}) \\
&\qquad \times \frac{P(A_{k,t-1} \mid \mathcal{R}_{0,k-1}, A_{k,s}, \boldsymbol{B}_k, \boldsymbol{Y}_{k+1}, \ldots, \boldsymbol{Y}_s)}{P(A_{k,t-1} \mid \mathcal{R}_{0,k-1}, A_{k,s}, \boldsymbol{B}_k, \boldsymbol{Y}_{k+1}, \ldots, \boldsymbol{Y}_{s-1})} \qquad (40)
\end{aligned}
$$

and

$$
\begin{aligned}
&P(A_{k,t-1} \mid \mathcal{R}_{0,k-1}, A_{k,s}, \boldsymbol{B}_k, \boldsymbol{Y}_{k+1}, \ldots, \boldsymbol{Y}_s) \\
&= \prod_{l=s+1}^{t-1} P(R_{0,l} = 0 \mid \mathcal{R}_{0,k-1}, A_{k,l-1}, \boldsymbol{B}_k, \boldsymbol{Y}_{k+1}, \ldots, \boldsymbol{Y}_s) \qquad (41) \\
&= \prod_{l=s+1}^{t-1} P(R_{0,l} = 0 \mid \mathcal{R}_{0,k-1}, A_{k,l-1}, \boldsymbol{B}_k) \\
&= P(A_{k,t-1} \mid \mathcal{R}_{0,k-1}, A_{k,s}, \boldsymbol{B}_k) \qquad (42)
\end{aligned}
$$

So, from equations (40) and (42), we have that for $k < s \leq t$,

$$
f(\boldsymbol{Y}_s \mid \mathcal{R}_{0,k-1}, A_{k,t-1}, \boldsymbol{B}_k, \boldsymbol{Y}_{k+1}, \ldots, \boldsymbol{Y}_{s-1}) = f(\boldsymbol{Y}_s \mid \mathcal{R}_{0,k-1}, A_{k,s}, \boldsymbol{B}_k, \boldsymbol{Y}_{k+1}, \ldots, \boldsymbol{Y}_{s-1}) \qquad (43)
$$

It follows from equations (1) and (9) that

$$
f(\boldsymbol{Y}_s \mid \mathcal{R}_{0,k-1}, A_{k,s}, \boldsymbol{B}_k, \boldsymbol{Y}_{k+1}, \ldots, \boldsymbol{Y}_{s-1}) = f(\boldsymbol{Y}_s \mid \boldsymbol{X}, \boldsymbol{Y}_{s-1}) \qquad (44)
$$

So, using equations (39), (43) and (44), we have

$$
\begin{aligned}
f(\boldsymbol{Y}_{k+1}, \ldots, \boldsymbol{Y}_t \mid \mathcal{R}_{0,k-1}, A_{k,t-1}, \boldsymbol{B}_k) &= \prod_{s=k+1}^{t} f(\boldsymbol{Y}_s \mid \boldsymbol{X}, \boldsymbol{Y}_{s-1}) \\
&= f(\boldsymbol{Y}_{k+1}, \ldots, \boldsymbol{Y}_t \mid \boldsymbol{X}, \boldsymbol{Y}_k)
\end{aligned}
$$

So, line (38) follows.

The following example shows that independent return does not imply strong independent return. However, it does not show that this matters for inference.

Suppose $T = 5$, $m = 1$ and there is no baseline covariate $\boldsymbol{X}$ and no measurement error. Suppose that Y_1, Y_2, Y_3 and Y_4 are independent and $Y_5 = Y_4$, with $Y_1 \sim$ Bernoulli(0.5), and $Y_2, Y_3 \sim$ Normal$(0,1)$, and $Y_4 \sim$ Bernoulli(0.1). Suppose that $P(R_2) = 1$, $P(R_3 = 0 \mid Y_1, Y_2, Y_3, Y_4) = 0.99$, $P(R_4 = R_5 = 1 \mid R_3 = 1) = 1$, $P(R_4 = 0 \mid R_3 = 0) = 1$, and

$$
P(R_5 = 1 \mid R_4 = 0, Y_1, Y_2, Y_3, Y_4) = \begin{cases} 1 & \text{if } Y_1 = Y_4 \\ 0 & \text{if } Y_1 \neq Y_4 \end{cases}
$$

Independent return holds, because $P(R_5 = 1 \mid R_4 = 0, Y_2, Y_3, Y_4) = P(R_5 = 1 \mid R_4 = 0, Y_2) = 0.5$. However, strong independent return does not hold, because $P(R_5 = 1 \mid R_4 = 0, Y_1, Y_2, Y_3, Y_4) \neq P(R_5 = 1 \mid R_4 = 0, Y_1, Y_2)$.

Consider what will happen when aMVN imputation and uMVN imputation are used. Autoregressive MVN 'knows' that $Y_1 \perp\!\!\!\perp Y_5$ and (asymptotically) imputes missing Y_5 as 0.1, which is correct. Unstructured MVN 'looks' at the observed data and (asymptotically) 'sees' that the correlation between Y_1 and Y_5 in individuals in whom Y_5 is observed equals 1, but that the correlation between Y_5 and Y_2 is zero. So (asymptotically), using formula (18), missing Y_5 values will be imputed as $0.1 + 1 \times \sqrt{\frac{0.1\times 0.9}{0.5\times 0.5}}(Y_1 - 0.5)$. This is 0.4 if $Y_1 = 1$ and -0.2 if $Y_1 = 0$. So, the average imputed value of Y_5 is $(0.4 - 0.2)/2 = 0.1$, which is correct.

Note that dDTIC does hold in this example.

1.4 Proof of Theorem 4

Let $\boldsymbol{G}_t(\boldsymbol{r}_t)$ denote what would have been an individual's value of $\boldsymbol{G}_t$ if their value of $\mathcal{R}_{0,t}$ had been $\boldsymbol{r}_t$ and the history $\mathcal{F}_t$ of their covariates and underlying outcomes were unchanged. Note that $\boldsymbol{G}_t = \boldsymbol{G}_t(\mathcal{R}_{0,t})$. As an example, suppose that $\mathcal{R}_{0,i4} = (1,1,0,1)^\top$. Then $\boldsymbol{G}_4 = \boldsymbol{G}_4(\mathcal{R}_{04}) = (\boldsymbol{Y}_{i1}^\top, \boldsymbol{Y}_{i2}^\top, \boldsymbol{Y}_{i4}^\top, \boldsymbol{X}_i^\top)^\top$, whereas $\boldsymbol{G}_4(\boldsymbol{r}_4) = (\boldsymbol{Y}_{i1}^\top, \boldsymbol{Y}_{i3}^\top, \boldsymbol{X}_i^\top)^\top$ for $\boldsymbol{r}_4 = (1,0,1,0)^\top$ and $\boldsymbol{G}_4(\boldsymbol{r}_4) = (\boldsymbol{Y}_{i1}^\top, \boldsymbol{X}_i^\top)^\top$ for $\boldsymbol{r}_4 = (1,0,0,0)^\top$.

Consider the reparameterisation of the unstructured MVN model given by equations (83)–(85). Let $\boldsymbol{\theta}$ denote the parameters in this model, i.e. $\boldsymbol{\theta} = (\boldsymbol{\mu}_1, \boldsymbol{\mu}_{T+1}, \boldsymbol{\Sigma}_{1,1}, \boldsymbol{\Sigma}_{1,T+1}, \boldsymbol{\Sigma}_{T+1,T+1},\ \boldsymbol{\alpha}_2, \boldsymbol{\beta}_2,\ \gamma_2, \boldsymbol{\sigma}_2, \ldots,\ \boldsymbol{\alpha}_T, \boldsymbol{\beta}_T, \boldsymbol{\delta}_{T,1},\ \ldots, \boldsymbol{\delta}_{T,T-2}, \gamma_T, \boldsymbol{\sigma}_T)^\top$. So, $\boldsymbol{\mu} = \boldsymbol{\mu}(\boldsymbol{\theta})$ and $\boldsymbol{\Sigma} = \boldsymbol{\Sigma}(\boldsymbol{\theta})$ are deterministic functions of $\boldsymbol{\theta}$ (e.g. equations (86)–(88) give the functions in the special case where $\boldsymbol{\delta}_{tj} = \boldsymbol{0}$). For $t \geq 2$, any value $\boldsymbol{r}_{t-1}$ of $\mathcal{R}_{0,t-1}$ and any value $\boldsymbol{g}_{t-1}$ of $\boldsymbol{G}_{t-1}(\boldsymbol{r}_{t-1})$, let $\boldsymbol{h}_t(\boldsymbol{y}_t \mid \boldsymbol{r}_{t-1}, \boldsymbol{g}_{t-1};\ \boldsymbol{\theta})$ denote the derivative with respect to $\boldsymbol{\theta}$ of the log conditional density function of $\boldsymbol{Y}_t$ given

$\boldsymbol{G}_{t-1}(\boldsymbol{r}_{t-1}) = \boldsymbol{g}_{t-1}$ evaluated at $\boldsymbol{Y}_t = \boldsymbol{y}_t$ when $(\boldsymbol{Y}_1, \ldots, \boldsymbol{Y}_T, \boldsymbol{X})$ is assumed to be distributed Normal$(\boldsymbol{\mu}(\boldsymbol{\theta}), \boldsymbol{\Sigma}(\boldsymbol{\theta}))$. Similarly, let $\boldsymbol{h}_1(\boldsymbol{x}, \boldsymbol{y}_1; \boldsymbol{\theta})$ denote the derivative of the log density of $(\boldsymbol{X}, \boldsymbol{Y}_1)$ when $(\boldsymbol{Y}_1, \ldots, \boldsymbol{Y}_T, \boldsymbol{X}) \sim$ Normal$(\boldsymbol{\mu}(\boldsymbol{\theta}), \boldsymbol{\Sigma}(\boldsymbol{\theta}))$.

The MLE of $\boldsymbol{\theta}$ obtained by fitting the MVN model to the observed data and ignoring the missingness mechanism is the value of $\boldsymbol{\theta}$ for which the derivative with respect to $\boldsymbol{\theta}$ of the log likelihood function $\sum_{i=1}^N L_i(\boldsymbol{\theta})$ equals zero, where

$$\frac{\partial L(\boldsymbol{\theta})}{\partial \boldsymbol{\theta}} = \boldsymbol{h}_1(\boldsymbol{x}, \boldsymbol{y}_1; \boldsymbol{\theta}) + \sum_{t=2}^{\top} \sum_{\boldsymbol{r}_{t-1}} \left\{ \begin{array}{c} I(R_{0,t} = R_{0,t-1} = 1, \mathcal{R}_{0,t-1} = \boldsymbol{r}_{t-1}) \\ \times \boldsymbol{h}_t(\boldsymbol{y}_t \mid \boldsymbol{r}_{t-1}, \boldsymbol{G}_{t-1}(\boldsymbol{r}_{t-1});\ \boldsymbol{\theta}) \end{array} \right\}$$
$$+ \sum_{t=3}^{\top} \sum_{s=1}^{t-2} \sum_{\boldsymbol{r}_s} \left\{ \begin{array}{l} I(R_{0,t} = 1, R_{0,t-1} = \ldots = R_{0,s+1} = 0, R_{0,s} = 1, \mathcal{R}_{0,s} = \boldsymbol{r}_s) \\ \quad \times \boldsymbol{h}_t(\boldsymbol{y}_t \mid \boldsymbol{r}_s, \boldsymbol{G}_s(\boldsymbol{r}_s);\ \boldsymbol{\theta}) \end{array} \right\} \quad (45)$$

and $\sum_{\boldsymbol{r}_k}$ means the sum over all possible k-vectors whose elements are zero or one.

If $(\boldsymbol{Y}_1, \boldsymbol{X}, \boldsymbol{\epsilon}_2, \ldots, \boldsymbol{\epsilon}_T)$ is normally distributed, then $(\boldsymbol{Y}_1, \ldots, \boldsymbol{Y}_T, \boldsymbol{X})$ is normally distributed with mean $\boldsymbol{\mu}$ and $\boldsymbol{\Sigma}$, and so at the true value of $\boldsymbol{\theta}$ (Stefanski and Boos, 2000).

$$E\{\boldsymbol{h}_t(\boldsymbol{Y}_t \mid \boldsymbol{r}_s, \boldsymbol{G}_s(\boldsymbol{r}_s);\ \boldsymbol{\theta}) \mid \boldsymbol{G}_s(\boldsymbol{r}_s)\} = \boldsymbol{0} \qquad \forall s, t, \boldsymbol{r}_s \text{ such that } 1 \leq s < t \leq T. \quad (46)$$

Even if $(\boldsymbol{Y}_1, \boldsymbol{X}, \boldsymbol{\epsilon}_2, \ldots, \boldsymbol{\epsilon}_T)$ is not normally distributed, equation (46) still holds, provided that $\boldsymbol{\epsilon}_t$ has mean zero and its variance does not depend on $\mathcal{F}_{t-1}$. This is because, treated as a function of $\boldsymbol{y}_{it}$ $(i = 1, \ldots, N)$, $\sum_{i=1}^N \boldsymbol{h}_t(\boldsymbol{y}_{it} \mid \boldsymbol{r}_s, \boldsymbol{G}_{is}(\boldsymbol{r}_s);\ \boldsymbol{\theta})$ depends only on (and is a linear combination of) $\sum_{i=1}^N \boldsymbol{y}_{it}$, $\sum_{i=1}^N \boldsymbol{y}_{it}\boldsymbol{y}_{it}^\top$ and $\sum_{i=1}^N \boldsymbol{y}_{it}\boldsymbol{G}_{is}(\boldsymbol{r}_s)^\top$. Therefore, $E\{\boldsymbol{h}_t(\boldsymbol{Y}_t \mid \boldsymbol{r}_s, \boldsymbol{G}_s(\boldsymbol{r}_s);\ \boldsymbol{\theta}) \mid \boldsymbol{G}_s(\boldsymbol{r}_s)\}$ is a linear combination of $E(\boldsymbol{Y}_t \mid \boldsymbol{G}_s(\boldsymbol{r}_s))$ and $\text{Var}(\boldsymbol{Y}_t \mid \boldsymbol{G}_s(\boldsymbol{r}_s))$, and hence only depends on the distribution of $(\boldsymbol{X}^\top, \boldsymbol{Y}_1)^\top$ and $\boldsymbol{\epsilon}_2, \ldots, \boldsymbol{\epsilon}_t$ through their means and variances.

Now, for any $\boldsymbol{r}_{t-1}$ with final element equal to one,

$$
\begin{aligned}
&E\{I(R_{0,t} = R_{0,t-1} = 1, \mathcal{R}_{0,t-1} = \boldsymbol{r}_{t-1})\ \boldsymbol{h}_t(\boldsymbol{Y}_t \mid \boldsymbol{r}_{t-1}, \boldsymbol{G}_{t-1}(\boldsymbol{r}_{t-1});\ \boldsymbol{\theta})\} \\
&= P(R_{0,t} = R_{0,t-1} = 1, \mathcal{R}_{0,t-1} = \boldsymbol{r}_{t-1}) \\
&\quad \times E\{\boldsymbol{h}_t(\boldsymbol{Y}_t \mid \boldsymbol{r}_{t-1}, \boldsymbol{G}_{t-1}(\boldsymbol{r}_{t-1});\ \boldsymbol{\theta}) \mid R_{0,t} = R_{0,t-1} = 1, \mathcal{R}_{0,t-1} = \boldsymbol{r}_{t-1}\} \\
&= P(R_{0,t} = R_{0,t-1} = 1, \mathcal{R}_{0,t-1} = \boldsymbol{r}_{t-1}) \\
&\quad \times E[E\{\boldsymbol{h}_t(\boldsymbol{Y}_t \mid \boldsymbol{r}_{t-1}, \boldsymbol{G}_{t-1}(\boldsymbol{r}_{t-1});\ \boldsymbol{\theta}) \mid \boldsymbol{G}_{t-1}(\boldsymbol{r}_{t-1}), R_{0,t} = R_{0,t-1} = 1, \mathcal{R}_{0,t-1} = \boldsymbol{r}_{t-1}\} \\
&\qquad\qquad \mid R_{0,t} = R_{0,t-1} = 1, \mathcal{R}_{0,t-1} = \boldsymbol{r}_{t-1}] \qquad (47)
\end{aligned}
$$

Using equation (13), equation (47) reduces to

$$
\begin{aligned}
&P(R_{0,t} = R_{0,t-1} = 1, \mathcal{R}_{0,t-1} = \boldsymbol{r}_{t-1}) \\
&\quad \times E[E\{\boldsymbol{h}_t(\boldsymbol{Y}_t \mid \boldsymbol{r}_{t-1}, \boldsymbol{G}_{t-1}(\boldsymbol{r}_{t-1});\ \boldsymbol{\theta}) \mid \boldsymbol{G}_{t-1}(\boldsymbol{r}_{t-1})\} \mid R_{0,t} = R_{0,t-1} = 1, \mathcal{R}_{0,t-1} = \boldsymbol{r}_{t-1}]
\end{aligned}
$$

which, by equation (46), equals zero at the true value of $\boldsymbol{\theta}$, because, as stated above, $E\{\boldsymbol{h}_t(\boldsymbol{Y}_t \mid \boldsymbol{r}_{t-1}, \boldsymbol{G}_{t-1}(\boldsymbol{r}_{t-1});\ \boldsymbol{\theta}) \mid \boldsymbol{G}_{t-1}(\boldsymbol{r}_{t-1})\} = \boldsymbol{0}$ at the true value of $\boldsymbol{\theta}$.

Similarly, using equation (14), it follows that

$$
I(R_{0,t} = 1, R_{0,t-1} = \ldots = R_{0,s+1} = 0, R_{0,s} = 1, \mathcal{R}_{0,s} = \boldsymbol{r}_s)\boldsymbol{h}_t(\boldsymbol{Y}_t \mid \boldsymbol{r}_s, \boldsymbol{G}_s(\boldsymbol{r}_s);\ \boldsymbol{\theta})
$$

also has expectation zero at the true value of $\boldsymbol{\theta}$.

Therefore equation (45) has expectation zero at the true value of $\boldsymbol{\theta}$. So, under standard regularity assumptions, the MLE of $\boldsymbol{\theta}$ from the unstructured MVN model is consistent (Stefanski and Boos, 2000).

The proof that autoregressive MVN yields consistent estimators when independent return holds is analogous. The parameters $\boldsymbol{\delta}_{tj}$ are removed from $\boldsymbol{\theta}$, since they are constrained to equal zero. Now $\boldsymbol{h}_t(\boldsymbol{Y}_t \mid \boldsymbol{r}_s, \boldsymbol{G}_s(\boldsymbol{r}_s);\ \boldsymbol{\theta})$ can be written as $\boldsymbol{h}_t(\boldsymbol{Y}_t \mid \boldsymbol{r}_s, \boldsymbol{Y}_s;\ \boldsymbol{\theta})$ whenever the final element of $\boldsymbol{r}_s$ equals one. The proof for unstructured MVN continues to apply for autoregressive MVN, once $\boldsymbol{h}_t(\boldsymbol{Y}_t \mid \boldsymbol{r}_s, \boldsymbol{G}_s(\boldsymbol{r}_s);\ \boldsymbol{\theta})$ have been replaced by $\boldsymbol{h}_t(\boldsymbol{Y}_t \mid \boldsymbol{r}_s, \boldsymbol{Y}_s;\ \boldsymbol{\theta})$ and equations (13) and (14) have been replaced

by versions of those equations with $(X, \boldsymbol{Y}_{t-1})$ and $\boldsymbol{X}, \boldsymbol{Y}_k$ in place of $\boldsymbol{G}_{t-1}$ and $\boldsymbol{G}_k$, as Theorem 5 allows.

1.5 Proof of Theorem 5

By equation (1) and dDTIC, we have

$$f(\boldsymbol{Y}_t \mid \mathcal{F}_{t-1}, \mathcal{R}_{0,t-2}, R_{0,t-1} = R_{0,t} = 1) \;=\; f(\boldsymbol{Y}_t \mid \mathcal{F}_{t-1}) = f(\boldsymbol{Y}_t \mid \boldsymbol{X}, \boldsymbol{Y}_{t-1})$$

which implies that equation (13) holds.

Next we prove that equation (14) holds.

$$\begin{aligned}
& f(\boldsymbol{Y}_t \mid \boldsymbol{G}_s, \mathcal{R}_{0,s-1}, A_{s,t-1}, R_{0,t} = 1) \\
&= f(\boldsymbol{Y}_t \mid \boldsymbol{G}_s, \mathcal{R}_{0,s-1}, A_{s,t-1}) \\
&\qquad \times \frac{P(R_{0,t} = 1 \mid \boldsymbol{G}_s, \boldsymbol{Y}_t, \mathcal{R}_{0,s-1}, A_{s,t-1})}{P(R_{0,t} = 1 \mid \boldsymbol{G}_s, \mathcal{R}_{0,s-1}, A_{s,t-1})} \\
&= f(\boldsymbol{Y}_t \mid \boldsymbol{G}_s, \mathcal{R}_{0,s-1}, A_{s,t-1}) && (48) \\
&= \int f(\boldsymbol{Y}_t \mid \boldsymbol{G}_s, \boldsymbol{Y}_{t-1}, \mathcal{R}_{0,s-1}, A_{s,t-1}) f(\boldsymbol{Y}_{t-1} \mid \boldsymbol{G}_s, \mathcal{R}_{0,s-1}, A_{s,t-1})\, d\boldsymbol{Y}_{t-1} \\
&= \int f(\boldsymbol{Y}_t \mid \boldsymbol{X}, \boldsymbol{Y}_{t-1}) f(\boldsymbol{Y}_{t-1} \mid \boldsymbol{G}_s, \mathcal{R}_{0,s-1}, A_{s,t-1})\, d\boldsymbol{Y}_{t-1} && (49) \\
&= \int f(\boldsymbol{Y}_t \mid \boldsymbol{X}, \boldsymbol{Y}_{t-1})\, f(\boldsymbol{Y}_{t-1} \mid \boldsymbol{G}_s, \mathcal{R}_{0,s-1}, A_{s,t-2}) \\
&\qquad \times \frac{P(R_{0,t-1} = 0 \mid \boldsymbol{G}_s, \boldsymbol{Y}_{t-1}, \mathcal{R}_{0,s-1}, A_{s,t-2})}{P(R_{0,t-1} = 0 \mid \boldsymbol{G}_s, \mathcal{R}_{0,s-1}, A_{s,t-2})}\, d\boldsymbol{Y}_{t-1} \\
&= \int f(\boldsymbol{Y}_t \mid \boldsymbol{X}, \boldsymbol{Y}_{t-1})\, f(\boldsymbol{Y}_{t-1} \mid \boldsymbol{G}_s, \mathcal{R}_{0,s-1}, A_{s,t-2})\, d\boldsymbol{Y}_{t-1} && (50) \\
&= \int\int f(\boldsymbol{Y}_t \mid \boldsymbol{X}, \boldsymbol{Y}_{t-1})\, f(\boldsymbol{Y}_{t-1} \mid \boldsymbol{X}, \boldsymbol{Y}_{t-2})\, f(\boldsymbol{Y}_{t-2} \mid \boldsymbol{G}_s, \mathcal{R}_{0,s-1}, A_{s,t-3})\, d\boldsymbol{Y}_{t-2}\, d\boldsymbol{Y}_{t-1} \\
&= \int \dots \int f(\boldsymbol{Y}_t \mid \boldsymbol{X}, \boldsymbol{Y}_{t-1})\, f(\boldsymbol{Y}_{t-1} \mid \boldsymbol{X}, \boldsymbol{Y}_{t-2})\, \dots f(\boldsymbol{Y}_{s+2} \mid \boldsymbol{X}, \boldsymbol{Y}_{s+1}) \\
&\qquad \times f(\boldsymbol{Y}_{s+1} \mid \boldsymbol{G}_s, \mathcal{R}_{0,s-1}, R_{0,s} = 1)\, d\boldsymbol{Y}_{s+1}\, d\boldsymbol{Y}_{s+2} \dots\, d\boldsymbol{Y}_{t-2}\, d\boldsymbol{Y}_{t-1} \\
&= \int \dots \int f(\boldsymbol{Y}_t \mid \boldsymbol{X}, \boldsymbol{Y}_{t-1})\, f(\boldsymbol{Y}_{t-1} \mid \boldsymbol{X}, \boldsymbol{Y}_{t-2})\, \dots f(\boldsymbol{Y}_{s+2} \mid \boldsymbol{X}, \boldsymbol{Y}_{s+1}) \\
&\qquad \times f(\boldsymbol{Y}_{s+1} \mid \boldsymbol{X}, \boldsymbol{Y}_s)\, d\boldsymbol{Y}_{s+1}\, d\boldsymbol{Y}_{s+2} \dots\, d\boldsymbol{Y}_{t-2}\, d\boldsymbol{Y}_{t-1} \\
&= f(\boldsymbol{Y}_t \mid \boldsymbol{X}, \boldsymbol{Y}_s) && (51)
\end{aligned}$$

which implies that equation (14) holds. Lines (48) and (50) follow from strong

independent return. Line (49) follows from the same argument used above to prove equation (13).

The proofs are analogous when independent return, rather than strong independent return, holds.

1.6 Proof of Theorem 6

The complete-data least-squares estimators of $\boldsymbol{\beta}_t$ and $\boldsymbol{\gamma}_t$ are

$$\begin{bmatrix} \hat{\boldsymbol{\beta}}_{cd,t} \\ \hat{\boldsymbol{\gamma}}_{cd,t} \end{bmatrix} = \begin{bmatrix} \boldsymbol{c}_{t-1,t-1} & \boldsymbol{c}_{t-1,T+1} \\ \boldsymbol{c}_{T+1,t-1} & \boldsymbol{c}_{T+1,T+1} \end{bmatrix}^{-1} \begin{bmatrix} \boldsymbol{c}_{t-1,t} \\ \boldsymbol{c}_{T+1,t} \end{bmatrix} \tag{52}$$

Solving these simultaneous equations yields

$$\begin{aligned} \hat{\boldsymbol{\beta}}_{cd,t} &= (\boldsymbol{c}_{t-1,t-1} - \boldsymbol{c}_{t-1,T+1}\boldsymbol{c}_{T+1,T+1}^{-1}\boldsymbol{c}_{T+1,t-1})^{-1} \\ &\quad (\boldsymbol{c}_{t-1,t} - \boldsymbol{c}_{t-1,T+1}\boldsymbol{c}_{T+1,T+1}^{-1}\boldsymbol{c}_{T+1,t}) && (53) \\ \hat{\boldsymbol{\gamma}}_{cd,t} &= \boldsymbol{c}_{T+1,T+1}^{-1}\boldsymbol{c}_{T+1,t} - \boldsymbol{c}_{T+1,T+1}^{-1}\boldsymbol{c}_{T+1,t-1}\hat{\boldsymbol{\beta}}_{cd,t} && (54) \end{aligned}$$

The least-squares estimator of $\boldsymbol{\alpha}_t$ is

$$\hat{\boldsymbol{\alpha}}_{cd,t} = \bar{\boldsymbol{Y}}_t - \hat{\boldsymbol{\beta}}_{cd,t}^{\top}\bar{\boldsymbol{Y}}_{t-1} - \hat{\boldsymbol{\gamma}}_{cd,t}^{\top}\bar{\boldsymbol{X}}. \tag{55}$$

If $\boldsymbol{\beta}_t$ is constrained to equal $\boldsymbol{I}$, then equations (54) and (55) still hold.

1.7 Proof of Theorem 7

When data are monotone missing and the $\boldsymbol{\delta}_{tj}$'s are constrained to equal zero, equation (45) reduces to

$$\partial L(\boldsymbol{\theta})/\partial\boldsymbol{\theta} = \sum_{t=1}^{\top} I(R_{0,t} = 1)\boldsymbol{h}(\boldsymbol{Y}_t \mid \mathbf{1}_{t-1}, \mathcal{F}_{t-1};\ \boldsymbol{\theta})$$

where $\mathbf{1}_{t-1}$ denotes a $(t-1)$-vector of ones. The maximum likelihood estimate of $\boldsymbol{\theta}$ can be obtained by fitting the models defined by equations (84) and (85) with the $\boldsymbol{\delta}_{tj}$'s omitted and estimating $\boldsymbol{\mu}_1$, $\boldsymbol{\mu}_{T+1}$, $\boldsymbol{\Sigma}_{1,1}$, $\boldsymbol{\Sigma}_{1,T+1}$ and $\boldsymbol{\Sigma}_{T+1,T+1}$ by the corresponding sample means, variances and covariances. Fitting by maximum

likelihood the models given by equations (84) and (85) with the $\boldsymbol{\delta}_{tj}$'s omitted is equivalent to fitting them by least squares, which is the method proposed by A&G.

When data are monotone missing, the imputed value of $\boldsymbol{Y}_t$ obtained using autoregressive MVN, $E(\boldsymbol{Y}_t \mid \boldsymbol{G}_T)$, can be written as $E(\boldsymbol{Y}_t \mid \boldsymbol{G}_T) = E(\boldsymbol{Y}_t \mid \boldsymbol{G}_{t-1}) = E\{E(\boldsymbol{Y}_t \mid \boldsymbol{Y}_{t-1}) \mid \boldsymbol{G}_{t-1}\}$. Therefore, aMVN imputation is equivalent to the iterative imputation procedure that is LI imputation.

1.8 Proof of Theorem 8

We begin by proving b). So, assume that mortal-cohort independent return and independent death hold. Then

$$\begin{aligned} &P(D \geq t \mid \mathcal{R}_{0,k-1}, A_{k,t-1}, \boldsymbol{X}, \boldsymbol{Y}_k, \ldots, \boldsymbol{Y}_t) \\ &= P(D \geq t \mid \mathcal{R}_{0,k-1}, A_{k,t-1}, \boldsymbol{X}, \boldsymbol{Y}_k) \\ &\quad \times \frac{f(\boldsymbol{Y}_{k+1}, \ldots, \boldsymbol{Y}_t \mid \mathcal{R}_{0,k-1}, A_{k,t-1}, \boldsymbol{X}, \boldsymbol{Y}_k, D \geq t)}{f(\boldsymbol{Y}_{k+1}, \ldots, \boldsymbol{Y}_t \mid \mathcal{R}_{0,k-1}, A_{k,t-1}, \boldsymbol{X}, \boldsymbol{Y}_k)} \end{aligned} \tag{56}$$

Equations (20) and (26) imply that

$$f(\boldsymbol{Y}_t \mid \mathcal{R}_{0,k-1}, A_{k,t-1}, \boldsymbol{X}, \boldsymbol{Y}_k, \ldots, \boldsymbol{Y}_{t-1}, D \geq t) = f(\boldsymbol{Y}_t \mid \boldsymbol{X}, \boldsymbol{Y}_{t-1}) \tag{57}$$

Now, for $k+1 \leq s < t$,

$$f(\boldsymbol{Y}_s \mid \mathcal{R}_{0,k-1}, A_{k,t-1}, \boldsymbol{X}, \boldsymbol{Y}_k, \ldots, \boldsymbol{Y}_{s-1}, D \geq t) \tag{58}$$

$$\begin{aligned} &= f(\boldsymbol{Y}_s \mid \mathcal{R}_{0,k-1}, A_{k,t-1}, \boldsymbol{X}, \boldsymbol{Y}_k, \ldots, \boldsymbol{Y}_{s-1}, D \geq t-1) \\ &\quad \times \frac{P(D \geq t \mid \mathcal{R}_{0,k-1}, A_{k,t-1}, \boldsymbol{X}, \boldsymbol{Y}_k, \ldots, \boldsymbol{Y}_s, D \geq t-1)}{P(D \geq t \mid \mathcal{R}_{0,k-1}, A_{k,t-1}, \boldsymbol{X}, \boldsymbol{Y}_k, \ldots, \boldsymbol{Y}_{s-1}, D \geq t-1)} \end{aligned} \tag{59}$$

$$= f(\boldsymbol{Y}_s \mid \mathcal{R}_{0,k-1}, A_{k,t-1}, \boldsymbol{X}, \boldsymbol{Y}_k, \ldots, \boldsymbol{Y}_{s-1}, D \geq t-1) \tag{60}$$

$$\begin{aligned} &= f(\boldsymbol{Y}_s \mid \mathcal{R}_{0,k-1}, A_{k,t-2}, \boldsymbol{X}, \boldsymbol{Y}_k, \ldots, \boldsymbol{Y}_{s-1}, D \geq t-1) \\ &\quad \times \frac{P(R_{0,t-1} = 0 \mid \mathcal{R}_{0,k-1}, A_{k,t-2}, \boldsymbol{X}, \boldsymbol{Y}_k, \ldots, \boldsymbol{Y}_s, D \geq t-1)}{P(R_{0,t-1} = 0 \mid \mathcal{R}_{0,k-1}, A_{k,t-2}, \boldsymbol{X}, \boldsymbol{Y}_k, \ldots, \boldsymbol{Y}_{s-1}, D \geq t-1)} \end{aligned} \tag{61}$$

$$= f(\boldsymbol{Y}_s \mid \mathcal{R}_{0,k-1}, A_{k,t-2}, \boldsymbol{X}, \boldsymbol{Y}_k, \ldots, \boldsymbol{Y}_{s-1}, D \geq t-1) \tag{62}$$

$$= f(\boldsymbol{Y}_s \mid \mathcal{R}_{0,k-1}, A_{k,s-1}, \boldsymbol{X}, \boldsymbol{Y}_k, \ldots, \boldsymbol{Y}_{s-1}, D \geq s) \tag{63}$$

$$= f(\boldsymbol{Y}_s \mid \boldsymbol{X}, \boldsymbol{Y}_{s-1}) \tag{64}$$

Line (60) follows because of independent death. Line (62) follows because of mortal-cohort independent return. Line (63) follows by induction. Line (64) follows by equation (57). Hence, from equation (64),

$$
\begin{aligned}
& f(\boldsymbol{Y}_{k+1}, \ldots, \boldsymbol{Y}_t \mid \mathcal{R}_{0,k-1}, A_{k,t-1}, \boldsymbol{X}, \boldsymbol{Y}_k, D \geq t) \\
& = \prod_{s=k+1}^{t} f(\boldsymbol{Y}_s \mid \mathcal{R}_{0,k-1}, A_{k,t-1}, \boldsymbol{X}, \boldsymbol{Y}_k, \ldots, \boldsymbol{Y}_{s-1}, D \geq t) \\
& = \prod_{s=k+1}^{t} f(\boldsymbol{Y}_s \mid \boldsymbol{X}, \boldsymbol{Y}_{s-1}) \\
& = f(\boldsymbol{Y}_{k+1}, \ldots, \boldsymbol{Y}_t \mid \boldsymbol{X}, \boldsymbol{Y}_k) \qquad (65)
\end{aligned}
$$

It follows from equations (56) and (65) that

$$
P(D \geq t \mid \mathcal{R}_{0,k-1}, A_{k,t-1}, \boldsymbol{X}, \boldsymbol{Y}_k, \ldots, \boldsymbol{Y}_t) = P(D \geq t \mid \mathcal{R}_{0,k-1}, A_{k,t-1}, \boldsymbol{X}, \boldsymbol{Y}_k) \quad (66)
$$

Finally,

$$
\begin{aligned}
& P(R_{0,t} = 1 \mid \mathcal{R}_{0,k-1}, A_{k,t-1}, \boldsymbol{X}, \boldsymbol{Y}_k, \ldots \boldsymbol{Y}_t) \qquad (67) \\
& = P(R_{0,t} = 1 \mid \mathcal{R}_{0,k-1}, A_{k,t-1}, \boldsymbol{X}, \boldsymbol{Y}_k, \ldots \boldsymbol{Y}_t, D \geq t) \\
& \qquad \times P(D \geq t \mid \mathcal{R}_{0,k-1}, A_{k,t-1}, \boldsymbol{X}, \boldsymbol{Y}_k, \ldots \boldsymbol{Y}_t) \qquad (68) \\
& = P(R_{0,t} = 1 \mid \mathcal{R}_{0,k-1}, A_{k,t-1}, \boldsymbol{X}, \boldsymbol{Y}_k, D \geq t) \\
& \qquad \times P(D \geq t \mid \mathcal{R}_{0,k-1}, A_{k,t-1}, \boldsymbol{X}, \boldsymbol{Y}_k) \qquad (69) \\
& = P(R_{0,t} = 1 \mid \mathcal{R}_{0,k-1}, A_{k,t-1}, \boldsymbol{X}, \boldsymbol{Y}_k) \qquad (70)
\end{aligned}
$$

Hence, independent return holds in the supplemented process. Line (69) follows from mortal-cohort independent return and equation (66).

The proof of c) is analogous to that of b). The changes are as follows. Replace $\boldsymbol{X}$ by $\boldsymbol{G}_{k-1}$ in equations (56), (58)–(63) and (66)–(70). Replace equation (57) by

$$
f(\boldsymbol{Y}_t \mid \mathcal{R}_{0,k-1}, A_{k,t-1}, \boldsymbol{G}_{k-1}, \boldsymbol{Y}_k, \ldots, \boldsymbol{Y}_{t-1}, D \geq t) = f(\boldsymbol{Y}_t \mid \boldsymbol{X}, \boldsymbol{Y}_{t-1})
$$

and replace equation (65) by

$$
f(\boldsymbol{Y}_{k+1}, \ldots, \boldsymbol{Y}_t \mid \mathcal{R}_{0,k-1}, A_{k,t-1}, \boldsymbol{G}_{k-1}, \boldsymbol{Y}_k, D \geq t) = f(\boldsymbol{Y}_{k+1}, \ldots, \boldsymbol{Y}_t \mid \boldsymbol{X}, \boldsymbol{Y}_k). \quad (71)
$$

Finally, we prove a).

$$
\begin{aligned}
&P(R_{0,t} = 1 \mid \mathcal{R}_{0,k-1}, A_{k,t-1}, \boldsymbol{X}, \boldsymbol{Y}_k, \boldsymbol{Y}_{k+1}, \ldots, \boldsymbol{Y}_t, D \geq t) \\
&= \int P(R_{0,t} = 1 \mid \mathcal{R}_{0,k-1}, A_{k,t-1}, \boldsymbol{G}_{k-1}, \boldsymbol{Y}_k, \boldsymbol{Y}_{k+1}, \ldots, \boldsymbol{Y}_t, D \geq t) \\
&\qquad \times f(\boldsymbol{G}_{k-1} \mid \mathcal{R}_{0,k-1}, A_{k,t-1}, \boldsymbol{X}, \boldsymbol{Y}_k, \boldsymbol{Y}_{k+1}, \ldots \boldsymbol{Y}_t, D \geq t)\, d\boldsymbol{G}_{k-1} \\
&= \int P(R_{0,t} = 1 \mid \mathcal{R}_{0,k-1}, A_{k,t-1}, \boldsymbol{G}_{k-1}, \boldsymbol{Y}_k, D \geq t) \\
&\qquad \times f(\boldsymbol{G}_{k-1} \mid \mathcal{R}_{0,k-1}, A_{k,t-1}, \boldsymbol{X}, \boldsymbol{Y}_k, \boldsymbol{Y}_{k+1}, \ldots \boldsymbol{Y}_t, D \geq t)\, d\boldsymbol{G}_{k-1} \qquad (72)
\end{aligned}
$$

Line (72) follows by mortal-cohort strong independent return. Now,

$$
\begin{aligned}
&f(\boldsymbol{G}_{k-1} \mid \mathcal{R}_{0,k-1}, A_{k,t-1}, \boldsymbol{X}, \boldsymbol{Y}_k, \boldsymbol{Y}_{k+1}, \ldots \boldsymbol{Y}_t, D \geq t) \\
&= f(\boldsymbol{G}_{k-1} \mid \mathcal{R}_{0,k-1}, A_{k,t-1}, \boldsymbol{X}, \boldsymbol{Y}_k, D \geq t) \\
&\qquad \times \frac{f(\boldsymbol{Y}_{k+1}, \ldots, \boldsymbol{Y}_t \mid \mathcal{R}_{0,k-1}, A_{k,t-1}, \boldsymbol{G}_{k-1}, \boldsymbol{Y}_k, D \geq t)}{f(\boldsymbol{Y}_{k+1}, \ldots, \boldsymbol{Y}_t \mid \mathcal{R}_{0,k-1}, A_{k,t-1}, \boldsymbol{X}, \boldsymbol{Y}_k, D \geq t)} \\
&= f(\boldsymbol{G}_{k-1} \mid \mathcal{R}_{0,k-1}, A_{k,t-1}, \boldsymbol{X}, \boldsymbol{Y}_k, D \geq t) \qquad (73)
\end{aligned}
$$

Line (73) follows from equation (71). So, from equations (72) and (73),

$$
\begin{aligned}
&P(R_{0,t} = 1 \mid \mathcal{R}_{0,k-1}, A_{k,t-1}, \boldsymbol{X}, \boldsymbol{Y}_k, \boldsymbol{Y}_{k+1}, \ldots, \boldsymbol{Y}_t, D \geq t) \\
&= P(R_{0,t} = 1 \mid \mathcal{R}_{0,k-1}, A_{k,t-1}, \boldsymbol{X}, \boldsymbol{Y}_k, D \geq t)
\end{aligned}
$$

That is, mortal-cohort independent return holds. Similarly,

$$
\begin{aligned}
&P(D = t \mid \mathcal{R}_{0,k-1}, A_{k,t-1}, \boldsymbol{X}, \boldsymbol{Y}_k, \boldsymbol{Y}_{k+1}, \ldots, \boldsymbol{Y}_t, D \geq t) \\
&= \int P(D = t \mid \mathcal{R}_{0,k-1}, A_{k,t-1}, \boldsymbol{G}_{k-1}, \boldsymbol{Y}_k, \boldsymbol{Y}_{k+1}, \ldots, \boldsymbol{Y}_t, D \geq t) \\
&\qquad \times f(\boldsymbol{G}_{k-1} \mid \mathcal{R}_{0,k-1}, A_{k,t-1}, \boldsymbol{X}, \boldsymbol{Y}_k, \boldsymbol{Y}_{k+1}, \ldots \boldsymbol{Y}_t, D \geq t)\, d\boldsymbol{Y}_1 \ldots d\boldsymbol{Y}_{k-1} \\
&= \int P(D = t \mid \mathcal{R}_{0,k-1}, A_{k,t-1}, \boldsymbol{G}_{k-1}, \boldsymbol{Y}_k, D \geq t) \\
&\qquad \times f(\boldsymbol{G}_{k-1} \mid \mathcal{R}_{0,k-1}, A_{k,t-1}, \boldsymbol{X}, \boldsymbol{Y}_k, D \geq t)\, d\boldsymbol{G}_{k-1} \qquad (74) \\
&= P(D = t \mid \mathcal{R}_{0,k-1}, A_{k,t-1}, \boldsymbol{X}, \boldsymbol{Y}_k, D \geq t)
\end{aligned}
$$

So, independent death holds. Line (74) follows from strong independent death and by equation (73).

1.9 Proof of Theorem 9

In order also to be able to discuss the use of MVN imputation for mortal-cohort inference (below), we prove the following more general version of Theorem 9.

Theorem 10. *If equation (20), mortal-cohort dDTIC, mortal-cohort independent return and independent death hold, then for $k < s < t$,*

a) $f(\boldsymbol{Y}_t \mid \mathcal{R}_{0,k-1}, R_{0,k} = 1, R_{0,k+1} = \ldots = R_{0,t} = 0, \boldsymbol{X}, \boldsymbol{Y}_k, D \geq t) = f(\boldsymbol{Y}_t \mid \boldsymbol{X}, \boldsymbol{Y}_k)$,

b) $f(\boldsymbol{Y}_t \mid \mathcal{R}_{0,k-1}, R_{0,k} = 1, R_{0,k+1} = \ldots = R_{0,T} = 0, \boldsymbol{X}, \boldsymbol{Y}_k, D \geq t) = f(\boldsymbol{Y}_t \mid \boldsymbol{X}, \boldsymbol{Y}_k)$

c) $f(\boldsymbol{Y}_s \mid \mathcal{R}_{0,k-1}, R_{0,k} = 1, R_{0,k+1} = \ldots = R_{0,t-1} = 0, R_{0,t} = 1, \boldsymbol{X}, \boldsymbol{Y}_k, \boldsymbol{Y}_t, D \geq s)$

$= f(\boldsymbol{Y}_s \mid \boldsymbol{X}, \boldsymbol{Y}_k, \boldsymbol{Y}_t)$

When mortal-cohort strong independent return and strong independent death hold, $(\boldsymbol{X}, \boldsymbol{Y}_k)$ on the left-hand side of these equations can be replaced by $(\boldsymbol{X}, \boldsymbol{G}_k)$.

Proof

First, consider a).

$$\begin{aligned}
& f(\boldsymbol{Y}_t \mid \mathcal{R}_{0,k-1}, A_{k,t}, \boldsymbol{X}, \boldsymbol{Y}_k, D \geq t) \\
&= f(\boldsymbol{Y}_t \mid \mathcal{R}_{0,k-1}, A_{k,t-1}, \boldsymbol{X}, \boldsymbol{Y}_k, D \geq t) \\
&\qquad \times \frac{P(R_{0,t} = 0 \mid \mathcal{R}_{0,k-1}, A_{k,t-1}, \boldsymbol{X}, \boldsymbol{Y}_k, \boldsymbol{Y}_t, D \geq t)}{P(R_{0,t} = 0 \mid \mathcal{R}_{0,k-1}, A_{k,t-1}, \boldsymbol{X}, \boldsymbol{Y}_k, D \geq t)} \\
&= f(\boldsymbol{Y}_t \mid \mathcal{R}_{0,k-1}, A_{k,t-1}, \boldsymbol{X}, \boldsymbol{Y}_k, D \geq t) && (75) \\
&= f(\boldsymbol{Y}_t \mid \boldsymbol{X}, \boldsymbol{Y}_k) && (76)
\end{aligned}$$

Line (75) follows by mortal-cohort independent return. Line (76) follows from equation (65).

Second, consider b).

$$
\begin{aligned}
&f(\boldsymbol{Y}_t \mid \mathcal{R}_{0,k-1}, A_{k,T}, \boldsymbol{X}, \boldsymbol{Y}_k, D \geq t) \\
&= f(\boldsymbol{Y}_t \mid \mathcal{R}_{0,k-1}, A_{k,t-1}, \boldsymbol{X}, \boldsymbol{Y}_k, D \geq t) \\
&\qquad \times \frac{P(R_{0,t} = \ldots = R_{0,T} = 0 \mid \mathcal{R}_{0,k-1}, A_{k,t-1}, \boldsymbol{X}, \boldsymbol{Y}_k, \boldsymbol{Y}_t, D \geq t)}{P(R_{0,t} = \ldots = R_{0,T} = 0 \mid \mathcal{R}_{0,k-1}, A_{k,t-1}, \boldsymbol{X}, \boldsymbol{Y}_k, D \geq t)} \\
&= f(\boldsymbol{Y}_t \mid \mathcal{R}_{0,k-1}, A_{k,t-1}, \boldsymbol{X}, \boldsymbol{Y}_k, D \geq t) \qquad (77) \\
&= f(\boldsymbol{Y}_t \mid \boldsymbol{X}, \boldsymbol{Y}_k) \qquad (78)
\end{aligned}
$$

Line (77) follows by mortal-cohort independent return and independent death. Line (78) follows from equation (65).

Third, consider c).

$$
\begin{aligned}
&f(\boldsymbol{Y}_s \mid \mathcal{R}_{0,k-1}, A_{k,t-1}, R_{0,t} = 1, \boldsymbol{X}, \boldsymbol{Y}_k, \boldsymbol{Y}_t, D \geq s) \\
&= f(\boldsymbol{Y}_s \mid \mathcal{R}_{0,k-1}, A_{k,t-1}, R_{0,t} = 1, \boldsymbol{X}, \boldsymbol{Y}_k, \boldsymbol{Y}_t, D \geq t) \qquad (79) \\
&= f(\boldsymbol{Y}_s \mid \mathcal{R}_{0,k-1}, A_{k,t-1}, \boldsymbol{X}, \boldsymbol{Y}_k, \boldsymbol{Y}_t, D \geq t) \\
&\qquad \times \frac{P(R_{0,t} = 1 \mid \mathcal{R}_{0,k-1}, A_{k,t-1}, \boldsymbol{X}, \boldsymbol{Y}_k, \boldsymbol{Y}_s, \boldsymbol{Y}_t, D \geq t)}{P(R_{0,t} = 1 \mid \mathcal{R}_{0,k-1}, A_{k,t-1}, \boldsymbol{X}, \boldsymbol{Y}_k, \boldsymbol{Y}_t, D \geq t)} \\
&= f(\boldsymbol{Y}_s \mid \mathcal{R}_{0,k-1}, A_{k,t-1}, \boldsymbol{X}, \boldsymbol{Y}_k, \boldsymbol{Y}_t, D \geq t) \qquad (80) \\
&= \frac{f(\boldsymbol{Y}_s, \boldsymbol{Y}_t \mid \mathcal{R}_{0,k-1}, A_{k,t-1}, \boldsymbol{X}, \boldsymbol{Y}_k, D \geq t)}{f(\boldsymbol{Y}_t \mid \mathcal{R}_{0,k-1}, A_{k,t-1}, \boldsymbol{X}, \boldsymbol{Y}_k, D \geq t)} \\
&= \frac{f(\boldsymbol{Y}_s, \boldsymbol{Y}_t \mid \boldsymbol{X}, \boldsymbol{Y}_k)}{f(\boldsymbol{Y}_t \mid \boldsymbol{X}, \boldsymbol{Y}_k)} \qquad (81) \\
&= f(\boldsymbol{Y}_s \mid \boldsymbol{X}, \boldsymbol{Y}_k, \boldsymbol{Y}_t) \qquad (82)
\end{aligned}
$$

Line (79) follows because $R_{0,t} = 1$ implies $D \geq t$. Line (80) follows by mortal-cohort independent return. Line (81) follows from equation (65).

Appendix S2: Proof that dDTIC can be written as equation (10)

Assume equation (9) holds. By Bayesian Theorem,

$$
P(R_{0t} = 1 \mid \mathcal{R}_{0,t-1}, \mathcal{F}_T) = \frac{f(\boldsymbol{Y}_t, \ldots, \boldsymbol{Y}_T \mid \mathcal{R}_{0,t-1}, R_{0t}, \mathcal{F}_{t-1})}{f(\boldsymbol{Y}_t, \ldots, \boldsymbol{Y}_T \mid \mathcal{R}_{0,t-1}, \mathcal{F}_{t-1})} P(R_{0t} = 1 \mid \mathcal{R}_{0,t-1}, \mathcal{F}_{t-1})
$$

Now,

$$\begin{aligned} f(\boldsymbol{Y}_t, \dots, \boldsymbol{Y}_T \mid \mathcal{R}_{0,t-1}, R_{0t}, \mathcal{F}_{t-1}) &= \prod_{s=t}^{\top} f(\boldsymbol{Y}_s \mid \mathcal{R}_{0,t-1}, R_{0t}, \mathcal{F}_{s-1}) \\ &= \prod_{s=t}^{\top} f(\boldsymbol{Y}_s \mid \mathcal{F}_{s-1}) \\ &= f(\boldsymbol{Y}_t, \dots, \boldsymbol{Y}_T \mid \mathcal{F}_{t-1}) \end{aligned}$$

by equation (9). So, equation (10) holds.

Conversely, assume that equation (10) holds. Then

$$\begin{aligned} f(\boldsymbol{Y}_t \mid \mathcal{F}_{t-1}, \mathcal{R}_{0,t}) &= f(\boldsymbol{Y}_t \mid \mathcal{F}_{t-1}) \prod_{s=2}^{t} \frac{P(R_{0s} \mid \mathcal{F}_t, \mathcal{R}_{0,s-1})}{P(R_{0s} \mid \mathcal{F}_{t-1}, \mathcal{R}_{0,s-1})} \\ &= f(\boldsymbol{Y}_t \mid \mathcal{F}_{t-1}) \prod_{s=2}^{t} \frac{P(R_{0s} \mid \mathcal{F}_{s-1}, \mathcal{R}_{0,s-1})}{P(R_{0s} \mid \mathcal{F}_{s-1}, \mathcal{R}_{0,s-1})} \\ &= f(\boldsymbol{Y}_t \mid \mathcal{F}_{t-1}) \end{aligned}$$

by equation (10).

Appendix S3: Proof of equation (19)

Before giving a formal proof, we provide some intuition as to why this constraint arises. The unstructured MVN model can be reparameterised as

$$\begin{bmatrix} \boldsymbol{Y}_1 \\ \boldsymbol{X} \end{bmatrix} \sim N\left\{ \begin{bmatrix} \boldsymbol{\mu}_1 \\ \boldsymbol{\mu}_{T+1} \end{bmatrix}, \begin{bmatrix} \boldsymbol{\Sigma}_{1,1} & \boldsymbol{\Sigma}_{1,T+1}^{\top} \\ \boldsymbol{\Sigma}_{1,T+1} & \boldsymbol{\Sigma}_{T+1,T+1} \end{bmatrix} \right\} \tag{83}$$

$$\boldsymbol{Y}_t = \boldsymbol{\alpha}_t + \boldsymbol{\beta}_t^{\top} \boldsymbol{Y}_{t-1} + \sum_{j=1}^{t-2} \boldsymbol{\delta}_{tj}^{\top} \boldsymbol{Y}_j + \boldsymbol{\gamma}_t^{\top} \boldsymbol{X} + \boldsymbol{\epsilon}_t \tag{84}$$

$$\boldsymbol{\epsilon}_t \mid \boldsymbol{Y}_1, \dots, \boldsymbol{Y}_{t-1}, \boldsymbol{X} \sim \text{Normal}(\boldsymbol{0}, \boldsymbol{\sigma}_t) \quad (t \geq 2) \tag{85}$$

If it assumed that equation (2) holds, then each $\boldsymbol{\delta}_{tj}$ must equal zero. Since there are $(T-1)(T-2)/2$ matrices $\boldsymbol{\delta}_{tj}$, each of which has m^2 elements, constraining $\boldsymbol{\delta}_{tj} = \boldsymbol{0}$ reduces the number of free parameters by $(T-1)(T-2)m^2/2$. Returning to equation (19), we note that there are $T(T-1)/2$ matrices $\boldsymbol{\Sigma}_{st}$ $(s < t)$ and $T-1$ matrices $\boldsymbol{\beta}_t$, and each of these matrices has m^2 elements, so the constraint of equation (19)

reduces the number of free parameters by the same number: $(T-1)(T-2)m^2/2$. The relation between the parameters of the original and reparameterised models is as follows. For $t \geq 2$,

$$\begin{aligned}
\boldsymbol{\mu}_t &= \boldsymbol{\alpha}_t + \boldsymbol{\beta}_t^\top \boldsymbol{\mu}_{t-1} + \boldsymbol{\gamma}_t^\top \boldsymbol{X} && (86)\\
\boldsymbol{\Sigma}_{t,T+1} &= \boldsymbol{\beta}_t^\top \boldsymbol{\Sigma}_{t-1,T+1} + \boldsymbol{\gamma}_t^\top \boldsymbol{\Sigma}_{T+1,T+1} && (87)\\
\boldsymbol{\Sigma}_{t,t} &= \boldsymbol{\beta}_t^\top \boldsymbol{\Sigma}_{t-1,t-1} \boldsymbol{\beta}_t + \boldsymbol{\gamma}_t^\top \boldsymbol{\Sigma}_{T+1,T+1} \boldsymbol{\gamma}_t + 2\boldsymbol{\beta}_t^\top \boldsymbol{\Sigma}_{t-1,T+1} \boldsymbol{\gamma}_t + \boldsymbol{\sigma}_t && (88)
\end{aligned}$$

and, for $1 \leq s < t \leq T$, $\boldsymbol{\Sigma}_{s,t}$ is given by equation (19). Conversely, for $t \geq 2$, $\boldsymbol{\beta}_t$, $\boldsymbol{\gamma}_t$ and $\boldsymbol{\alpha}_t$ are given by equations (15)–(17) without the hats, and

$$\boldsymbol{\sigma}_t = \boldsymbol{\Sigma}_{t,t} - \boldsymbol{\beta}_t^\top \boldsymbol{\Sigma}_{t-1,t-1} \boldsymbol{\beta}_t - \boldsymbol{\gamma}_t^\top \boldsymbol{\Sigma}_{T+1,T+1} \boldsymbol{\gamma}_t - 2\boldsymbol{\beta}_t^\top \boldsymbol{\Sigma}_{t-1,T+1} \boldsymbol{\gamma}_t \qquad (89)$$

We now provide a formal proof of equation (19).

For $1 \leq s < t \leq T$, we have from equation (1) that

$$\begin{aligned}
\boldsymbol{Y}_t &= \boldsymbol{\alpha}_t + \boldsymbol{\beta}_t^\top (\boldsymbol{\alpha}_{t-1} + \boldsymbol{\beta}_{t-1}^\top \boldsymbol{Y}_{t-2} + \boldsymbol{\gamma}_{t-1}^\top \boldsymbol{X} + \boldsymbol{\epsilon}_{t-1}) + \boldsymbol{\gamma}_t^\top \boldsymbol{X} + \boldsymbol{\epsilon}_t\\
&= \sum_{j=s+1}^{t-1} \{\boldsymbol{\beta}_t^\top \boldsymbol{\beta}_{t-1}^\top \ldots \boldsymbol{\beta}_{j+1}^\top (\boldsymbol{\alpha}_j + \boldsymbol{\gamma}_j^\top \boldsymbol{X})\} + \boldsymbol{\alpha}_t + \boldsymbol{\gamma}_t^\top \boldsymbol{X} + \boldsymbol{\beta}_t^\top \boldsymbol{\beta}_{t-1}^\top \ldots, \boldsymbol{\beta}_{s+1}^\top \boldsymbol{Y}_s\\
&\quad + \sum_{j=s+1}^{t-1} \{\boldsymbol{\beta}_t^\top \boldsymbol{\beta}_{t-1}^\top \ldots \boldsymbol{\beta}_{j+1}^\top \boldsymbol{\epsilon}_j\} + \boldsymbol{\epsilon}_t
\end{aligned}$$

So,

$$\begin{aligned}
E(\boldsymbol{Y}_t \boldsymbol{Y}_s^\top) &= \sum_{j=s+1}^{t-1} [\boldsymbol{\beta}_t^\top \boldsymbol{\beta}_{t-1}^\top \ldots \boldsymbol{\beta}_{j+1}^\top \{\boldsymbol{\alpha}_j E(\boldsymbol{Y}_s)^\top + \boldsymbol{\gamma}_j^\top E(\boldsymbol{X}\boldsymbol{Y}_s^\top)\}] + \boldsymbol{\alpha}_t E(\boldsymbol{Y}_s)^\top\\
&\quad + \boldsymbol{\gamma}_t^\top E(\boldsymbol{X}\boldsymbol{Y}_s^\top) + \boldsymbol{\beta}_t^\top \boldsymbol{\beta}_{t-1}^\top \ldots \boldsymbol{\beta}_{s+1}^\top E(\boldsymbol{Y}_s \boldsymbol{Y}_s^\top)
\end{aligned}$$

[Throughout this proof, terms beginning $\sum_{j=s+1}^{t-1}$ should be interpreted as being equal to zero if $s = t-1$.]

Consequently, using $\prod_{j=a}^{b}\boldsymbol{\beta}_j^\top$ as shorthand for $\boldsymbol{\beta}_b^\top\boldsymbol{\beta}_{b-1}^\top\ldots\boldsymbol{\beta}_a^\top$, we have

$$
\begin{aligned}
\boldsymbol{\Sigma}_{t,s} &= \sum_{j=s+1}^{t-1}\prod_{k=j+1}^{t}\boldsymbol{\beta}_k^\top\{\boldsymbol{\alpha}_j\boldsymbol{\mu}_s^\top+\boldsymbol{\gamma}_j^\top(\boldsymbol{\Sigma}_{T+1,s}+\boldsymbol{\mu}_{T+1}\boldsymbol{\mu}_s^\top)\}+\boldsymbol{\alpha}_t\boldsymbol{\mu}_s^\top+\boldsymbol{\gamma}_t^\top(\boldsymbol{\Sigma}_{T+1,s}+\boldsymbol{\mu}_{T+1}\boldsymbol{\mu}_s^\top) \\
&\quad + \prod_{k=s+1}^{t}\boldsymbol{\beta}_k^\top(\boldsymbol{\Sigma}_{s,s}+\boldsymbol{\mu}_s\boldsymbol{\mu}_s^\top)-\boldsymbol{\mu}_t\boldsymbol{\mu}_s^\top \\
&= \sum_{j=s+1}^{t-1}\prod_{k=j+1}^{t}\boldsymbol{\beta}_k^\top\{(\boldsymbol{\mu}_j-\boldsymbol{\beta}_j^\top\boldsymbol{\mu}_{j-1})\boldsymbol{\mu}_s^\top+\boldsymbol{\gamma}_j^\top\boldsymbol{\Sigma}_{T+1,s}\}+(\boldsymbol{\mu}_t-\boldsymbol{\beta}_t^\top\boldsymbol{\mu}_{t-1})\boldsymbol{\mu}_s^\top \\
&\quad +\boldsymbol{\gamma}_t^\top\boldsymbol{\Sigma}_{T+1,s}+\prod_{k=s+1}^{t}\boldsymbol{\beta}_k^\top(\boldsymbol{\Sigma}_{s,s}+\boldsymbol{\mu}_s\boldsymbol{\mu}_s^\top)-\boldsymbol{\mu}_t\boldsymbol{\mu}_s^\top \\
&= \left(\boldsymbol{\mu}_t-\prod_{k=s+1}^{t}\boldsymbol{\beta}_k^\top\boldsymbol{\mu}_s\right)\boldsymbol{\mu}_s^\top+\sum_{j=s+1}^{t-1}\prod_{k=j+1}^{t}\boldsymbol{\beta}_k^\top\boldsymbol{\gamma}_j^\top\boldsymbol{\Sigma}_{T+1,s}+\boldsymbol{\gamma}_t^\top\boldsymbol{\Sigma}_{T+1,s} \\
&\quad + \prod_{k=s+1}^{t}\boldsymbol{\beta}_k^\top(\boldsymbol{\Sigma}_{s,s}+\boldsymbol{\mu}_s\boldsymbol{\mu}_s^\top)-\boldsymbol{\mu}_t\boldsymbol{\mu}_s^\top \\
&= -\prod_{k=s+1}^{t}\boldsymbol{\beta}_k^\top\boldsymbol{\mu}_s\boldsymbol{\mu}_s^\top+\sum_{j=s+1}^{t-1}\prod_{k=j+1}^{t}\boldsymbol{\beta}_k^\top(\boldsymbol{\Sigma}_{j,T+1}-\boldsymbol{\beta}_j^\top\boldsymbol{\Sigma}_{j-1,T+1})\boldsymbol{\Sigma}_{T+1,T+1}^{-1}\boldsymbol{\Sigma}_{T+1,s} \\
&\quad +(\boldsymbol{\Sigma}_{t,T+1}-\boldsymbol{\beta}_t^\top\boldsymbol{\Sigma}_{t-1,T+1})\boldsymbol{\Sigma}_{T+1,T+1}^{-1}\boldsymbol{\Sigma}_{T+1,s}+\prod_{k=s+1}^{t}\boldsymbol{\beta}_k^\top(\boldsymbol{\Sigma}_{s,s}+\boldsymbol{\mu}_s\boldsymbol{\mu}_s^\top) \\
&= -\prod_{k=s+1}^{t}\boldsymbol{\beta}_k^\top\boldsymbol{\mu}_s\boldsymbol{\mu}_s^\top+\left(\boldsymbol{\Sigma}_{t,T+1}-\prod_{k=s+1}^{t}\boldsymbol{\beta}_k^\top\boldsymbol{\Sigma}_{s,T+1}\right)\boldsymbol{\Sigma}_{T+1,T+1}^{-1}\boldsymbol{\Sigma}_{T+1,s} \\
&\quad + \prod_{k=s+1}^{t}\boldsymbol{\beta}_k^\top(\boldsymbol{\Sigma}_{s,s}+\boldsymbol{\mu}_s\boldsymbol{\mu}_s^\top) \\
&= \boldsymbol{\Sigma}_{t,T+1}\boldsymbol{\Sigma}_{T+1,T+1}^{-1}\boldsymbol{\Sigma}_{T+1,s}+\prod_{k=s+1}^{t}\boldsymbol{\beta}_k^\top(\boldsymbol{\Sigma}_{s,s}-\boldsymbol{\Sigma}_{s,T+1}\boldsymbol{\Sigma}_{T+1,T+1}^{-1}\boldsymbol{\Sigma}_{T+1,s}) \qquad (90)
\end{aligned}
$$

using equations (54) and (55). Note that equation (90) still holds when $\boldsymbol{\beta}_t$ is constrained to equal $\boldsymbol{I}$.

Appendix S4: Random-walk MVN (rMVN) methods

Here we describe the LI-rMVN imputation and rMVN imputation methods introduced in Section 4.3.

The model defined by $(\boldsymbol{Y}_1^\top, \ldots, \boldsymbol{Y}_T^\top, \boldsymbol{X}^\top)^\top \sim \text{Normal}(\boldsymbol{\mu}, \boldsymbol{\Sigma})$ and equation (19) with $\boldsymbol{\beta}_t = \boldsymbol{I}$ can be reparameterised as equations (83)–(85) with $\boldsymbol{\delta}_{tj} = \boldsymbol{0}$ and $\boldsymbol{\beta}_t = \boldsymbol{I}$. The relation between the parameters of the original and reparameterised models is given by equations (86)–(88) and by equations (16) and (17) without the hats and (89), all with $\boldsymbol{\beta}_t = \boldsymbol{I}$.

This model can be fitted by maximum likelihood to the outcomes $\boldsymbol{Y}_t + \boldsymbol{e}_t$ observed with error, thus treating them as though they were the underlying outcomes $\boldsymbol{Y}_t$, and ignoring the missingness mechanism (see Section S5 for fitting algorithm). We call this the 'random-walk MVN (rMVN)' method.

Theorem 11. *If the increments model of equation (2), the dDTIC assumption of equation (9) and the independent return assumption of equation (8) hold and* $\boldsymbol{\beta}_t = \boldsymbol{I}$*, then the random-walk MVN method yields consistent estimates of* $\boldsymbol{\mu}_1$*,* $\boldsymbol{\mu}_{T+1}$*,* $\boldsymbol{\Sigma}_{1,T+1}$*,* $\boldsymbol{\Sigma}_{T+1,T+1}$*,* $\boldsymbol{\alpha}_t$ *and* $\boldsymbol{\gamma}_t$ *(*$t = 2, \ldots, T$*).*

Like Theorem 4, Theorem 11 does not require that the data actually be normally distributed. Equations (86) and (87) can then be used to obtain consistent estimates of $\boldsymbol{\mu}_t$ and $\boldsymbol{\Sigma}_{t,T+1}$. Note that the maximum likelihood estimates of $\boldsymbol{\Sigma}_{t,t}$ obtained using equation (88) are not consistent unless there is no measurement error. For example, the maximum likelihood estimator of $\boldsymbol{\Sigma}_{11}$ converges to $\text{Var}(\boldsymbol{Y}_1) + \text{Var}(\boldsymbol{e}_1)$ as $N \to \infty$, rather than to $\boldsymbol{\Sigma}_{11} = \text{Var}(\boldsymbol{Y}_1)$. This is not a problem for LI imputation using the random-walk MVN estimates of $\boldsymbol{\alpha}_t$ and $\boldsymbol{\gamma}_t$ ('LI-rMVN imputation'). It is also not a problem when imputation is carried out using equation (18) with the random-walk MVN estimates of $\boldsymbol{\mu}$ and $\boldsymbol{\Sigma}$ ('rMVN imputation'), because the complete-data maximum likelihood estimator of the parameters of a linear regression of $\boldsymbol{Y}$ on t and/or $\boldsymbol{X}$ is not a function of $\hat{\boldsymbol{\Sigma}}_{t,t}$ (see Appendix S6 for details).

Proof of Theorem 11

To avoid confusion in this proof, we shall denote the true values of $\boldsymbol{\alpha}_t$ and $\boldsymbol{\gamma}_t$ in equation (2) as $\boldsymbol{\alpha}_{0t}$ and $\boldsymbol{\gamma}_{0t}$, and the true values of $\boldsymbol{\mu}_t = E(\boldsymbol{Y}_t)$ and $\boldsymbol{\Sigma}_{st} = \mathrm{Cov}(\boldsymbol{Y}_s, \boldsymbol{Y}_t)$ as $\boldsymbol{\mu}_{0t}$ and $\boldsymbol{\Sigma}_{0st}$. The model being fitted is

$$\begin{bmatrix} \boldsymbol{Y}_1 + \boldsymbol{e}_1 \\ \boldsymbol{X} \end{bmatrix} \sim N\left\{ \begin{bmatrix} \boldsymbol{\mu}_1 \\ \boldsymbol{\mu}_{T+1} \end{bmatrix}, \begin{bmatrix} \boldsymbol{\Sigma}_{1,1} & \boldsymbol{\Sigma}_{1,T+1}^\top \\ \boldsymbol{\Sigma}_{1,T+1} & \boldsymbol{\Sigma}_{T+1,T+1} \end{bmatrix} \right\} \tag{91}$$

$$\boldsymbol{Y}_t + \boldsymbol{e}_t \mid \boldsymbol{Y}_s + \boldsymbol{e}_s, \boldsymbol{X} \sim N\left(\sum_{j=s+1}^{t} (\boldsymbol{\alpha}_j + \boldsymbol{\gamma}_j^\top \boldsymbol{X}) + \boldsymbol{Y}_s + \boldsymbol{e}_s, \sum_{j=s+1}^{t} \boldsymbol{\sigma}_j \right) \tag{92}$$

$$(\forall t > s \geq 1)$$

Note that we are not assuming in this proof that the model given by equations (91) and (92) describes the true relation between the random variables.

Equation (2) with $\boldsymbol{\beta}_t = \boldsymbol{I}$ implies that

$$E(\boldsymbol{Y}_t \mid \boldsymbol{Y}_s, \boldsymbol{X}) = \sum_{j=s+1}^{t} (\boldsymbol{\alpha}_{0j} + \boldsymbol{\gamma}_{0j}^\top \boldsymbol{X}) + \boldsymbol{Y}_s \qquad \forall t > s \tag{93}$$

Note that we are assuming in this proof that equation (93) does describe the true relation between the random variables.

Let $\tilde{\boldsymbol{x}}$ denote the $p \times m$ matrix in which each of the m columns equals $\boldsymbol{x}$. Let L denote an individual's contribution to the log-likelihood function of the model defined by equations (91) and (92), and let $\boldsymbol{\theta} = (\boldsymbol{\mu}_1, \boldsymbol{\mu}_{T+1}, \boldsymbol{\Sigma}_{1,1}, \boldsymbol{\Sigma}_{1,T+1}, \boldsymbol{\Sigma}_{T+1,T+1}, \boldsymbol{\alpha}_2, \boldsymbol{\gamma}_2, \boldsymbol{\sigma}_2, \ldots, \boldsymbol{\alpha}_T, \boldsymbol{\gamma}_T, \boldsymbol{\sigma}_T)^\top$. The contribution of an individual to the score function $\partial L/\partial \boldsymbol{\theta}$ of this model is (as in the proof of Theorem 4)

$$\begin{aligned} \frac{\partial L(\boldsymbol{\theta})}{\partial \boldsymbol{\theta}} &= \boldsymbol{h}_1(\boldsymbol{x}, \boldsymbol{y}_1 + \boldsymbol{e}_1; \boldsymbol{\theta}) \\ &\quad + \sum_{t=2}^{\top} \sum_{s=1}^{t-1} \left\{ \begin{array}{c} I(R_{0,t} = 1, R_{0,t-1} = \ldots = R_{0,s+1} = 0, R_{0,s} = 1) \\ \times \boldsymbol{h}_{t,s}(\boldsymbol{y}_t + \boldsymbol{e}_t \mid \boldsymbol{y}_s + \boldsymbol{e}_s, \boldsymbol{x};\ \boldsymbol{\theta}) \end{array} \right\} \end{aligned} \tag{94}$$

where the form of $\boldsymbol{h}_1(\boldsymbol{x}, \boldsymbol{y}_1 + \boldsymbol{e}_1;\ \boldsymbol{\theta})$ is evident from equation (91) and, for $t > s \geq 1$, the elements of $\boldsymbol{h}_{t,s}$ are (from equation (92))

$$\boldsymbol{1}_m^\top \left(\sum_{j=s+1}^{t} \boldsymbol{\sigma}_j \right)^{-1} \left\{ \boldsymbol{y}_t + \boldsymbol{e}_t - \boldsymbol{y}_s - \boldsymbol{e}_s - \sum_{j=s+1}^{t} (\boldsymbol{\alpha}_j + \boldsymbol{\gamma}_j^\top \boldsymbol{x}) \right\}$$

for elements corresponding to $\boldsymbol{\alpha}_j$ with $s+1 \le j \le t$, and

$$\tilde{\boldsymbol{x}} \left(\sum_{j=s+1}^{t} \boldsymbol{\sigma}_j \right)^{-1} \left\{ \boldsymbol{y}_t + \boldsymbol{e}_t - \boldsymbol{y}_s - \boldsymbol{e}_s - \sum_{j=s+1}^{t} (\boldsymbol{\alpha}_j + \boldsymbol{\gamma}_j^\top \boldsymbol{x}) \right\}$$

for elements corresponding to $\boldsymbol{\gamma}_j$ with $s+1 \le j \le t$, and zero for all other elements. Clearly, the elements of $\boldsymbol{h}_1(\boldsymbol{x}, \boldsymbol{y}_1 + \boldsymbol{e}_1;\ \boldsymbol{\theta})$ corresponding to $(\boldsymbol{\alpha}_2, \boldsymbol{\gamma}_2, \ldots, \boldsymbol{\alpha}_T, \boldsymbol{\gamma}_T)^\top$ are all zero.

Let $\hat{\boldsymbol{\theta}}$ denote the solution of $\partial L(\boldsymbol{\theta})/\partial \boldsymbol{\theta}$. Let $\boldsymbol{\theta}_0 = (\boldsymbol{\mu}_{01}, \boldsymbol{\mu}_{0,T+1},\ \boldsymbol{\Sigma}_{011} + \mathrm{Var}(\boldsymbol{e}_1),$ $\boldsymbol{\Sigma}_{01,T+1},\ \boldsymbol{\Sigma}_{0T+1,T+1}, \boldsymbol{\alpha}_{02},\ \boldsymbol{\gamma}_{02}, \boldsymbol{\sigma}_2^*, \ldots, \boldsymbol{\alpha}_{0T}, \boldsymbol{\gamma}_{0T},\ \boldsymbol{\sigma}_T^*)^\top$ for some $\boldsymbol{\sigma}_2^*, \ldots, \boldsymbol{\sigma}_T^*$.

In order to show that $\hat{\boldsymbol{\theta}}$ converges in probability to $\boldsymbol{\theta}_0$ as $N \to \infty$, it suffices to show that $E\{\partial L(\boldsymbol{\theta})/\partial \boldsymbol{\theta}\}|_{\theta=\theta_0} = \mathbf{0}$. It is obvious that $E\{\boldsymbol{h}_1(\boldsymbol{X}, \boldsymbol{Y}_1 + \boldsymbol{e}_1;\ \boldsymbol{\theta}_0)\} = \mathbf{0}$ at $\boldsymbol{\theta}_0$. So, it suffices to show that

$$E\left\{ \boldsymbol{Y}_t + \boldsymbol{e}_t - \boldsymbol{Y}_s - \boldsymbol{e}_s - \sum_{j=s+1}^{t} (\boldsymbol{\alpha}_{0j} + \boldsymbol{\gamma}_{0j}^\top \boldsymbol{X}) \mid \boldsymbol{X}, A_{s,t-1}, R_{0,t} = 1 \right\} = \mathbf{0}.$$

for all $t > s \ge 1$. I now show this.

$$\begin{aligned}
& E\left\{ \boldsymbol{Y}_t + \boldsymbol{e}_t - \boldsymbol{Y}_s - \boldsymbol{e}_s - \sum_{j=s+1}^{t} (\boldsymbol{\alpha}_{0j} + \boldsymbol{\gamma}_{0j}^\top \boldsymbol{X}) \mid \boldsymbol{X}, A_{s,t-1}, R_{0,t} = 1 \right\} \\
&= E\left\{ \boldsymbol{Y}_t - \boldsymbol{Y}_s - \sum_{j=s+1}^{t} (\boldsymbol{\alpha}_{0j} + \boldsymbol{\gamma}_{0j}^\top \boldsymbol{X}) \mid \boldsymbol{X}, A_{s,t-1}, R_{0,t} = 1 \right\} \qquad (95) \\
&= E\left[E\left\{ \boldsymbol{Y}_t - \boldsymbol{Y}_s - \sum_{j=s+1}^{t} (\boldsymbol{\alpha}_{0j} + \boldsymbol{\gamma}_{0j}^\top \boldsymbol{X}) \mid \boldsymbol{X}, \boldsymbol{Y}_s, A_{s,t-1}, R_{0,t} = 1 \right\} \mid \boldsymbol{X}, A_{s,t-1}, R_{0,t} = 1 \right] \\
&= E\left[E\left\{ \boldsymbol{Y}_t - \boldsymbol{Y}_s - \sum_{j=s+1}^{t} (\boldsymbol{\alpha}_{0j} + \boldsymbol{\gamma}_{0j}^\top \boldsymbol{X}) \mid \boldsymbol{X}, \boldsymbol{Y}_s \right\} \mid A_{s,t-1}, R_{0,t} = 1 \right] \qquad (96) \\
&= E\left[\mathbf{0} \mid A_{s,t-1}, R_{0,t} = 1 \right] \qquad (97) \\
&= \mathbf{0}
\end{aligned}$$

Equation (95) uses the assumptions that $\{\boldsymbol{e}_t : t = 1, \ldots, T\}$ is independent of all other processes, $\boldsymbol{e}_t$ is independent of $\boldsymbol{e}_s$ for all $t \neq s$, and $E(\boldsymbol{e}_t) = \mathbf{0}$ for all t. Equation (96) follows from equation (14) with $\boldsymbol{G}_k$ replaced by $(\boldsymbol{X}, \boldsymbol{Y}_k)$. Equation (97) uses equation (93).

Appendix S5: EM algorithm for MVN methods

As explained in Section 4 of our paper, the standard (unstructured) MVN method does not respect the constraints on the variance given by equation (19). Schafer (1997) described how to fit this MVN model using an EM algorithm, and this has been implemented in the norm package of R. For the autoregressive MVN method, we need to impose this constraint at the M step of the algorithm. The norm package can still be used to carry out the E step of the EM algorithm, but the M step needs to be modified.

The linearity of the log likelihood function of an MVN model means that the M step involves simply applying complete-data maximum likelihood estimators of $\boldsymbol{\mu}$ and $\boldsymbol{\Sigma}$ to the expected sufficient statistics calculated at the E step. The complete data sufficient statistics are the sample mean and variance of $(\boldsymbol{Y}_1, \ldots, \boldsymbol{Y}_T, \boldsymbol{X})$. For the MVN model with unstructured variance matrix, the complete-data maximum likelihood estimators are given by Schafer (1997) on page 149–150. For the autoregressive MVN model, the complete-data maximum likelihood estimators of $\boldsymbol{\mu}$, $\boldsymbol{\Sigma}_{t,t}$ and $\boldsymbol{\Sigma}_{T+1,t}$ ($t = 1, \ldots, T+1$) are the same as for the unstructured MVN model. The only difference is for $\boldsymbol{\Sigma}_{t,s}$ ($1 \leq s, t \leq T$ with $s \neq t$). For the autoregressive MVN model the complete-data maximum likelihood estimator of $\boldsymbol{\Sigma}_{t,s}$ ($1 \leq s < t \leq T$) is

$$\hat{\boldsymbol{\Sigma}}_{t,s} = \hat{\boldsymbol{\Sigma}}_{t,T+1}\hat{\boldsymbol{\Sigma}}^{-1}_{T+1,T+1}\hat{\boldsymbol{\Sigma}}_{T+1,s} + \boldsymbol{\beta}^{*\top}_t \boldsymbol{\beta}^{*\top}_{t-1} \ldots \boldsymbol{\beta}^{*\top}_{s+1}(\hat{\boldsymbol{\Sigma}}_{s,s} - \hat{\boldsymbol{\Sigma}}_{s,T+1}\hat{\boldsymbol{\Sigma}}^{-1}_{T+1,T+1}\hat{\boldsymbol{\Sigma}}_{T+1,s})$$

where

$$\boldsymbol{\beta}^*_t = (\boldsymbol{c}_{t-1,t-1} - \boldsymbol{c}_{t-1,T+1}\boldsymbol{c}^{-1}_{T+1,T+1}\boldsymbol{c}_{T+1,t-1})^{-1}(\boldsymbol{c}_{t-1,t} - \boldsymbol{c}_{t-1,T+1}\boldsymbol{c}^{-1}_{T+1,T+1}\boldsymbol{c}_{T+1,t})$$

with $\boldsymbol{c}_{s,t}$ ($1 \leq s, t \leq T$) denoting the sample covariance of $\boldsymbol{Y}_s$ and $\boldsymbol{Y}_t$, and $\boldsymbol{c}_{T+1,t}$ denoting the sample covariance of $\boldsymbol{X}$ and $\boldsymbol{Y}_t$, and $\boldsymbol{c}_{T+1,T+1}$ denoting the sample variance of $\boldsymbol{X}$.

For the random-walk MVN model, the constraints on the variance given by equation (19) with $\boldsymbol{\beta} = \boldsymbol{I}$ need to be imposed. Again, the norm package can be used

to carry out the E step of the EM algorithm, but the M step needs to be modified. The M step can be carried out by fitting the model given by equations (83)–(85) with $\boldsymbol{\delta}_{tj} = \mathbf{0}$ and $\boldsymbol{\beta}_t = \boldsymbol{I}$ and then calculating $\hat{\boldsymbol{\mu}}$ and $\hat{\boldsymbol{\Sigma}}$ using equations (86)–(88). However, we did not actually do this. Instead, we used a Newton-Raphson algorithm to maximise the observed-data likelihood directly.

Appendix S6: MVN imputation as a method for estimating parameters of a linear regression model

For simplicity, assume that $\boldsymbol{Y}$ is univariate (i.e. $m = 1$). However, in the following, $\boldsymbol{Y}$ could easily be replaced by one of the m univariate elements of a vector $\boldsymbol{Y}$.

First, we shall show that the complete-data maximum likelihood estimates of the linear regression model

$$\boldsymbol{Y}_{it} = \psi_0 + \psi_1 t + \boldsymbol{\psi}_2^\top \boldsymbol{X} + \text{Normal}(0, \tau^2) \tag{98}$$

are functions of the complete-data statistics $\bar{\boldsymbol{X}}$, $\bar{Y}_t$, $\boldsymbol{c}_{T+1,t}$ and $\boldsymbol{c}_{T+1,T+1}$. Second, we shall show that the values of $\bar{\boldsymbol{X}}$, $\bar{Y}_t$, $\boldsymbol{c}_{T+1,t}$ and $\boldsymbol{c}_{T+1,T+1}$ ($t = 1, \ldots, T$) in the imputed dataset are equal to, respectively, $\hat{\boldsymbol{\mu}}_{T+1}$, $\hat{\boldsymbol{\mu}}_t$, $\hat{\boldsymbol{\Sigma}}_{T+1,t}$ and $\hat{\boldsymbol{\Sigma}}_{T+1,T+1}$. This implies that performing MVN imputation and then fitting the linear regression model of equation (98) to the imputed data gives the same estimates of ψ_0, ψ_1 and ψ_2 as applying the forementioned functions to $\hat{\boldsymbol{\mu}}_{T+1}$, $\hat{\boldsymbol{\mu}}_t$, $\hat{\boldsymbol{\Sigma}}_{T+1,t}$ and $\hat{\boldsymbol{\Sigma}}_{T+1,T+1}$ directly.

Let $\boldsymbol{\psi} = (\psi_0, \psi_1, \boldsymbol{\psi}_2^\top)^\top$. Then

$$\hat{\boldsymbol{\psi}} = (\boldsymbol{Z}^\top \boldsymbol{Z})^{-1} \boldsymbol{Z}^\top \boldsymbol{W} \tag{99}$$

where

$$\boldsymbol{Z} = \begin{bmatrix} 1 & 1 & \boldsymbol{X}_1^\top \\ 1 & 2 & \boldsymbol{X}_1^\top \\ \vdots & \vdots & \vdots \\ 1 & T & \boldsymbol{X}_1^\top \\ 2 & 1 & \boldsymbol{X}_2^\top \\ 2 & 2 & \boldsymbol{X}_2^\top \\ \vdots & \vdots & \vdots \\ 2 & T & \boldsymbol{X}_2^\top \\ \vdots & \vdots & \vdots \\ N & 1 & \boldsymbol{X}_N^\top \\ N & 2 & \boldsymbol{X}_N^\top \\ \vdots & \vdots & \vdots \\ N & T & \boldsymbol{X}_N^\top \end{bmatrix} \quad \boldsymbol{W} = \begin{bmatrix} Y_{11} \\ Y_{12} \\ \vdots \\ Y_{1T} \\ Y_{21} \\ Y_{22} \\ \vdots \\ Y_{2T} \\ \vdots \\ Y_{N1} \\ Y_{N2} \\ \vdots \\ Y_{NT} \end{bmatrix}$$

So,

$$\boldsymbol{Z}^\top \boldsymbol{Z} = \begin{bmatrix} NT & N\sum_{t=1}^{T} t & TN\bar{\boldsymbol{X}}^\top \\ N\sum_{t=1}^{T} t & N\sum_{t=1}^{T} t^2 & N\sum_{t=1}^{T} t\bar{\boldsymbol{X}}^\top \\ TN\bar{\boldsymbol{X}} & N\sum_{t=1}^{T} t\bar{\boldsymbol{X}} & N(\boldsymbol{c}_{T+1,T+1} + \bar{\boldsymbol{X}}\bar{\boldsymbol{X}}^\top) \end{bmatrix} \tag{100}$$

and

$$\boldsymbol{Z}^\top \boldsymbol{W} = \begin{bmatrix} N\sum_{t=1}^{T} \bar{Y}_t \\ N\sum_{t=1}^{T} t\bar{Y}_t \\ N\sum_{t=1}^{T} (\boldsymbol{c}_{T+1,t} + \bar{\boldsymbol{X}}\bar{Y}_t) \end{bmatrix} \tag{101}$$

Therefore $\hat{\boldsymbol{\psi}}$ is a function of $\bar{\boldsymbol{X}}$, $\bar{Y}_t$, $\boldsymbol{c}_{T+1,t}$ and $\boldsymbol{c}_{T+1,T+1}$.

At convergence of the EM algorithm for fitting the MVN model (whether unstructured, autoregressive or random-walk) the expected values of $\bar{\boldsymbol{X}}$, $\bar{Y}_t$, $\boldsymbol{c}_{T+1,t}$ and $\boldsymbol{c}_{T+1,T+1}$ given the observed data are equal to $\hat{\boldsymbol{\mu}}_{T+1}$, $\hat{\boldsymbol{\mu}}_t$, $\hat{\boldsymbol{\Sigma}}_{T+1,t}$ and $\hat{\boldsymbol{\Sigma}}_{T+1,T+1}$ (see Section S5). Since $\boldsymbol{X}$ is fully observed, $\bar{\boldsymbol{X}}$ and $\boldsymbol{c}_{T+1,T+1}$ are observed, and $\hat{\boldsymbol{\mu}}_{T+1}$ and $\hat{\boldsymbol{\Sigma}}_{T+1,T+1}$ are equal to them. The values of $\bar{Y}_t$ and $\boldsymbol{c}_{T+1,t}$ are not observed, but because $\boldsymbol{X}$ is fully observed their expected values given the observed data can be calculated by application of equation (18). Since this is precisely what is done in MVN imputation, the values of $\bar{Y}_t$ and $\boldsymbol{c}_{T+1,t}$ calculated from the imputed data will equal $\hat{\boldsymbol{\mu}}_t$ and $\hat{\boldsymbol{\Sigma}}_{T+1,t}$. Thus, whether one applies equations (99)–(101) to the imputed data or substitutes $\hat{\boldsymbol{\mu}}_{T+1}$, $\hat{\boldsymbol{\mu}}_t$, $\hat{\boldsymbol{\Sigma}}_{T+1,t}$ and $\hat{\boldsymbol{\Sigma}}_{T+1,T+1}$ for $\bar{\boldsymbol{X}}$, $\bar{Y}_t$, $\boldsymbol{c}_{T+1,t}$ and $\boldsymbol{c}_{T+1,T+1}$ in equations (99)–(101), one gets the same value of $\hat{\boldsymbol{\psi}}$.

Appendix S7: MVN imputation for mortal-cohort inference

Let $\boldsymbol{\eta}^*_{it}$ denote the value of $\boldsymbol{Y}_{it}$ in the dataset created by MVN imputation with the true values of $\boldsymbol{\mu}$ and $\boldsymbol{\Sigma}$. (Note that with the true values of $\boldsymbol{\mu}$ and $\boldsymbol{\Sigma}$, uMVN imputation and aMVN imputation are the same.) If $\boldsymbol{\epsilon}_{it}$ is normally distributed, then $(\boldsymbol{Y}_2^\top, \ldots, \boldsymbol{Y}_T^\top)^\top$ is multivariate normally distributed given $\boldsymbol{X}$ and $\boldsymbol{Y}_1$, and hence

$$\begin{aligned}
\boldsymbol{\eta}^*_t = \boldsymbol{\eta}^*_t(\boldsymbol{G}_T, \mathcal{R}_{0,T}) &= \begin{cases} \boldsymbol{Y}_t & \text{if} \quad R_{0,t} = 1 \\ E(\boldsymbol{Y}_t \mid \boldsymbol{G}_T) & \text{if} \quad R_{0,t} = 0 \end{cases} \\
&= \begin{cases} \boldsymbol{Y}_t & \text{if} \quad R_{0,t} = 1 \\ E(\boldsymbol{Y}_t \mid \boldsymbol{X}, \boldsymbol{Y}_U, \boldsymbol{Y}_V) & \text{if} \quad R_{0,t} = 0 \end{cases}
\end{aligned}$$

where U and V are, respectively, the last time before t and first time after t that the outcome is observed (so $R_{0,U} = R_{0,V} = 1$ and $R_{0,U+1} = \ldots = R_{0,V-1} = 0$). If the outcome is not observed after t, then $V = T+1$ and $\boldsymbol{Y}_V$ is null. Now,

$$\begin{aligned}
&E(\boldsymbol{\eta}^*_t \mid \boldsymbol{X}, D \geq t) \\
&= P(R_{0,t} = 1 \mid \boldsymbol{X}, D \geq t) E(\boldsymbol{Y}_t \mid \boldsymbol{X}, R_{0,t} = 1, D \geq t) \\
&\quad + P(R_{0,t} = 0 \mid \boldsymbol{X}, D \geq t) E\{\boldsymbol{\eta}^*_t(\boldsymbol{G}_T, \mathcal{R}_{0,T}) \mid \boldsymbol{X}, R_{0,t} = 0, D \geq t\} \\
&= P(R_{0,t} = 1 \mid \boldsymbol{X}, D \geq t) E(\boldsymbol{Y}_t \mid \boldsymbol{X}, R_{0,t} = 1, D \geq t) \\
&\quad + P(R_{0,t} = 0 \mid \boldsymbol{X}, D \geq t) E\{E(\boldsymbol{Y}_t \mid \boldsymbol{X}, \boldsymbol{Y}_U, \boldsymbol{Y}_V) \mid \boldsymbol{X}, R_{0,t} = 0, D \geq t\} \quad (102)
\end{aligned}$$

where $E\{E(\boldsymbol{Y}_t \mid \boldsymbol{X}, \boldsymbol{Y}_U, \boldsymbol{Y}_V) \mid \boldsymbol{X}, R_{0,t} = 0, D \geq t\}$ means $E_{U,V,\boldsymbol{Y}_U,\boldsymbol{Y}_V}\{E(\boldsymbol{Y}_t \mid \boldsymbol{X}, \boldsymbol{Y}_U, \boldsymbol{Y}_V) \mid \boldsymbol{X}, R_{0,t} = 0, D \geq t\}$.

From parts b) and c) of Theorem 10, we have [noting that conditioning on $\{R_{0,U} = 1, R_{0,U+1} = \ldots = R_{0,V-1} = 0, R_{0,V} = 1\}$ means the same as conditioning on

$(U, V, \{R_{0,t} = 0\})$, which means the same as conditioning on (U, V)] that

$$
\begin{aligned}
& E\{E(\boldsymbol{Y}_t \mid \boldsymbol{X}, \boldsymbol{Y}_U, \boldsymbol{Y}_V) \mid \boldsymbol{X}, R_{0,t} = 0, D \geq t\} \\
&= E_{U,V}[E_{\boldsymbol{Y}_U,\boldsymbol{Y}_V}\{E(\boldsymbol{Y}_t \mid \boldsymbol{X}, \boldsymbol{Y}_U, \boldsymbol{Y}_V) \mid \boldsymbol{X}, R_{0,t} = 0, D \geq t, U, V\} \mid \boldsymbol{X}, R_{0,t} = 0, D \geq t] \\
&= E_{U,V}[E_{\boldsymbol{Y}_U,\boldsymbol{Y}_V}\{E(\boldsymbol{Y}_t \mid \boldsymbol{X}, \boldsymbol{Y}_U, \boldsymbol{Y}_V, U, V, R_{0,t} = 0, D \geq t) \mid \boldsymbol{X}, R_{0,t} = 0, D \geq t, U, V\} \\
&\qquad\qquad \mid \boldsymbol{X}, R_{0,t} = 0, D \geq t] \\
&= E_{U,V}\{E(\boldsymbol{Y}_t \mid \boldsymbol{X}, U, V, R_{0,t} = 0, D \geq t) \mid \boldsymbol{X}, R_{0,t} = 0, D \geq t\} \\
&= E(\boldsymbol{Y}_t \mid \boldsymbol{X}, R_{0,t} = 0, D \geq t)
\end{aligned}
$$

and so equation (102) implies that

$$
\begin{aligned}
E(\boldsymbol{\eta}^*_t \mid \boldsymbol{X}, D \geq t) &= P(R_{0,t} = 1 \mid \boldsymbol{X}, D \geq t)E(\boldsymbol{Y}_t \mid \boldsymbol{X}, R_{0,t} = 1, D \geq t) \\
&\quad + P(R_{0,t} = 0 \mid \boldsymbol{X}, D \geq t)E(\boldsymbol{Y}_t \mid \boldsymbol{X}, R_{0,t} = 0, D \geq t) \\
&= E(\boldsymbol{Y}_t \mid \boldsymbol{X}, D \geq t)
\end{aligned}
$$

as required.

If $\boldsymbol{\epsilon}_{it}$ is not normally distributed, formula (18) will not correspond, in general, to the conditional expectation of $\boldsymbol{Y}_t$ given $\boldsymbol{G}_T$ when $R_l = 1$ for some $l > t$. Nevertheless, multiple imputation using the unstructured MVN model has been found often to work well in practice when data are MAR even when not normally distributed (Schafer, 1997; Schafer and Graham, 2002; Lee and Carlin, 2010; Demirtas et al., 2008), and so there is cause to think that uMVN imputation may also work well in practice.

Appendix S8: Further results from Simulation Studies 1 and 2, and Simulation Study 3

Tables S1 and S2 show the results of fitting the linear regression model in simulation studies 1 and 2, respectively, of Section 6 of our paper.

For Simulation Study 1 we also modified the return mechanism so that the independent return assumption was violated. In particular, logit$\{P(R_{0,t} = 1 \mid$

$R_{0,t-1} = 0, \mathcal{R}_{0,t-2}, \mathcal{F}_T)\} = \phi_t + X + (Y_{t-1} + Y_{t-2})/2$, where ϕ_t is chosen to make $P(R_{0,t} = 1 \mid R_{0,t-1} = 0) = 0.5$. The results are shown in Tables S3 and S4.

In Simulation Study 3, data were generated from the same model as in Simulation Study 1 except that $\beta_t = 1.2$ was replaced with $\beta_t = 1$, and independent measurement error e_{it} was added to the underlying outcomes. The errors e_{it} were generated from the same bimodal distribution as ϵ_{it}. The ω_t and ϕ_t values were again chosen so that $P(R_{0,t} = 0 \mid R_{0,t-1} = 1) = 0.5$ and $P(R_{0,t} = 1 \mid R_{0,t-1} = 0) = 0.5$. For each of 1000 simulated datasets we applied the same methods as in Section 6.1. We additionally applied these methods constraining $\beta_t = 1$. Table S5 and Table S6 show the means and empirical SEs of the estimators of μ_t and $(\psi_0, \psi_1, \psi_2, \psi_3)$, respectively. As expected, the methods that constrain $\beta_t = 1$ are approximately unbiased and the methods that do not impose this constraint are biased. LI-LS imputation is more efficient than estimating the compensator, and LI-rMVN imputation is yet more efficient. There is little gain from using rMVN imputation compared to using LI-rMVN imputation.

Appendix S9: Software for LI methods

The LI-LS imputation method can be applied using the FLIM package in R (Hoff, 2014). The other methods (estimating the compensator and the MVN methods), as well as LI-LS imputation, can be applied in R using the *linearincrements()* function available from the MRC Biostatistics Unit website (www.mrc-bsu.cam.ac.uk). An example dataset and R code for analysing it using the FLIM package and the *linearincrements()* function are also provided there.

References

H Demirtas, SA Freels, and RM Yucel. Plausibility of multivariate normality assumption when multiply imputing non-gaussian continuous outcomes: a simulation

assessment. Journal of Statistical Computation and Simulation, 78:69–84, 2008.

KJ Lee and JB Carlin. Multiple imputation for missing data: fully conditional specification versus multivariate normal imputation. American Journal of Epidemiology, 171:624–632, 2010.

R Hoff, JM Gran JM, and D Farewell. Farewell's Linear Increments Model for Missing Data: The FLIM package. The R Journal, 6:137–150, 2014.

JL Schafer and JW Graham. Missing data: Our view of the start of the art. Psychological Methods, 7:147–177, 2002.

LA Stefanski and DD Boos. The calculus of M-estimation. American Statistician, 56:29–38, 2000.

Method	ψ_0	ψ_1	ψ_2	ψ_3
true values	-0.169	0.243	0.592	0.886
	Means			
complete data	-0.164	0.234	0.589	0.887
complete cases	-0.171	0.261	1.153	0.708
LI-LS impute	-0.166	0.237	0.590	0.888
LI-uMVN impute	-0.165	0.235	0.589	0.887
LI-aMVN impute	-0.164	0.235	0.589	0.888
uMVN impute	-0.164	0.233	0.590	0.887
aMVN impute	-0.163	0.233	0.589	0.887
	Empirical SEs			
complete data	0.104	0.184	0.093	0.162
complete cases	0.122	0.207	0.122	0.193
LI-LS impute	0.167	0.255	0.136	0.196
LI-uMVN impute	0.135	0.224	0.111	0.181
LI-aMVN impute	0.135	0.223	0.111	0.182
uMVN impute	0.131	0.217	0.111	0.182
aMVN impute	0.131	0.217	0.111	0.182

Table S1: Means and empirical SEs of estimated ψ_0, ψ_1, ψ_2 and ψ_3 in Simulation Study 1 of Section 6

Method	ψ_0	ψ_1	ψ_2	ψ_3
true values	-0.224	0.360	0.684	0.927
	Means			
complete data	-0.223	0.352	0.685	0.924
complete cases	-0.132	0.252	1.052	0.867
LI-LS impute	-0.213	0.338	0.685	0.922
LI-uMVN impute	-0.218	0.343	0.684	0.924
uMVN impute	-0.220	0.347	0.684	0.923
	Empirical SEs			
complete data	0.158	0.267	0.099	0.170
complete cases	0.173	0.291	0.140	0.225
LI-LS impute	0.233	0.357	0.144	0.217
LI-uMVN impute	0.197	0.319	0.121	0.196
uMVN impute	0.192	0.309	0.121	0.196

Table S2: Means and empirical SEs of estimated ψ_0, ψ_1, ψ_2 and ψ_3 in Simulation Study 2 of Section 6

Method	μ_1	μ_2	μ_3	μ_4	μ_5	μ_6
true values	0.250	0.950	1.790	2.798	4.008	5.459
	Means					
complete data	0.249	0.945	1.784	2.790	3.998	5.447
complete cases	0.249	1.482	2.803	4.560	6.415	8.700
estim. compens.	0.249	0.944	1.784	2.787	3.998	5.446
LI-LS impute	0.249	0.944	1.898	3.059	4.414	6.009
LI-uMVN impute	0.249	0.991	2.069	3.293	4.672	6.298
LI-aMVN impute	0.249	0.999	2.067	3.291	4.676	6.295
uMVN impute	0.249	1.009	2.065	3.265	4.656	6.304
aMVN impute	0.249	1.018	2.062	3.259	4.655	6.295
	Empirical SEs					
complete data	0.045	0.084	0.120	0.160	0.204	0.255
complete cases	0.045	0.114	0.160	0.195	0.250	0.294
estim. compens.	0.045	0.113	0.227	0.350	0.491	0.655
LI-LS impute	0.045	0.113	0.180	0.236	0.294	0.356
LI-uMVN impute	0.045	0.103	0.136	0.166	0.208	0.255
LI-aMVN impute	0.045	0.100	0.135	0.165	0.207	0.254
uMVN impute	0.045	0.101	0.134	0.161	0.205	0.254
aMVN impute	0.045	0.098	0.133	0.161	0.204	0.254

Table S3: Means and empirical SEs of estimated μ_t in Simulation Study 1 when return mechanism is modified to violate independent return assumption.

Method	ψ_0	ψ_1	ψ_2	ψ_3
true values	-0.169	0.243	0.592	0.886
	Means			
complete data	-0.164	0.234	0.589	0.887
complete cases	-0.164	0.275	1.344	0.533
LI-LS impute	-0.289	0.335	0.774	0.758
LI-uMVN impute	-0.271	0.325	0.885	0.658
LI-aMVN impute	-0.267	0.324	0.884	0.659
uMVN impute	-0.252	0.294	0.883	0.657
aMVN impute	-0.247	0.293	0.879	0.659
	Empirical SEs			
complete data	0.104	0.184	0.093	0.162
complete cases	0.115	0.201	0.107	0.174
LI-LS impute	0.168	0.254	0.138	0.194
LI-uMVN impute	0.134	0.223	0.099	0.165
LI-aMVN impute	0.133	0.223	0.099	0.166
uMVN impute	0.131	0.217	0.098	0.164
aMVN impute	0.130	0.217	0.099	0.165

Table S4: Means and empirical SEs of estimated ψ_0, ψ_1, ψ_2 and ψ_3 in Simulation Study 1 when return mechanism is modified to violate independent return assumption.

Method	μ_1	μ_2	μ_3	μ_4	μ_5	μ_6
true values	0.250	0.900	1.550	2.200	2.850	3.500
	Means					
complete data	0.251	0.897	1.544	2.193	2.846	3.496
complete cases	0.249	1.346	2.065	3.087	4.027	4.933
unconstrained β_t						
estim. compens.	0.251	1.186	2.251	3.298	4.427	5.517
LI-LS impute	0.251	1.186	2.033	2.872	3.748	4.565
LI-uMVN impute	0.251	1.132	1.726	2.457	3.162	3.898
LI-aMVN impute	0.251	1.111	1.715	2.436	3.149	3.956
uMVN impute	0.251	1.085	1.658	2.363	3.049	3.809
aMVN impute	0.251	1.060	1.651	2.355	3.065	3.956
constrained $\beta_t = 1$						
estim. compens.	0.251	0.891	1.545	2.181	2.844	3.491
LI-LS impute	0.251	0.891	1.547	2.182	2.848	3.496
LI-rMVN impute	0.251	0.892	1.548	2.186	2.853	3.499
rMVN impute	0.251	0.893	1.547	2.188	2.854	3.499
	Empirical SEs					
complete data	0.078	0.098	0.121	0.139	0.156	0.168
complete cases	0.045	0.107	0.146	0.175	0.189	0.205
unconstrained β_t						
estim. compens.	0.078	0.139	0.243	0.295	0.319	0.339
LI-LS impute	0.078	0.139	0.193	0.226	0.252	0.261
LI-uMVN impute	0.078	0.134	0.159	0.186	0.204	0.219
LI-aMVN impute	0.078	0.134	0.159	0.185	0.203	0.217
uMVN impute	0.078	0.133	0.158	0.185	0.205	0.222
aMVN impute	0.078	0.132	0.157	0.181	0.202	0.217
constrained $\beta_t = 1$						
estim. compens.	0.078	0.148	0.248	0.315	0.363	0.406
LI-LS impute	0.078	0.148	0.193	0.217	0.234	0.244
LI-rMVN impute	0.078	0.138	0.161	0.182	0.200	0.215
rMVN impute	0.078	0.135	0.159	0.178	0.199	0.215

Table S5: Means and empirical SEs of estimated μ_t in Simulation Study 3. Estimating the compensator and LI-LS imputation are applied both with β_t estimated and with β_t constrained to equal 1.

Method	ψ_0	ψ_1	ψ_2	ψ_3
true values	0.000	0.500	0.400	0.500
	Means			
complete data	0.004	0.486	0.397	0.503
complete cases	0.036	0.479	0.697	0.417
unconstrained β_t				
LI-LS impute	0.040	0.503	0.707	0.306
LI-uMVN impute	0.081	0.465	0.481	0.469
LI-aMVN impute	0.051	0.481	0.497	0.455
uMVN impute	0.067	0.452	0.460	0.473
aMVN impute	0.018	0.470	0.495	0.453
constrained $\beta_t = 1$				
LI-LS impute	0.008	0.474	0.394	0.511
LI-rMVN impute	0.007	0.477	0.397	0.506
rMVN impute	0.008	0.475	0.397	0.506
	Empirical SEs			
complete data	0.116	0.205	0.059	0.103
complete cases	0.157	0.273	0.087	0.139
unconstrained β_t				
LI-LS impute	0.222	0.350	0.107	0.154
LI-uMVN impute	0.186	0.312	0.084	0.136
LI-aMVN impute	0.187	0.315	0.085	0.136
uMVN impute	0.183	0.309	0.084	0.135
aMVN impute	0.185	0.312	0.085	0.136
constrained $\beta_t = 1$				
LI-LS impute	0.228	0.367	0.109	0.166
LI-rMVN impute	0.190	0.320	0.085	0.138
rMVN impute	0.186	0.315	0.084	0.136

Table S6: Means and empirical SEs of estimated ψ_0, ψ_1, ψ_2 and ψ_3 in in Simulation Study 3. LI-LS imputation is applied both with β_t estimated and with β_t constrained to equal 1.
